# Enantiospecific Optical Sensing of Terpenes by an Aggregated Atropisomeric Platinum(II) Complex

**DOI:** 10.1002/anie.202523522

**Published:** 2026-01-27

**Authors:** Annika Huber, Alessandro Prescimone, Christof Sparr, Oliver S. Wenger

**Affiliations:** ^1^ Department of Chemistry University of Basel St. Johanns‐Ring 19 Basel 4056 Switzerland

**Keywords:** 1,2‐naphthylenes, aggregation, metal‐metal interactions, nanostructures, UV–vis spectroscopy

## Abstract

Terpenes are unfunctionalized small volatile organic compounds (VOCs) that are naturally abundant, relevant to climate change, and impose potential health risks. Herein, we report a conceptually novel approach for the enantiospecific recognition of terpenes and other VOCs based on a square planar platinum(II) complex with an atropisomeric ligand. This molecular design results in chiral aggregated nanostructures caused by weak π‐π‐ and metal–metal interactions with characteristic UV–vis absorption bands. Distinct UV–vis absorption changes are induced by weak intermolecular interactions with the enantiomers of diverse VOCs (2‐butanol, 1‐phenylethanol, α‐pinene, and limonene) leading to perturbations of the chiral aggregates. This allows an enantiospecific, reversible detection of VOCs by UV–vis absorption spectroscopy in nonpolar solution. The study provides a new working principle for enantiospecific recognition with artificial optoelectronic noses, which are particularly promising for determining the enantiomers of unfunctionalized volatile organics.

## Graphical abstract

Aggregated metal complexes act as an artificial nose and permit reversible optical sensing of small volatile molecules like *R*/*S*‐limonene based on enantiospecific recognition.

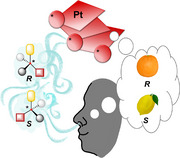

## Introduction

While chemical sensing advanced to a sophisticated technology, the enantiospecific recognition and optoelectronic discrimination of enantiomers, characterized by their mirror image relationship, remains a formidable challenge.^[^ ^1^, ^2^, ^3^, ^4^, ^5^, ^6^ ^]^ This applies to many different volatile organic compounds (VOCs), where terpene hydrocarbons pose a specific obstacle due to the lack of heteroatom containing functional groups. Terpenes are relevant on a global scale due to their impact on climate change,^[^ ^7^, ^8^, ^9^ ^]^ and can even pose a risk to human health.^[^ ^10^, ^11^, ^12^ ^]^ Enantioselective vapochromism or vapoluminescence are thus highly desirable to be achieved based on molecular properties leading to sensing materials. The human nose readily discriminates between enantiomers of numerous VOCs, for example *R‐*limonene is perceived by an orange‐, whereas *S*‐limonene is recognized as turpentine or lemon odor.^[^ ^13^ ^]^ In artificial systems however, the fundamental working principles of enantiomer recognition of VOCs are underexplored.^[^ ^3^, ^14^, ^15^, ^16^, ^17^ ^]^ The aim of our study was therefore to develop a concept for the enantiospecific sensing of VOCs with chiral nanostructured molecular materials by optoelectronic discrimination between different enantiomers.

Identification and quantification of VOC concentrations in gaseous mixtures has been realized mostly through physical measurement techniques. Among the applied methods are photo‐ionization detectors, semiconductor‐ or amperometric sensors, and gas chromatography (GC).^[^ ^18^, ^19^ ^]^ For the enantiospecific recognition of VOCs by GC, specialized chiral columns were developed, utilizing chiral stationary phases based on cyclodextrins, metal‐organic frameworks, or amino acids.^[^ ^20^, ^21^ ^]^ The established analytical methods that are able to distinguish enantiomers, such as GC combined with mass spectrometry,^[^ ^22^, ^23^ ^]^ often require laborious sample preparation (i.e., adsorbing gaseous samples on an adsorbate for later extraction) and sophisticated technical equipment.^[^ ^14^, ^24^, ^25^, ^26^ ^]^ A simple color or luminescence change by a chemical sensor could be a more accessible way of detecting different enantiomers.^[^ ^17^ ^]^ Changes in crystal packing or aggregation induced by analyte molecules thereby lead to alterations of the weak intermolecular interactions of molecular materials.^[^ ^27^ ^]^ These structural perturbations can be exploited to induce color or luminescence changes.^[^ ^28^, ^29^, ^30^, ^31^, ^32^ ^]^ Enantiospecific recognition utilizing this methodology has so far been accomplished with crystalline vapochromic sensors^[^ ^33^ ^]^ and recently also by sensors that specially reacted with a functional group of the sensor, thereby specifying and limiting the analyte`s functional groups.^[^ ^16^, ^17^ ^]^ A stereogenic organic sensor molecule showed enantiospecific color changes upon charge‐aided hydrogen bonding with carboxylic acids.^[^ ^17^ ^]^ Discrimination of seven amine enantiomer pairs was achieved by a condensation reaction with a formyl‐containing Pt(II)‐based sensor reaching a limit of detection (LOD) of 0.52 × 10^−6^ M with 0.41 × 10^−6^ M sensor.^[^ ^16^ ^]^

Sensing materials that distinguish enantiomers thus remained highly analyte‐specific. Broadly applicable molecular sensors, especially for analytes without polar functional groups, are of high interest.^[^ ^15^, ^21^, ^31^, ^33^ ^]^ Enantiospecific recognition of a particular analyte has been shown to arise from a configuration‐dependent interaction on the individual molecular level or from the perturbation of a defined supramolecular packing.^[^ ^16^, ^17^, ^34^, ^35^ ^]^ Stereoisomers of VOC analytes thereby interact with the aggregated transition‐metal complexes by direct coordination, strong binding (covalent‐ or hydrogen bonds), intercalation, or through π‐π interactions with ligands of a defined stereo‐configuration.^[^ ^16^, ^17^, ^33^, ^36^, ^37^ ^]^ We envisioned a nanostructured material that can sense enantiomers of diverse VOC analytes by utilizing weak intermolecular interactions for the recognition at various regimes, ideally including substrates without polar functional groups.

Aggregated square‐planar Pt(II) complexes emerged as ideal sensors, because they tend to stack on top of each other as a result of weak attractive forces between adjacent d^8^ metals.^[^ ^16^, ^27^, ^28^, ^35^, ^38^, ^39^, ^40^, ^41^ ^]^ In combination with chelate ligands possessing extended π‐conjugated frameworks, these metal‐metal interactions lead to electronic transitions of the metal–metal‐to‐ligand charge transfer (MMLCT) type, giving rise to characteristic UV–vis absorption bands.^[^ ^39^, ^42^ ^]^ Perturbation of the Pt(II) stacks by an analyte can alter the metal‐metal interaction as well as the π‐π interactions and lead to color changes.^[^ ^28^, ^29^, ^32^, ^43^, ^44^, ^45^ ^]^ To enable enantiospecific sensing, the incorporation of stereogenic elements into the Pt(II) complex structures was anticipated. Enantiospecific sensing by weak intermolecular interactions was previously achieved with a platinum(II) double salt, capable of recognizing the two enantiomers of 2‐butanol (2‐BuOH) in the solid state.^[^ ^33^ ^]^ Upon penetration of the crystalline *R*‐ and *S*‐enantiomers of the Pt(II) double salt, *R*‐ or *S*‐2‐BuOH vapors induced distinct shifts in the photoluminescence band maxima. Since exposure to vapors was not quantified, no LOD was reported. In the context of light emitting devices, anti‐counterfeiting applications, and supramolecular systems, stacked square planar Pt(II) complexes with stereogenic elements have been reported to provide access to helical frameworks with unique aggregation behavior.^[^ ^46^, ^47^, ^48^ ^]^ Many reports describe charged complexes. The stereogenic Pt(II) complex presented in this work was specifically designed to be charge‐neutral to allow for intermolecular interactions with neutral small molecules.

Furthermore, for chiroptical luminophores, charge‐neutral Pt(II) complexes were used to achieve chiral aggregates.^[^ ^49^ ^]^ The complexes were bearing a ligand equipped with mandelic acid to introduce a stereocenter. Similar *P*‐ and *M*‐helical nanostructure aggregates were also obtained with Pt(II) complexes bearing *R*‐ or *S*‐isocyanide ligands.^[^ ^50^ ^]^ These previous reports indicated the viability of controlled chiral nanostructures from aggregated, configurationally defined Pt(II) complexes as a design strategy for enantiospecific sensing.^[^ ^49^, ^50^, ^51^, ^52^, ^53^, ^54^, ^55^, ^56^ ^]^

Accordingly, charge‐neutral Pt(II) complexes were expected to stack without interfering effects caused by counterions and bis‐anionic tridentate triazolylpyridine (trzpy) ligand seemed ideal for this purpose.^[^ ^49^ ^]^ To achieve the desired helical stacking, we further considered introducing the necessary stereogenic element in the form of an atropisomeric monodentate ligand, completing the coordination sphere around Pt(II). To the best of our knowledge, the well‐defined 3D topology of atropisomers has not yet been integrated into nanostructured Pt(II) based sensor materials. A configurationally stable, atropisomeric 1,2′‐binaphthyl moiety at the terminus of a monodentate arylisocyanide ligand appeared to be well‐suited as stereogenic element that can allow for enantiospecific CH–π‐ and π–π interactions with analyte molecules.^[^ ^57^, ^58^, ^59^, ^60^, ^61^ ^]^ Furthermore, the arylisocyanide unit of the ligand allows the sterically demanding atropisomeric moieties to be placed far enough from the Pt(II) metal to still allow suitable metal‐metal contacts and the required stacking for a controlled aggregation.^[^ ^62^, ^63^, ^64^ ^]^ Overall, the notion of an enantiospecific sensing based on an atropisomeric coordination complex was examined.

## Results and Discussion

### Specific Molecular Design Principles

**Figure 1:**
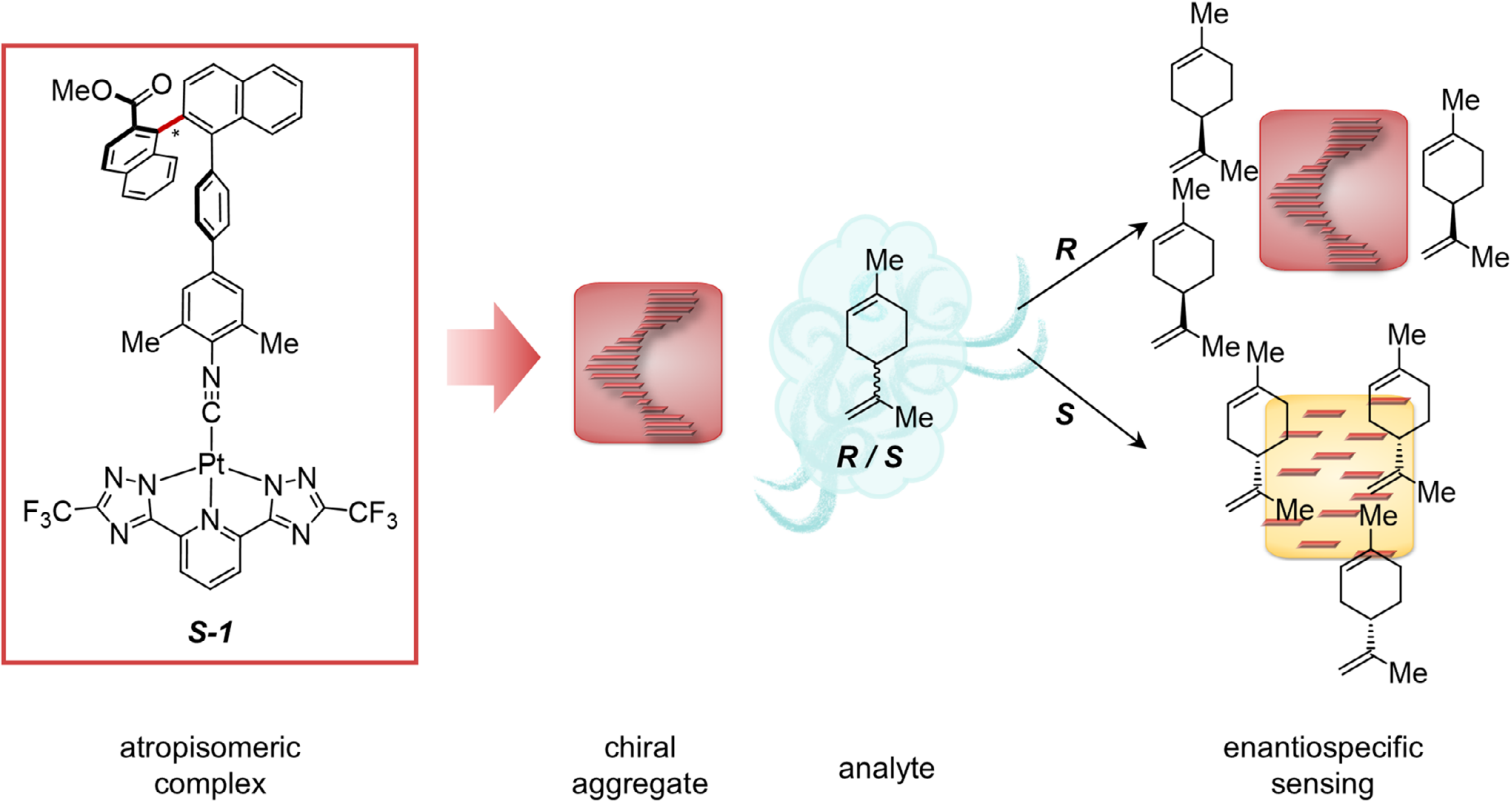
From left to right: molecular structure of ***S*‐1** and a schematic representation of aggregated ***S*‐1**, forming the “chiral nose” type material. Aggregated ***S*‐1** was anticipated to show an enantiospecific response toward VOC enantiomers (indicated by *R*/*S*‐limonene). The aggregated structure could either be preserved (indicated by the red color and persistent stacking) or perturbed and lead to a different color (indicated in yellow).

After the specific ligand design was developed by molecular modelling (Figures S1–S3), the isocyanide ligand was installed on a biphenyl unit at the 4‐position that was equipped with a configurationally stable binaphthyl‐unit at the 4′‐position. Two methyl groups were placed at the *ortho*‐positions next to the ligand for increased stability of the metal complex (Figure 1), a general design principle for obtaining inert isocyanide complexes.^[^ ^65^, ^66^, ^67^, ^68^ ^]^ To allow the formation of molecular stacks from Pt(II) isocyanide complexes, steric hindrance in proximity to the metal center was decreased by inserting a phenyl spacer between the binaphthyl‐ and the isocyanide moieties.

In view of the aggregation requirements as well as efficient enantiomer recognition, we reasoned that the end group on the naphthyl‐chain should be balanced with respect to steric repulsion, while still allowing differentiated intermolecular interactions. At the same time, averaging the polarity was key since an unfunctionalized nonpolar system restricts the range of possible molecular interactions with VOCs to CH‐π, π‐π or London dispersion interactions. A methyl ester end group appeared ideal, allowing for polar interactions with potential analytes while introducing only minimal steric hindrance.

### Synthesis of the Atropisomeric Ligand and the Pt(II) Complex

**Scheme 1:**
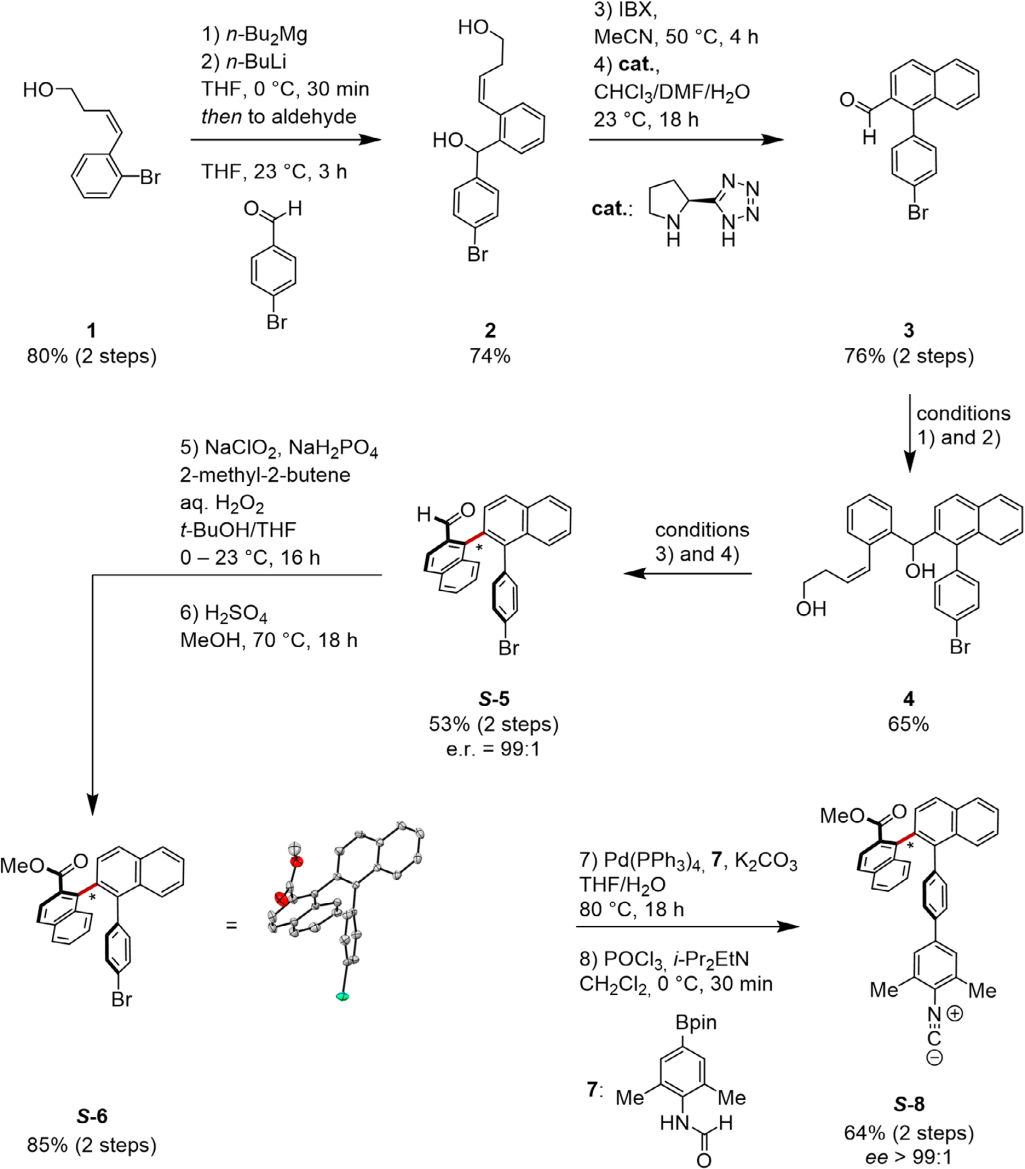
Atroposelective synthesis of aldehyde ***S*‐5**, X‐ray crystal structure of methyl ester ***S*‐6**, and preparation of the isocyanide ligand ***S*‐8**.

For the synthesis of the atropisomeric ligand ***S*‐8**, we implemented the iterative stereoselective arene‐forming aldol‐condensation strategy (Scheme 1).^[^ ^69^, ^70^ ^]^ More specifically, secondary amine organocatalysis allowed to assemble configurationally stable 1,2′‐naphthyl atropisomers with a high level of stereocontrol. The precursor **1** was prepared in 80% yield in two steps and commercially available starting materials enabled the synthesis of Na[Pt(trzpy)Cl] (detailed procedures, characterization, and spectroscopic data are provided in Supporting Information).^[^ ^51^, ^53^, ^69^ ^]^

With 4‐bromobenzaldehyde as the starting material, addition of **1** and the arene‐forming aldol condensation were optimized. The 1,2‐binaphthalene carbaldehyde intermediate ***S*‐5** was obtained with an excellent stereoselectivity of 99:1.

**Scheme 2:**
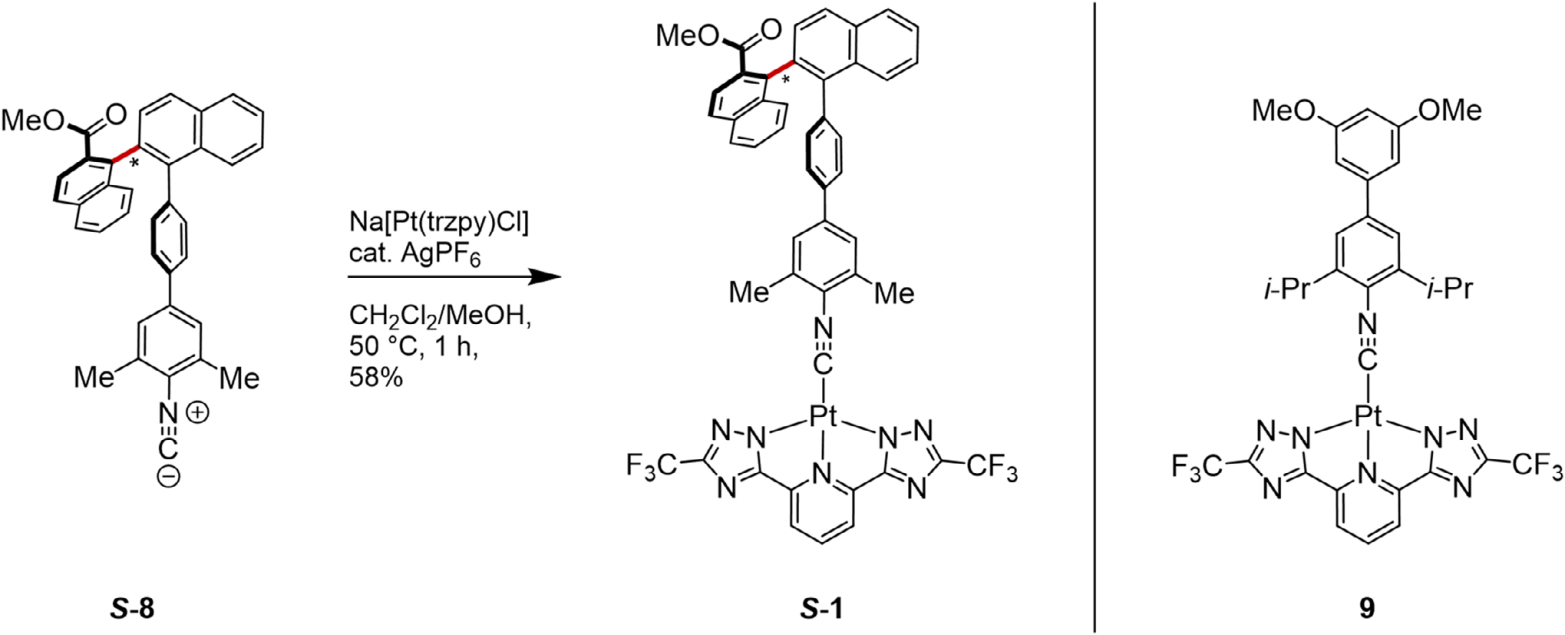
Synthesis of the Pt(II) complex ***S‐*1** and the molecular structure of reference complex **9**.

Aldehyde ***S*‐5** was subjected to Pinnick oxidation followed by esterification, yielding methyl ester ***S*‐6** in 85% yield over two steps. Notably, crystals of ***S*‐6** suitable for X‐ray crystallography confirmed the assigned absolute configuration. Suzuki cross‐coupling of ***S*‐6** and **7** resulted in the formamide ligand precursor and dehydration of the formamide completed the isocyanide ligand synthesis (***S*‐8**), setting the stage for the complexation (Scheme 2).

Reaction of the precursor complex Na[Pt(trzpy)Cl] with ligand ***S*‐8** yielded the complex ***S*‐1** in 58% isolated yield. A catalytic amount of silver hexafluorophosphate thereby facilitated the ligand exchange of the chloride by the isocyanide. For the photophysical investigations, we also prepared the achiral complex **9** with essentially the same coordination sphere using a monodentate isocyanide ligand lacking the atropisomeric unit. Both complexes were characterized by NMR spectroscopy, HRMS, and FTIR spectroscopy (spectroscopic data provided in the Supporting Information).

### Optical Properties of the Pt(II) Complex, Aggregate Formation, and VOC Sensing

**Figure 2:**
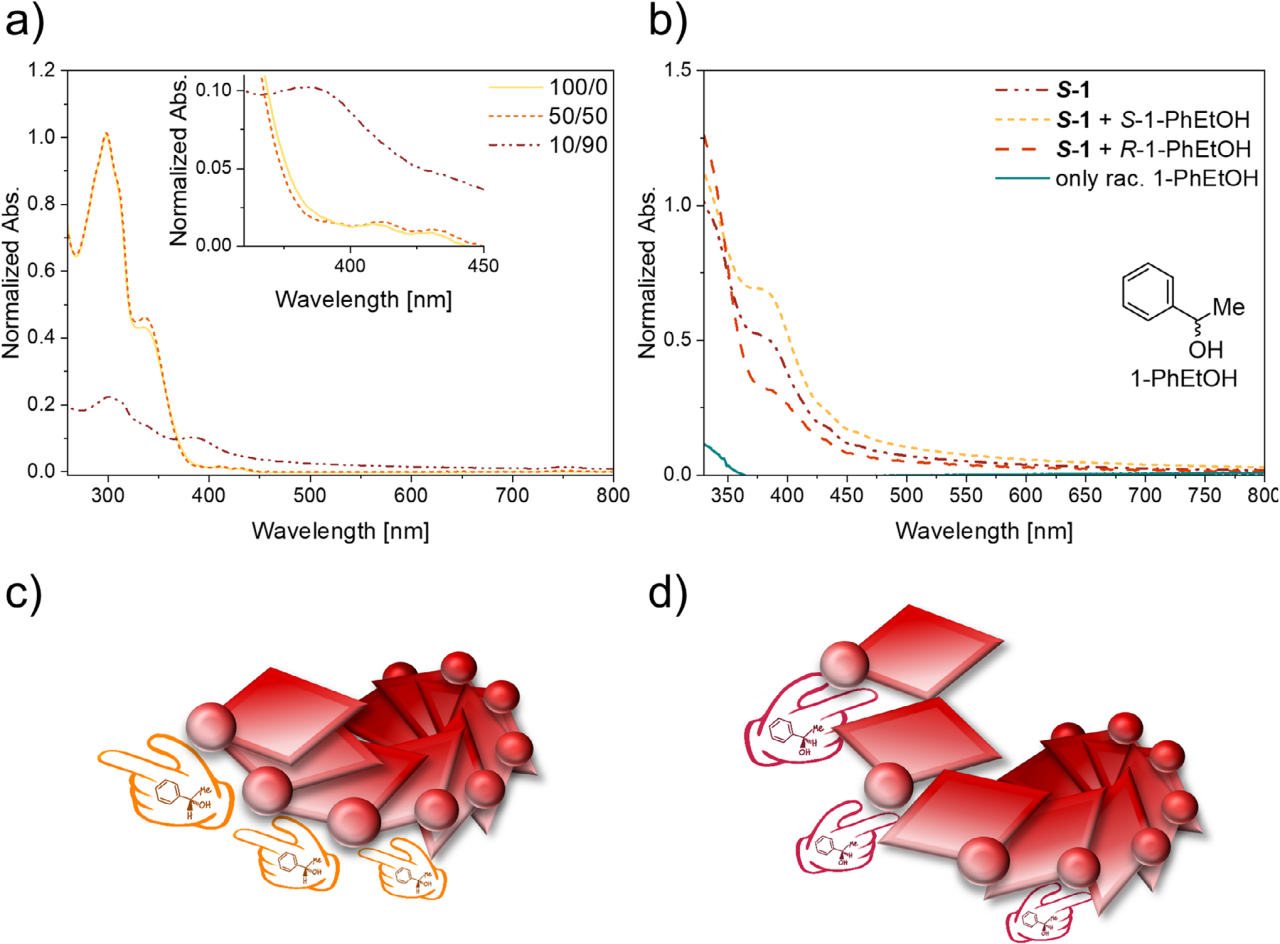
a) UV–vis absorption spectra of ***S*‐1** (10^−5^ M) in neat CHCl_3_ and in CHCl_3_/*n*‐hexane mixtures at the given ratios. The inset highlights the newly formed MMLCT absorption band signaling Pt(II)‐Pt(II) stacking as a result of aggregation in less polar solvent. b) Enantiospecific recognition of *R*‐ and *S*‐1‐PhEtOH (0.42 mmol, 850 eq.). c) Schematic representation of a situation that increased the metal‐metal distances between helically stacked complexes. Complexes arerepresented by the red parallelograms with one point on the corner to symbolize the chiral ligand ***S*‐8**. Left hands represent the *S*‐enantiomers of the analyte. d) Schematic representation of a situation, where the metal‐metal distances are perturbed by analyte interactions (intercalating *R*‐enantiomers represented by right hands).

In CHCl_3_ solution, the complex ***S*‐1** shows intra‐ligand (IL) π‐π* transitions leading to absorption band maxima at 298 nm and 336 nm (solid yellow trace in Figure 2a). The weak and broad absorption bands between 370–450 nm are attributed to metal‐to‐ligand charge transfer (MLCT), possibly with some admixed IL character.^[^ ^53^ ^]^ In an attempt to induce aggregation, we added *n*‐hexane and found that in a 50/50 CH_3_Cl/*n*‐hexane mixture the UV–vis absorption spectrum remains largely unchanged (red dotted trace in Figure 2a). At a 10/90 solvent ratio of CHCl_3_/*n‐*hexane, significant changes in the absorption spectrum became detectable (purple dash‐dotted trace in Figure 2a). At equal concentration of ***S*‐1** as in neat CHCl_3_ (10^−5^ M), the absorption bands below 350 nm dropped in intensity, while a new broad band arose at 385 nm. In this spectral region we anticipate metal–metal‐to‐ligand charge transfer (MMLCT) transitions, enabled by close Pt(II)‐Pt(II) contacts in the formed aggregates (≤ 3.5 Å) along with increased π‐π interactions between aromatic units of neighboring complexes within the aggregate.^[^ ^39^, ^71^, ^72^ ^]^ The baseline increase in the UV–vis absorption spectrum recorded in the 10/90 mixture of CHCl_3_/*n‐*hexane (tailing all the way to 800 nm) is compatible with the formation of aggregates and a colloidal suspension that leads to increased light scattering. Scanning electron microscope (SEM) imaging of drop‐casted ***S*‐1** from a 10/90 mixture of CHCl_3_/*n‐*hexane provided clear evidence for the formation of nanowires between 4 and 12 µm in length and a thickness ranging from 0.20– 0.33 µm (Figure S12). Reference complex **9** did not show such aggregation behavior nor strong spectral changes in the same solvent mixtures. The observed aggregation of complex ***S*‐1** was reversible, which could allow for reversible sensing applications. In all further studies, the 10/90 solvent mixture CHCl_3_/*n‐*hexane was used to obtain aggregates of ***S*‐1** and to study any changes in the optical spectroscopic properties. ***S*‐1** showed only very weak photoluminescence (Figure S5d), making UV–vis absorption spectroscopy the preferred method of detection. Previous work suggests that pronounced photoluminescence changes can be expected mostly in solid‐state emission rather than in solution.^[^ ^27^, ^32^ ^]^

Next, the aggregated ***S*‐1** was investigated for the enantiospecific recognition of selected small molecule enantiomers. For this purpose, ***S*‐1** was tested with the four readily available enantiomer pairs of 2‐butanol, 1‐phenylethanol, α‐pinene, and limonene. Significant changes in the spectral region of the MMLCT absorption band around 385 nm arose upon addition of excess *R*/*S*‐1‐PhEtOH (Figure 2b). Most importantly, the *R‐* and *S‐*enantiomers of 1‐PhEtOH had an opposite effect on the absorbance change, leading to a 37% decrease of the absorbance for *R*‐1‐PhEtOH and a 34% increase for *S*‐1‐PhEtOH. Thus, 1‐PhEtOH indicates the effect of different analyte enantiomers on the complex aggregate (Figure 2c,d) at this complex to analyte ratio (850 eq.). While the *S*‐enantiomer showed further increase of the MMLCT absorption (Figure 2c), the *R*‐enantiomer lowered the absorption (Figure 2d).

It seems plausible that metal‐metal interactions are probed at 385 nm, as well as intense π‐π interactions allowing to postulate a scenario in which the analyte displaces the individual Pt(II)complex units in a lateral direction relative to the overall aggregate, resulting in longer Pt(II)–Pt(II) distances and decreased metal–metal interactions (Figure 2d). In an alternative scenario (Figure 2c), the analyte‐sensor interaction leads to shorter metal‐metal contacts, thus increasing the MMLCT absorption band, and potentially allowing stronger π‐π interactions at the same time.

Aggregated ***S*‐1** also showed an enantiospecific response to 1‐PhEtOH in circular dichroism (CD). The CD signals indicated that the complex arranged into chiral higher ordered structures, which amplified the differential absorption of circularly polarized light (Figure S4c). Upon addition of *R*/*S*‐1‐PhEtOH, analogous effects to the UV–vis absorbance changes in Figure 2 were observed (Figure S4d). While these CD studies are useful to corroborate the findings of the UV–vis investigations, the latter allow a more direct sensitivity assessment of analyte sensing by our aggregates.

**Figure 3:**
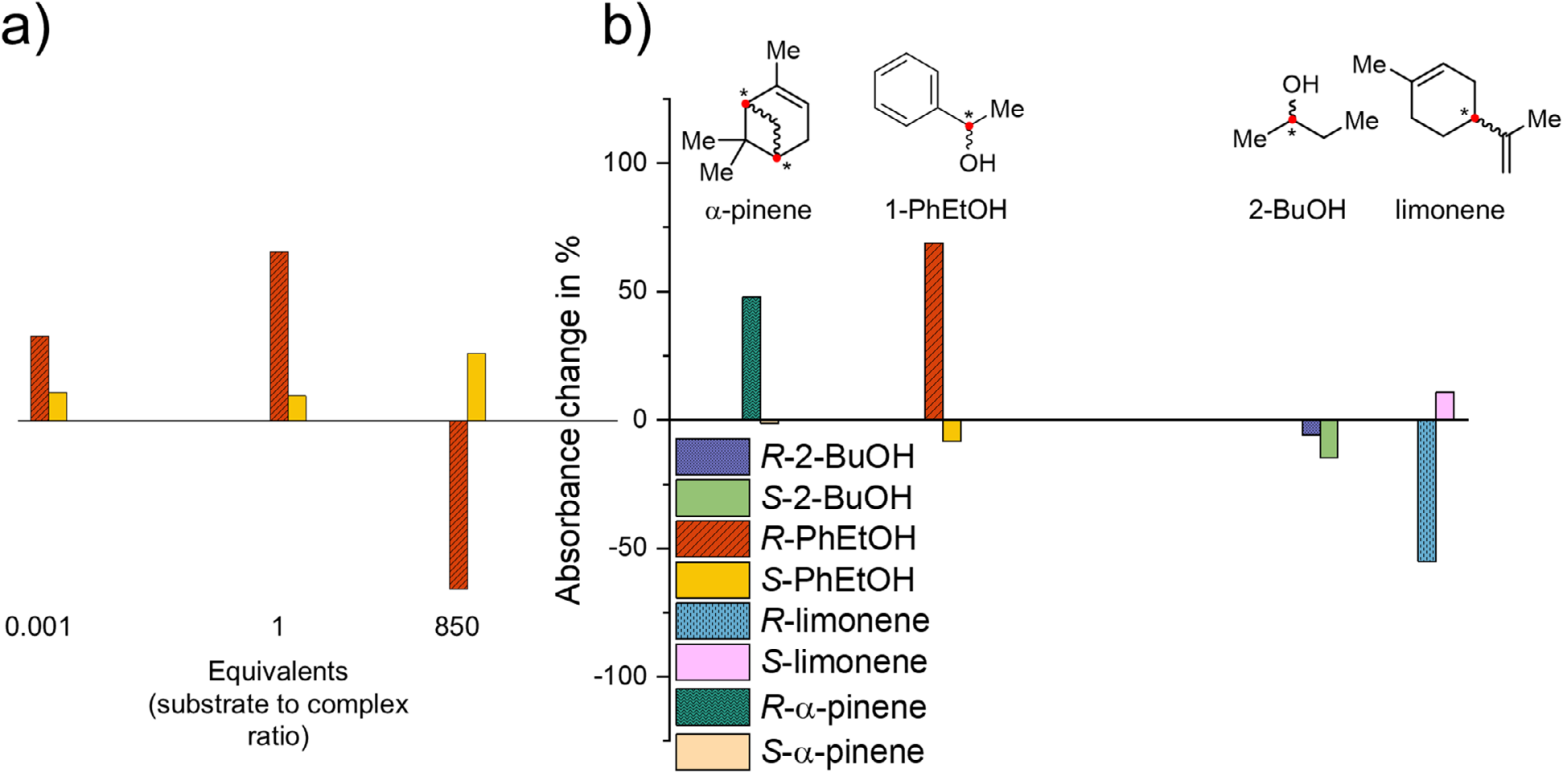
a) Schematic representation of the individual effects of *R*/*S* 1‐PhEtOH on aggregated ***S*‐1** depending on the added equivalents, showcasing the sensor sensitivity. Equivalents are given with respect to the concentration of monomeric ***S*‐1** used to prepare the aggregate. b) Effects of the different analyte enantiomers on aggregated ***S*‐1** with the corresponding molecular structures displayed on top. Pure aggregated ***S*‐1** corresponds to an absorbance change of 0%. The analyte to sensor ratios were: 2‐BuOH 1800 eq., 1‐PhEtOH 9 eq., limonene 3000 eq., α‐pinene 0.002 eq. The absorbance change at 392 nm was monitored.

Depending on the amount added with respect to the nominal ***S*‐1** concentration, the two 1‐PhEtOH enantiomers showed different effects on the measured absorbance at 392 nm (Figure 3). At stoichiometric amounts of analyte and lower analyte‐to‐***S*‐1** ratios, the effect on the absorbance change was reversed compared to the situation with an excess of 1‐PhEtOH (Figure 2b, 850 eq.). When stoichiometric or sub‐stoichiometric amounts of analyte are present, both enantiomers of 1‐PhEtOH cause an increase in absorbance at 392 nm. Gratifyingly, differentiation of the enantiomers by means of aggregated ***S*‐1** was feasible at low analyte ratios due to the distinct intensity changes caused by *R*‐ and *S*‐1‐PhEtOH.

Aggregated ***S*‐1** in the 10/90 CH_3_Cl/*n*‐hexane mixture is furthermore capable of enantiospecific recognition of 2‐BuOH, α‐pinene, and limonene (Figure 3b and Figure S6). Like 1‐PhEtOH, the enantiomers of the aliphatic 2‐BuOH showed effects on the MMLCT absorption band of aggregated ***S*‐1** (Figure S6a). Here, the effects caused by R/S enantiomers followed a consistent trend over a wide quantitative range (0.001 – 1`000 eq.) with distinct differences starting from >1 eq. (Figure S7a). Moving to more challenging analytes with further reduced possible intermolecular interactions, the aliphatic terpenes α‐pinene and limonene were chosen as representative environmentally relevant VOCs. ***S*‐1** showed enantiospecific recognition for both, α‐pinene and limonene (Figure 3b and Figure S6). Comparable to *R*/*S*‐PhEtOH, both VOCs showed switching trends between the *R*‐ and *S*‐ enantiomer`s effects on ***S*‐1** aggregation (Figure S8). Notably, aggregated ***S*‐1** was able to differentiate all enantiomers even at sub‐stoichiometric amounts. Therefore, the upper and lower LODs were explored in enantiomer titration experiments. The upper saturation limit was not reached at 5`100 eq. for any of the tested enantiomers (0.10 – 0.18 M). At the lower limit, most enantiomers could be differentiated at 0.01 eq. or even less (the lowest being 2.4 × 10^−8^ M for limonene and α‐pinene). Over the course of our studies, ***S*‐1** proved stable over multiple measurements and could be regenerated at least four times by a particularly simple washing procedure with *n*‐pentane (Supporting Information, Section 1.2 and Section 6.2).

## Conclusions

A new concept for the enantiospecific detection of environmentally relevant VOC analytes was developed. By integrating an atropisomeric organic ligand into a square‐planar Pt(II) complex, aggregated supramolecular structures resulting from the stacking of the individual Pt(II) complexes become sensitive to chiral analytes. Different enantiomers of the same analyte interfere differently with the metal‐metal interactions between stacked chiral complexes, resulting in enantiospecific changes in the UV–vis absorption spectrum. The supramolecular aggregation is reversible and can be controlled by solvent polarity. The aggregates discriminate between the different enantiomers of selected alcohol and terpene analytes. Notably, the enantiospecific detection of nonpolar hydrocarbon molecules with C═C double bonds as the only functional groups generally poses an immense challenge compared to the detection of polar analytes with heteroatom‐containing functional groups. Many previously reported concepts for supramolecular aggregation rely on intermolecular interactions between heteroatom‐containing functional groups that are highly polar or even charged, and consequently perturbation of such aggregation is often limited. The approach reported herein is based on a charge‐neutral complex in which metal‐metal interactions contribute to stacking and aggregation, while the atropisomeric ligands provide enantiospecificity. This could be a generally applicable design principle for highly sensitive enantiospecific VOC sensing. Possible applications demonstrate relevance not only to research on climate change, but also to monitor work‐place air quality or for fragrance and odor nuisance applications.

## Conflict of Interests

The authors declare no conflict of interest.

## Supporting information



Supporting Information

## Data Availability

The data that support the findings of this study are available in the Supporting Information of this article.
