# Supplementary material for: Enantiospecific Optical Sensing of Terpenes by an Aggregated Atropisomeric Platinum(II) Complex
Source: Angew Chem Int Ed Engl. 2026 Jan 27;65(10):e23522. doi: 10.1002/anie.202523522 (PMC12955516; doi:10.1002/anie.202523522)
Supplement: Supplementary file 1 — Supporting Information [file ANIE-65-e23522-s001.docx]

Enantiospecific optical sensing of terpenes by an aggregated atropisomeric platinum(II) complex

Supporting information

Annika Huber, Alessandro Prescimone, Christof Sparr* and Oliver S. Wenger*

[*] A. Huber, A. Prescimone, C. Sparr, O. S. Wenger

Department of Chemistry, University of Basel

St. Johanns-Ring 19, 4056 Basel (Switzerland)

E-mail: christof.sparr@unibas.ch, oliver.wenger@unibas.ch

Table of Contents

[1. General equipment and methods S2](#_Toc217728760)

[1.1 General procedure for UV-vis absorption and CD measurements of aggregated ***S*-1** S4](#_Toc217728761)

[1.2 General method for ***S*-1** recovery after analyte sensing S4](#_Toc217728762)

[2. DFT optimized structures S5](#_Toc217728763)

[3. Experimental procedures S7](#_Toc217728764)

[3.1 Preparation of the monodentate atropisomeric ligand S9](#_Toc217728765)

[3.2 Preparations of Pt(II) complexes S17](#_Toc217728766)

[4. Additional photophysical data S19](#_Toc217728767)

[5. Scanning electron microscope (SEM) images of ***S*-1** S24](#_Toc217728768)

[6. ^1^H NMR comparisons S26](#_Toc217728769)

[6.1 NMR titrations of aggregated ***S*-1** with *R*/*S* 2-BuOH S26](#_Toc217728770)

[6.2 NMR spectra before- and after sensing and recovery S28](#_Toc217728771)

[7. NMR and HRMS data S30](#_Toc217728772)

[7.1 All intermediates and the isocyanide ligand **8** S30](#_Toc217728773)

[7.2 Pt(II) complexes **9** and ***S*-1** S60](#_Toc217728774)

[8. FTIR spectra S73](#_Toc217728775)

[9. Chiral HPLC data S77](#_Toc217728776)

[10. X-Ray crystal structure of **10** S79](#_Toc217728777)

[11. References S82](#_Toc217728778)

## 1. General equipment and methods

All chemicals and solvents for synthesis or analysis were obtained from commercial suppliers in high purity and were used without further purification unless otherwise noted. 2‑Iodoxybenzoic acid (IBX) was prepared according to a literature procedure.^[1]^ *n‑*Dibutylmagnesium solution (*n-*Bu_2_Mg) in heptane was purchased from Acros, *n*‑butyllithium solution (*n-*BuLi) in hexanes from Acros, citrate buffer solution (pH 5) from J.T. Baker, DMF (peptide synthesis grade) from Acros and chloroform (EtOH-free) from Sigma Aldrich. Concentrations of *n-*Bu_2_Mg and *n-*BuLi were determined by titration with iodine.^[2]^

Analytical and preparative thin layer chromatography (TLC) was performed on pre-coated Merck silica gel 60 F254 plates (0.25 mm) and visualized by UV (254 nm or 365 nm). Concentration under reduced pressure was performed by rotary evaporation to ~10 mbar at 40 °C. Final products were dried at ~10^–2^ mbar on the Schlenk line at room temperature and stored in screw-cap glass vials under Ar at –19 °C.

Column chromatography of reaction products was performed on a Biotage chromatography instrument or manually on Silicycle SiliaFlash P60 (230 – 400 mesh).

NMR spectra were recorded either on a Bruker Avance III instrument operating at 400 MHz proton frequency or on a Bruker Avance III operating at 500 MHz proton frequency. Measurements were performed at 298 K in 5 mm standard NMR tubes using commercial deuterated solvents from Apollo Scientific. All 600 MHz spectra such as full assignment, dynamics or ^195^Pt NMR were obtained from a Bruker Avance III NMR spectrometer operating at 600.13 MHz proton frequency equipped with an indirect 5 mm BBI probe or from a Bruker Avance III HD NMR spectrometer operating at 600.13 MHz proton frequency equipped with a cryogenic 5 mm four-channel QCI probe (H/C/N/F). All chemical shifts are reported in δ values in ppm referred to residual non-perdeuterated solvent. All coupling constants *J* are stated in Hertz (Hz). The signals and their coupling patterns were described using the following abbreviations: s (singlet), d (doublet), dd (doublet of doublets), ddd (doublet of doublets of doublets), t (triplet), m (multiplet), b (broad).

Melting points (m.p.) were measured on a Büchi B-545 melting point apparatus and are uncorrected. Infrared (IR) spectra were measured on an ATR Varian Scimitar 800 FT-IR spectrometer and wavenumber ν was reported in cm^–1^.

Enantiomeric ratios were determined by HPLC on a chiral stationary phase using the indicated analytical columns at 1 mL/min in *i-*PrOH/heptane, 10/90, eluent. The retention times (R_t_) were reported in minutes.

Optical rotations were obtained at 24 °C on a Jasco P-2000 polarimeter at 589 nm using a 1.00 mL cell with a length of 100 mm and are reported in (°·mL)/(g·dm) with concentrations in g/100 mL.

All optical spectroscopic experiments were carried out at 293 K and solutions in quartz cuvettes (1 cm path length) with septum caps.

Steady-state absorption was recorded on a Cary 5000 spectrophotometer (Varian). Luminescence spectra were obtained from a Fluorolog-3-22 instrument (Horiba Jobin-Yvon) with a xenon arc lamp as excitation source. CD Measurements were performed on a JASCO J-1500 CD Spectrophotometer.

Electronspray ionization high resolution mass spectra (ESI-HRMS) were recorded by Dr. Michael Pfeffer (University of Basel, Department of Chemistry) on a Bruker maxis 4G ESI-Q-TOF instrument.

Elemental Analysis was performed by Ms. Sylvie Mittelheisser (University of Basel, Department of Chemistry), with a Vario Micro Cube instrument from Elementar. The measured elemental composition did not match the calculated values. One probable reason could be incomplete combustion due to the two CF_3_-groups on the ligand.

X-Ray crystal structures were measured by Dr. Alessandro Prescimone at 150 K on a Bruker Kappa Apex2 diffractometer using graphite monochromated Cu Kα-radiation with λ = 1.54178 Å, Θ_max_ = 68.976°.

### 1.1 General procedure for UV-vis absorption and CD measurements of aggregated ***S*-1**

Quartz glass cuvettes were cleaned with aqua regia, distilled water, and acetone before use. A stock solution of complex ***S*-1** (1.03 mg, 0.969 mmol) was prepared in dry CHCl_3_ (3.00 mL). The stock solution was ultrasonicated for 15 s. The colloidal dispersions of ***S*-1** were formed by diluting 300 μL of the stock solution with 2.70 mL dry *n*‑hexane. Visible particles appeared within 10-20 s. The mixture was stirred before being transferred into a quartz glass cuvette. The cuvette was closed by a screw cap with a septum and the dispersion was manually shaken directly before starting the UV-vis measurement. Stock solutions of selected enantiomers were prepared by adding pure compound (50 μL) to dry CHCl_3_/ *n*-hexane, 1/9 (3.00 mL). Stepwise dilution was achieved by diluting 10 μL of the initial enantiomer solution in dry CHCl_3_/ *n*-hexane, 1/9 (2.99 mL). Each solution was mixed before further manipulation. When titrating the colloidal dispersions of ***S*-1** with enantiomer dilutions, 10 μL of a dilution were added to the cuvette and the sample was shaken for 10 s directly before starting the measurement.

For each measurement or titration series, 0.3 mL of the ***S*-1** stock solution (3.00 mL) were required. One stock solution could be used for ten different samples, which increased consistency.

### 1.2 General method for ***S*-1** recovery after analyte sensing

After completed measurements complex ***S*-1** was recycled for further investigations. The mixtures were collected in a flask and concentrated under reduced pressure. The residual yellow solid was dissolved in a minimal amount of CHCl_3_, suspended in *n*-hexane, and precipitated by centrifugation in glass tubes (5`000 rpm, 3 min). The solvent was decanted, the precipitate was suspended with *n*‑hexane, and the mixture was centrifuged (5`000 rpm, 3 min). This procedure was repeated three times before collecting the yellow solid and drying *in vacuo*. ^1^H NMR confirmed the recovery of complex ***S*-1** and removal of previously used VOCs (section 6.2, S28).

## 2. DFT optimized structures

All density functional theory (DFT) calculations were carried out for the gas phase using the ORCA 4.1.2 package.^[3–5]^ Geometry optimizations were performed with the composite approach PBEh- 3c,^[6]^ the basis def2-mSVP(C,H,N,O)^[6]^ with the atom pairwise dispersion correction with the Becke−Johnson damping scheme (D3BJ),^[7,8]^ the RIJCOSX approximation, the auxiliary basis def2/J,^[9]^ fine numerical integration grids (grid4, gridX4, and NoFinalGrid in ORCA 4 nomenclature), and KDIIS+SOSCF. The crystallographic structures of similar reported compounds (MIGFOA, UZARIY, and ZOMLAQ) were used as a starting point.^[10–12]^ The libint2 library was used for the computation of 2-el integrals.^[13]^ The output results were handled with the ChemCraft software package.^[14]^


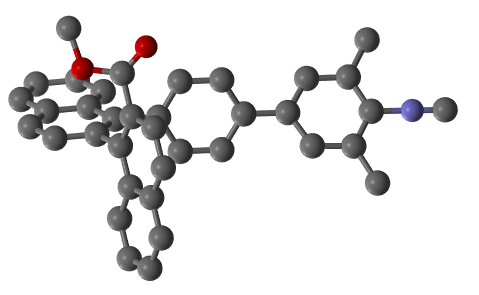


**Figure S1.** DFT-optimized structure of the isocyanide ligand with the atropisomeric binaphthyl-moiety in the periphery. Hydrogen atoms were omitted for clarity.


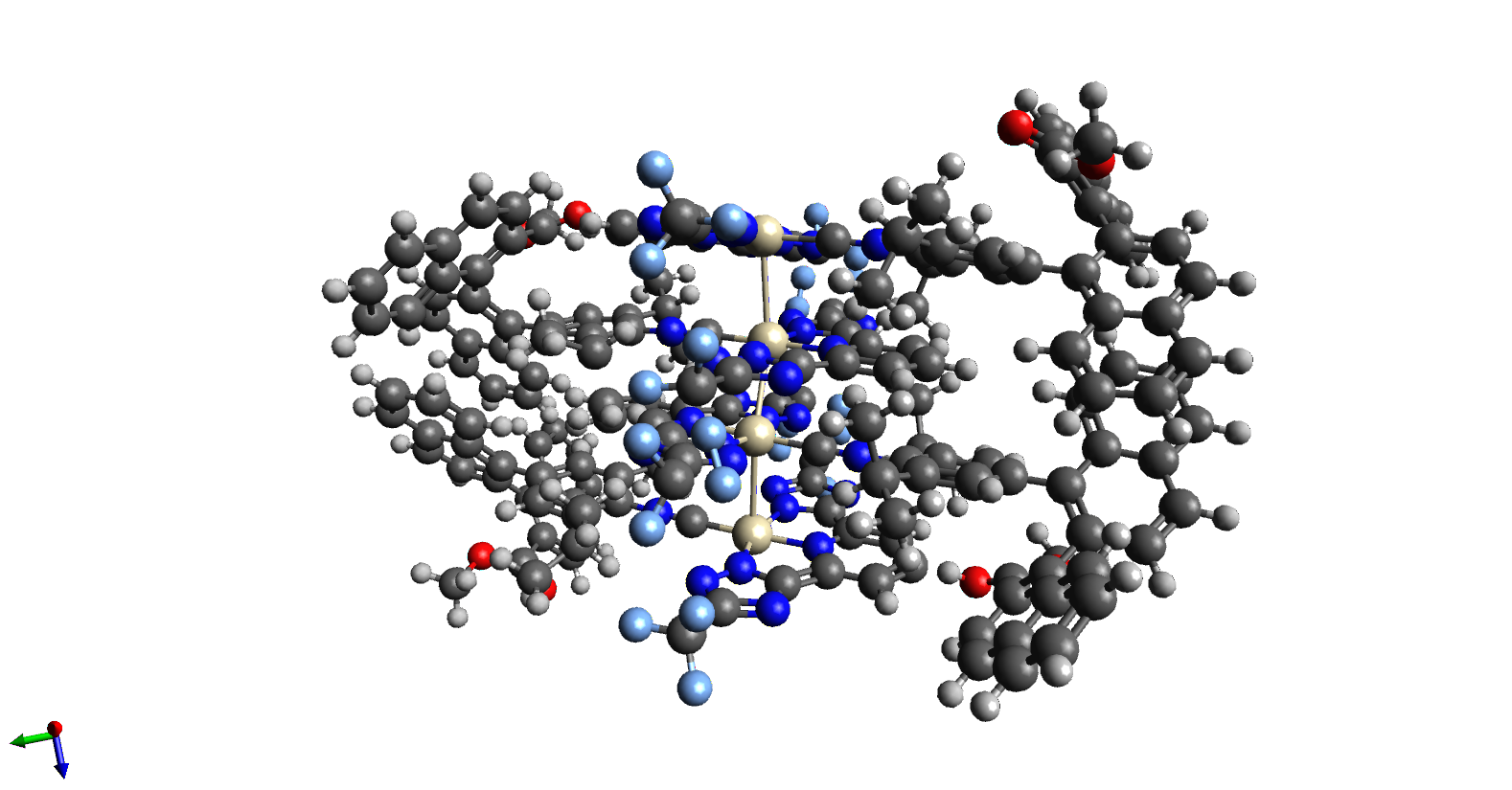


**Figure S2.** Side-view of the simulated head-to-tail aggregation of four **S-1** complexes. The light grey bonds between the Pt(II) centers (gold) simulate possible Pt-Pt interactions due to distances <3.5 Å.


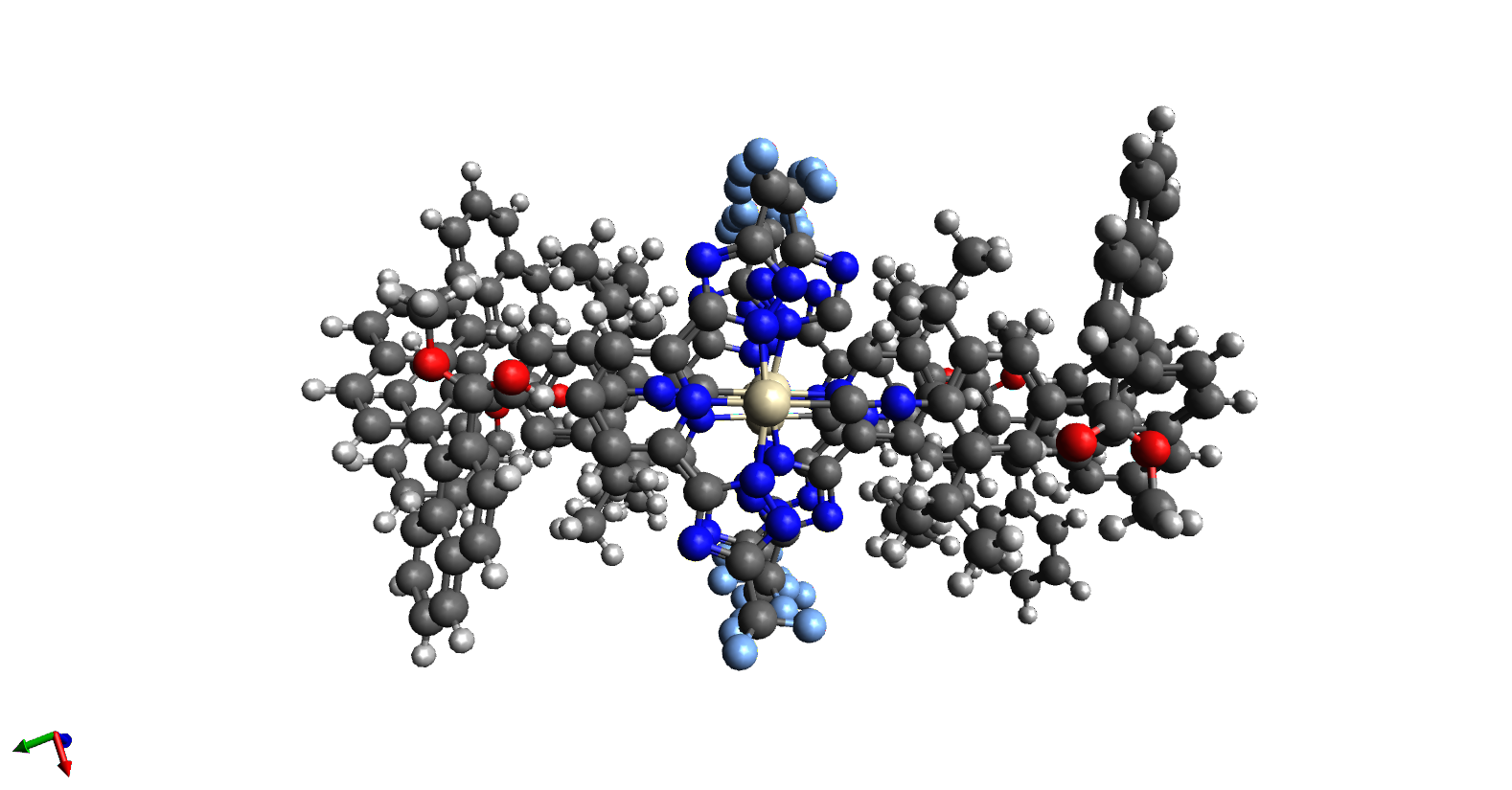


**Figure S3.** Top-view of the simulated head-to-tail aggregation of four **S-1** complexes. A slight helical displacement between individual complexes is visible.

## 3. Experimental procedures

No unexpected or unusually high safety hazards were encountered. Isocyanides as well as platinum(II) [Pt(II)] complexes are potentially toxic or cancerogenic and should be handled with care.

The following compounds were prepared in good yields, following reported procedures: *N*-formyl-4-bromo-2,6-dimethylaniline,^[15]^ (Scheme S1a) 4-isocyano-3,5-diisopropyl-3’,5’-dimethoxy-1,1’-biphenyl (monodent-RNC),^[16]^ (Scheme S1b), iodoxybenzoic acid,^[1]^ 4-(2-bromophenyl)but-3-yn-1-ol,^[17]^ *Z*-4-(2-bromophenyl)but-3-en-1-ol. ^[17]^ (Scheme S1c).

**Scheme S1**. Synthesis of reported compounds that were used as reagents and for test reactions towards reference compounds.

The ligand trzpy^[18]^ as well as the corresponding parent complex Na[Pt(trzpy)Cl]^[18]^ was prepared according to slightly modified literature procedures (Scheme S2).

**Scheme S2.** Synthesis of the reported trzpy ligand and the parent Pt(II) complex Na[Pt(trzpy)Cl] applying slightly modified literature procedures^[18]^.

### 3.1 Preparation of the monodentate atropisomeric ligand

#### 3.1.1 (Z)-4-(2-((4-Bromophenyl)(hydroxy)methyl)phenyl)but-3-en-1-ol (**2**)

####

The starting materials each were dissolved in toluene (15 mL) and concentrated under reduced pressure. The procedure was repeated twice.

(*Z*)-4-(2-Bromophenyl)but-3-en-1-ol (**1**, 1.87 g, 7.00 mmol, 1.40 eq.) in dry THF (100 mL) was cooled to 0 °C and *n*-Bu_2_Mg (in heptane, 0.68 M, 0.78 eq., 5.74 mL) was added over 5 min. The light yellow solution was stirred at 0 °C for 45 min before *n*-BuLi (in hexanes, 0.80 M, 9.75 mL, 1.56 eq.) was added over 15 min. The yellow mixture was stirred for 1 h at 0 °C, before it was added over 45 min to a solution of a 4–bromobenzaldehyde (925 mg, 5.00 mmol, 1.00 eq.) in dry THF (50 mL/mmol, 100 mL). The mixture was stirred at rt (27 °C). ^1^H-NMR showed full conversion. After 2 h, aq. sat. NH_4_Cl (10 mL/mmol) and EtOAc (10 mL/mmol) were added, the layers were separated and the aqueous layer was extracted with EtOAc (2 × 30 mL/mmol). The combined organic layers were washed with brine (20 mL), dried over Na_2_SO_4_, filtered, and concentrated under reduced pressure. The crude products were purified by column chromatography (50 g silica, 0 – 70% EtOAc in cyclohexane) to give a white amorphous solid (**2**, 1.23 g, 3.69 mmol, 74%).

m.p.: 129.2 °C; R*_f_* 0.50 (Cy/EtOAc, 1/1); ν_max_ (neat) = 3327bm, 2924w, 2882w, 1485m, 1400m, 1028m, 908m, 733s, 649w; ^1^H NMR hua.844.1 (400 MHz, CDCl_3_) δ [ppm] = 7.42 – 7.37 (m, 2H, C13*H*, C13`*H*), 7.32 – 7.27 (m, 1H, C8*H*), 7.24 – 7.21 (m, 2H, C14*H*, C14`*H*), 7.20 – 7.17 (m, 2H, C6*H*, C7*H*), 7.14 – 7.10 (m, 1H, C9*H*), 6.61 (dd, ^3^*J* = 11.3 Hz, ^4^*J* = 1.9 Hz, 1H, C4*H*), 5.93 (s, 1H,C11*H*), 5.74 (ddd, ^3^*J* = 11.3 Hz, 8.3 Hz, 6.5 Hz, 1H, C3*H*), 3.57 (ddd, ^3^*J* = 5.5 Hz, ^4^*J* = 2.9 Hz, 2H, C1*H*), 2.38 – 2.16 (m, 2H, C2*H*), 2.14 (s, 2H, O*H*); ^13^C NMR hua.844.2 (126 MHz, CDCl_3_) δ [ppm] = 142.4 (*C*10), 141.59 (*C*12), 134.0 (*C*5), 131.3 (*C*13, *C*13`), 130.7 (*C*4), 130.3 (*C*3), 129.9 (*C*9), 128.6 (*C*14, *C*14’), 127.6 (*C*8), 127.5 (*C*7), 127.1 (*C*6), 121.1 (*C*15), 72.2 (*C*11), 61.8 (*C*1), 31.5 (*C*2); ESI-MS: m/z calcd. for C_17_H_17_BrNaO_2_^+^ 355.0304 found 355.0308 [M+Na^+^].

#### 3.1.2 1-(4-Bromophenyl)-2-naphthaldehyde (**3**)

####

IBX (7.22 g, 25.8 mmol, 3.00 eq.) was added to a light yellow solution of diol-precursor (2.87 g, 8.60 mmol, 1.00 eq.) in MeCN (410 mL, 21 mmol/L), the white suspension was sonicated for 30 s and vigorously stirred at 50 °C for 16 h. ^1^H NMR showed full conversion. The mixture was cooled to rt (30 °C), filtered and concentrated under reduced pressure. CHCl_3_ (430 mL, 20 mmol/L) was added to the residue, the mixture was filtered and directly used in the next step. To the keto-aldehyde solution, DMF peptide grade (270 mL, 30 mmol/L), (*S*)-(–)-5-(2-pyrrolidinyl)-*1H*-tetrazole (430 mg, 3.10 mmol, 36.0 mol%), and citrate buffer (pH 5, 280 mL, 30 mmol/L) was added. The yellow biphasic mixture was shortly sonicated and then vigorously stirred at rt for 6 h. The conversion was monitored by mini work-up and ^1^H NMR. The layers were separated and the aq. layer was extracted with CH_2_Cl_2_ (2 × 50 mL). The combined org. layers were concentrated under reduced pressure (to 200 mbar), redissolved in EtOAc/Cy (1/1, 400 mL), washed with H_2_O (5 × 80 mL) and brine (50 mL). The org. layer was dried over Na_2_SO_4_, filtered and the solvent was removed under reduced pressure. The crude aldol condensation product was purified by column chromatography (100 g silica, 0 – 30% EtOAc in cyclohexane) to give a light yellow solid (**3**, 2.03 g, 6.52 mmol, 76% over two steps)

m.p.: 133.9 °C; R*_f_* 0.72 (Cy:EtOAc, 4:1); ν_max_ (neat) = 3063w, 2890w, 2363w, 1689s, 1490w, 1242w, 907s, 729s, 632m; ^1^H NMR hua.871.1 (400 MHz, CDCl_3_) δ [ppm] = 9.90 (d, ^4^*J* = 0.87 Hz, 1H, C1*H*), 8.06 (d, ^3^*J* = 8.6 Hz, 1H), 7.98 – 7.92 (m, 2H), 7.71 – 7.67 (m, 2H), 7.66 – 7.58 (m, 2H, C13*H*, C13`*H*), 7.48 (ddd, ^3^*J* = 8.4 Hz, 6.7 Hz, ^4^*J* = 1.3 Hz, 1H), 7.32 – 7.28 (m, 2H, C14*H*, C14`*H*); ^13^C NMR hua.873.2 (126 MHz, CDCl_3_) δ [ppm] = 192.3 (*C*1), 145.1 (*C*_q_), 136.2(*C*_q_), 134.3(*C*_q_), 132.7 (*C*13, *C*13`), 132.3(*C*_q_), 131.7 (*C*14, *C*14`), 131.3(*C*_q_), 129.1, 128.9, 128.5, 127.5, 127.3, 122.9 (*C*15), 122.4; ESI-MS: m/z calcd. for C_17_H_11_BrNaO^+^ 332.9885 found 332.9884 [M+Na^+^].

#### 3.1.3 (Z)-4-(2-((1-(4-Bromophenyl)naphthalen-2-yl)(hydroxy)methyl)phenyl)but-3-en-1-ol (**4**)

####

**1** was dissolved in toluene (15 mL) and concentrated under reduced pressure. The procedure was repeated twice. **3** was dried the same way.

**1** (243 mg, 0.91 mmol, 1.50 eq.) in dry THF (12 mL, 60 mL/mmol) was cooled to 0 °C and *n*-Bu_2_Mg (in heptane, 0.92 M, 0.55 mL, 0.78 eq.) was added over 7 min. The light yellow solution was stirred at 0 °C for 45 min before *n*-BuLi (in hexanes, 0.82 M, 1.24 mL, 1.56 eq.) was added over 10 min. The slightly turbid yellow mixture was stirred for 2 h at 0 – 4 °C, before being divided into three parts and added each over 7 min to three parts of a solution of aldehyde **3** (202 mg, 0.650 mmol, 1.00 eq.) in dry THF (12 mL, 60 mL/mmol). The mixtures were stirred at rt for 16 h. Aq. sat. NH_4_Cl (10 mL/mmol), water (5 mL/mmol), and EtOAc (50 mL/mmol) were added. The layers were separated, and the aqueous layer was extracted with EtOAc (2 × 30 mL/mmol). The combined organic layers were washed with brine (20 mL), dried over Na_2_SO_4_, filtered, and concentrated under reduced pressure. The crude products were purified by column chromatography (25 g silica, 0 – 70% EtOAc in cyclohexane) to give a yellow oil (**4**, 194 mg, 0.420 mmol, 65%).

m.p.: 110.0 – 112.4 °C; R*f* 0.20 (Cy:EtOAc, 6:1); ν_max_ (neat) = 3322bm, 2930w, 2362w, 1488m, 1390m, 1042s, 908s, 820w, 730s; ^1^H NMR hua.871.1 (400 MHz, CDCl_3_) δ [ppm] = 7.93 (d, ^3^*J* = 8.6 Hz, 1H, C19*H*), 7.90 – 7.87 (m, 1H, C16*H*), 7.83 (d, ^3^*J* = 8.7 Hz, 1H, C14*H*), 7.63 (dd, ^3^*J* = 8.1 Hz, ^4^*J* = 2.2 Hz, 1H, C18*H*), 7.46 (ddd, ^3^*J* = 8.1 Hz, 6.6 Hz, ^4^*J* = 1.5 Hz, 1H, C17*H*), 7.39 – 7.36 (m, 1H, C24*H*), 7.35 – 7.33 (m, 1H, C24`*H*), 7.32 – 7.28 (m, 1H, C23*H*), 7.27 – 7.24 (m, 1H, C23`*H*), 7.20 – 7.17 (m, 3H, C6*H*, C7*H*, C8*H*), 7.03 – 7.01 (m, 1H, C9*H*), 6.59 (dd, ^3^*J* = 8.1 Hz, ^4^*J* = 2.2 Hz, 1H, C13*H*), 6.00 (d, ^3^*J* = 11.3 Hz, 1H, C4*H*), 5.85 (s, 1H, C11*H*), 5.57 (ddd, ^3^*J* = 11.4 Hz, 9.1 Hz, 5.9 Hz, 1H, C3*H*), 3.50 (m, 2H, C1*H*), 2.30 – 2.00 (m, 2H, C2*H*) 1.60 (bs, 2H, O*H*); ^13^C NMR hua.873.2 (126 MHz, CDCl_3_) δ [ppm] = 142.0, 138.0, 137.4, 136.5, 136.4, 133.0, 132.7, 132.5, 132.0, 131.7, 131.3, 130.5, 129.6, 128.2, 128.1, 127.5, 127.4, 127.4, 126.4, 126.3, 126.0, 124.6, 121.7, 70.5 (*C*11), 62.0 (*C*1), 31.4 (*C*2).; ESI-MS: m/z calcd. for C_27_H_23_BrNaO2^+^ 481.0774 found 481.0770 [M+Na^+^].

#### 3.1.4 (S)-1'-(4-Bromophenyl)-[1,2'-binaphthalene]-2-carbaldehyde (**S-5**)

####

IBX (353 mg, 1.26 mmol, 3.00 eq.) was added to a light yellow solution of **4** (193 mg, 0.42 mmol, 1.00 eq.) in MeCN (21 mL, 20 mmol/L), the white suspension was sonicated for 30 s and vigorously stirred at 50 °C for 26 h. The mixture was cooled to rt, filtered and concentrated under reduced pressure. CHCl_3_ (21 mL, 20 mmol/L) was added to the residue and the mixture was filtered again to yield a yellow solution, which was directly used in the next step. To the keto-aldehyde, DMF peptide grade (4 mL), (*S*)-(–)-5-(2-pyrrolidinyl)-1*H*-tetrazole (23.4 mg, 0.17 mmol, 41.0 mol%) and citrate buffer (pH5, 13 mL, 30 mmol/L) was added. The yellow biphasic mixture was sonicated for 30 s and vigorously stirred at rt for 5 h. The conversion was monitored by mini work-up and ^1^H NMR. The layers were separated and the aq. layer was extracted with CH_2_Cl_2_ (3 × 15 mL). The combined org. layers were washed with H_2_O (4 × 10 mL and brine (10 mL). The org. layer was dried over Na_2_SO_4_, filtered and the solvent was removed under reduced pressure. The crude product was purified by column chromatography (10 g silica, 0 – 70% EtOAc in cyclohexane), yielding a yellow solid (***S*-*5***, 67.6 mg, 0.42 mmol, 37%).

R*_f_* 0.65 (Cy:EtOAc, 4:1); ν_max_ (neat) = 3059m, 2926m, 2852m, 2362w, 1679s, 1238m, 1012m, 821s, 750s; ^1^H NMR hua.1048.1 (400 MHz, CDCl_3_) δ [ppm] = 9.90 (d, ^4^*J* = 0.9 Hz, 1H, C1*H*), 8.05 – 8.01 (m, 2H, C16*H*, C14*H*), 7.88 – 7.84 (m, 2H, C3*H*, C4*H*), 7.82- 7.79 (m, 1H, C9*H*), 7.62 – 7.56 (m, 4H, C6*H*, C7*H*, C17*H*, C19*H*), 7.52 (d, ^3^*J* = 8.4 Hz, 1H, C13*H*), 7.50 (ddd, ^3^*J* = 8.0 Hz, 7.4 Hz, ^4^*J* = 1.3 Hz ,1H, C8H), 7.43 (ddd, ^3^*J* = 8.4 Hz, 6.9 Hz, ^4^*J* = 1.4 Hz, 1H, C18*H*), 7.34 (dd, ^3^*J* = 8.2 Hz, ^4^*J* = 2.2 Hz, 1H, C23*H*), 7.03 (ddd, ^3^*J* = 8.2 Hz, 5.1 Hz, ^4^*J* =2.2 Hz, 2H, C23`*H*, C24`*H*), 6.69 (dd, ^3^*J* = 8.2 Hz, ^4^*J* =2.2 Hz, 1H, C24*H*); ^13^C NMR hua.878.2 (126 MHz, CDCl_3_) δ [ppm] = 192.3 (*C*1), 145.8 (*C*2), 139.2 (*C*21), 137.2 (*C*22), 136.0 (*C*12), 133.6 (*C*15), 132.9 (*C*10/*C*20), 132.4 (*C*5), 132.0 (*C*10/20), 131.8 (*C*24/ *C*24`), 131.2 (*C*24/ *C*24`), 131.2 (*C*23), 131.1 (*C*23`), 130.9 (*C*11), 128.9 (*C*19), 128.6 (*C*4), 128.5(*C*9, *C*6), 128.4 (*C*13), 128.0 (*C*16/*C*14), 127.8(*C*16/*C*14), 127.3 (*C*17), 127.0 (*C*7/*C*8), 126.8 (*C*18), 126.6 (*C*7/*C*8), 122.1 (*C*3), 121.6 (*C*25); ESI-MS: m/z calcd. for C_27_H_17_BrNaO^+^ 459.0355 found 459.0350 [M+Na^+^].

#### 3.1.5 (S)-1'-(4-Bromophenyl)-[1,2'-binaphthalene]-2-carboxylic acid (**10**)

####

***S*-5** (48.1 mg, 0.11 mmol, 1.00 eq) was dissolved in THF (3.0 mL, 35 mmol/L) and *t*-BuOH (3.0 mL, 35 mmol/L) and 2-methyl-2-butene (0.15 mL, 1.32 mmol, 12.0 eq.) was added. To the solution, a solution of sodium chlorite (30.9 mg, 0.33 mmol, 3.05 eq.) and NaH_2_PO_4_ (92.1 mg, 0.66 mmol, 6.00 eq.) in H_2_O (5.0 mL, 20 mmol/L) was added at 0 °C, followed by aq. H_2_O_2_ (35%, 0.02 mL, 2.0 eq.). Gas formation was observed. The light yellow mixture was stirred at rt for 16 h. TLC (cyclohexane, EtOAc, 4/1) showed full conversion. Aq. HCl (1.0 M, 7 mL) was added and the mixture was extracted with EtOAc (3 × 20 mL). The combined org. layers were washed with brine (15 mL), dried over Na_2_SO_4_, filtered, and concentrated under reduced pressure to give a light yellow resin (**10**, 49.9 mg, 0.11 mmol, quant.).

R*_f_* 0.26 (Cy:EtOAc, 4:1); ^1^H NMR hua.902.1 (400 MHz, CDCl_3_) δ [ppm] = 7.99 (d, ^3^*J* = 8.0 Hz, 1H), 7.96 (d, ^3^*J* = 8.4 Hz, 1H), 7.90 (d, ^3^*J* = 8.7 Hz, 1H), 7.81 (d, ^3^*J* = 8.2 Hz, 1H), 7.77 (d, ^3^*J* = 8.7 Hz, 1H), 7.58 – 7.52 (m, 1H), 7.52 – 7.48 (m, 2H), 7.47 – 7.43 (m, 2H), 7.41 (d, ^3^*J* = 8.4 Hz, 1H), 7.35 (ddd, ^3^*J* = 8.5 Hz, 6.8 Hz, ^4^*J* = 1.3 Hz, 1H), 7.18 (dd, ^3^*J* = 8.2 Hz, ^4^*J* =2.1 Hz, 1H), 7.08 (dd, ^3^*J* = 8.2 Hz, ^4^*J* = 2.2 Hz, 1H), 7.01 (dd, ^3^*J* = 8.2 Hz, ^4^*J* = 2.2 Hz, 1H), 6.70 (dd, ^3^*J* = 8.2 Hz, ^4^*J* = 2.2 Hz, 1H); ^13^C NMR hua.902.2 (101 MHz, CDCl_3_) δ [ppm] = 171.1 (*C*1), 137.6, 136.9, 135.5, 135.2, 133.3, 132.9, 132.7, 132.0, 131.2, 130.9, 130.6, 128.4, 128.3, 128.1, 128.0, 127.9, 127.6, 126.7, 126.6, 126.5, 126.3, 126.1, 126.0, 121.2 (*C*25); ESI-MS: m/z calcd. for C_27_H_16_BrO_2_^-^ 451.0339 found 451.0348 [M^-^].

#### 3.1.6 Methyl (S)-1'-(4-bromophenyl)-[1,2'-binaphthalene]-2-carboxylate (**S-6**)

####

**10** (204 mg, 0.45 mmol, 1.00 eq.) was dissolved in dry MeOH (9.00 mL, 0.5 mol/L) and H_2_SO_4_ (0.24 mL, 4.50 mmol, 10.0 eq.) was added and the solution was heated to reflux (70 °C) for 16 h. TLC (cyclohexane/EtOAc, 4/1) showed full conversion. After cooling to rt, the solution was diluted with Et_2_O (20 mL) and aq. sat. NaHCO_3_ (15 mL). The layers were separated, and the aq. layer was extracted with Et_2_O (2 × 20 mL). The combined org. layers were dried over Na_2_SO_4_, filtered and evaporated. The residue was purified by column chromatography (12 g silica, 0 – 40% EtOAc in cyclohexane) to obtain the product as an off-white solid (***S*-6**, 179 mg, 0.38 mmol, 85%). Crystals suitable for X-ray crystallography were grown from CH_2_Cl_2_/EtOH.

R*_f_* 0.80 (Cy/EtOAc, 4/1); ν_max_ (neat) = 3057m, 2949w, 2360w, 1725m, 1243s, 1135m, 822m, 748s; ^1^H NMR hua.911.1 (400 MHz, CDCl_3_) δ [ppm] = 7.99 (d, ^3^*J* = 8.4 Hz, 2H, C15*H*, C17*H*), 7.85 (d, ^3^*J* = 8.7 Hz, 1H, C4*H*), 7.81 (d, ^3^*J* = 8.3 Hz, 1H, C7*H*), 7.74 (d, ^3^*J* = 8.8 Hz, 1H, C5*H*), 7.57 – 7.49 (m, 3H, C8*H*, C18*H*, C20*H*), 7.46 (d, ^3^*J* = 8.6 Hz, 1H, C10*H*), 7.42 (d, ^3^*J* = 8.4 Hz, 1H, C14*H*), 7.45 – 7.38 (m, 1H, C19*H*), 7.35 (ddd, ^3^*J* = 8.4 Hz, 6.7 Hz, ^4^*J* = 1.3 Hz, 1H, C9*H*), 7.28 (dd, ^3^*J* = 8.2 Hz, ^4^*J* = 2.0 Hz, 1H, C25`*H*), 7.09 (dd, ^3^*J* = 8.2 Hz, ^4^*J* = 2.0 Hz, 1H, C24`*H*), 7.03 (dd, ^3^*J* = 8.2 Hz, ^4^*J* = 2.0 Hz, 1H, C25*H*), 6.74 (dd, ^3^*J* = 8.2 Hz, ^4^*J* = 2.0 Hz, 1H, C24*H*), 3.65 (s, 3H, C1*H*); ^13^C NMR hua.919.2 (126 MHz, CDCl_3_) δ [ppm] = 167.8 (*C*1), 141.9 (*C*12), 137.7 (*C*23), 135.8 (*C*26), 135.0 (*C*22), 134.8 (*C*6), 133.2 (*C*13), 132.8 (*C*16),132.7 (*C*21), 132.0 (*C*11), 131.3 (*C*24`), 130.7, 130.6 (*C*24), 128.4 (*C*10), 128.2 (*C*14), 128.08 (*C*7), 128.05 (*C*3), 127.8 (*C*5), 127.7 (*C*8), 127.6 (*C*15), 127.5 (*C*20), 127.0 (*C*25), 126.6 (*C*9, *C*19), 126.5 (*C*17, *C*25`), 126.0 (*C*18), 125.7 (*C*4), 52.1 (*C*1); ESI-MS: m/z calcd. for C_28_H_19_BrNaO_2_^+^ 489.0468 found 489.0461 [M+Na^+^].

#### 3.1.7 1-N-Formamide-2,6-Me_2_-Ph-4-Ph-Naphth_2_-CO_2_Me (**11**)

A white suspension of ***S*-6** (60.3 mg, 0.130 mmol, 1.00 eq.), boronic acid bis(pinacolone)ester **7** (44.8 mg, 0.163 mmol, 1.20 eq.), and K_2_CO_3_ (45.8 mg, 0.325 mmol, 2.5 eq.) in THF (4.0 mL) and H_2_O (2.0 ml) was degassed by bubbling Ar for 10 min. Pd(PPh_3_)_4_ (11.0 mg, 1.3 10^-3^ mmol, 0.10 eq.) was added and the mixture was degassed for 15 min. The yellow suspension was heated to 90 °C for 18 h. TLC (EtOAc, Cy, 1/2) showed full conversion. The reaction mixture was cooled to rt (32 °C), the organic layer was concentrated onto a portion of celite and purified by column chromatography (12 g, silica, 0 – 70% EtOAc in Cy) to give an off-white amorphous solid (**11**, 66.3 mg, 0.124 mmol, 95%). In ^1^H NMR, a mixture of *E* and *Z* isomers of the formamide was observed. The compound was used as is for the next step.

R*_f_* 0.20 (Cy/EtOAc, 2/1); ν_max_ (neat) = 3237bw, 2980w, 2362m, 1676s, 1506m, 1383s, 1243s, 1140s, 839m, 729s, 647s; ^1^H NMR hua.1097.1 (400 MHz, CDCl_3_) δ [ppm] = 8.43 – 8.41 (m, 1H, C32*H*), 8.08 (d, ^3^*J* = 8.2 Hz, 2H, C15*H*, C17*H*), 7.84 (dd, ^3^*J* = 8.7 Hz, ^4^*J* = 2.6 Hz, 1H, C7*H*), 7.81 – 7.77 (m, 1H, C4*H*), 7.74 (d, ^3^*J* = 8.7 Hz, 1H, C5*H*), 7.71 – 7.64 (m, 2H), 7.62 (d, ^3^*J* = 8.9 Hz, 1H), 7.58 – 7.51 (m, 2H), 7.50 – 7.42 (m, 2H), 7.45 (d, ^3^*J* = 8.4 Hz, 1H, C14*H*), 7.39 – 7.29 (m, 2H), 7.22 (s, 1H, C28*H*), 7.19 (s, 1H, C28*H*), 7.14 – 7.07 (m, 1H, C25*H*), 6.93 – 6.86 (m, 1H, C24*H*), 3.65 (s, 3H, C1*H*), 2.30 (s, 3H, C30*H*), 2.27 (s, 3H, C30*H*); ESI-MS: m/z calcd. for C_37_H_29_NNaO3^+^ 558.2040 found 558.2034 [M+Na^+^].

#### 3.1.8 1-NC-2,6-Me_2_-Ph-4-Ph-Naphth_2_-CO_2_Me (**S-8**)

####

Dry diisopropylamine (0.28 ml from a dilution of 0.10 mL amine in 1.00 mL CH_2_Cl_2_, 0.197 mmol, 3.50 eq) was added to a solution of **11** (29.5 mg, 0.055 mmol, 1.00 eq.) in dry CH_2_Cl_2_ (1.00 ml). After cooling to 0 °C, POCl_3_ (0.10 mL from a dilution of 0.10 mL in 1.00 mL CH_2_Cl_2_, 0.099 mmol, 1.80 eq.) was added dropwise. The yellow solution was stirred at 25 °C for 30 min, then a small sample was checked by TLC (CH_2_Cl_2_/EtOAc, 4/1), already showing full conversion. Diisopropylamine (0.500 ml from a dilution of 0.300 mL amine in 1.00 mL CH_2_Cl_2_) was added to equilibrate at pH8. The reaction mixture was diluted with CH_2_Cl_2_ (5 ml), a small portion of Celite was added and the mixture was concentrated under reduced pressure. Column chromatography (12 g silica, 0 – 90% EtOAc in cyclohexane) yielded a white solid (***S***-**8**, 19.0 mg, 0.037 mmol, 67%).

[α]_D_ = –122.5 °; m.p.: 100 °C; R*_f_* 0.74 (Cy/EtOAc, 9/1); ν_max_ (neat) = 3068w, 2930w, 2116m, 1723s, 1243s, 909s, 730s, 649s; ^1^H NMR hua.1153.1 (400 MHz, CDCl_3_) δ [ppm] = 8.00 (d, ^3^*J* = 8.4 Hz, 2H, C15*H*, C17*H*), 7.84 (d, ^3^*J* = 8.7 Hz, 1H, C4*H*), 7.79 (d, ^3^*J* = 8.3 Hz, 1H, C7*H*), 7.74 (d, ^3^*J* = 8.7 Hz, 1H, C5*H*), 7.60 (d, ^3^*J* = 8.5 Hz, 1H, C20*H*), 7.57 – 7.52 (m, 2H, C18*H*, C10*H* ), 7.50 (ddd, ^3^*J* = 8.1 Hz, 6.8 Hz, ^4^*J* = 1.2 Hz, 1H, C8*H*), 7.45 (d, ^3^*J* = 8.4 Hz, 1H, C14*H*), 7.44 – 7.42 (m, 1H, C19*H*), 7.41 – 7.34 (m, 2H, C25`*H*, C9*H*), 7.27 (dd, ^3^*J* = 8.0 Hz, ^4^*J* = 1.8 Hz, 1H, C24`*H*), 7.19 (s, 2H, C28*H*), 7.10 (dd, ^3^*J* = 7.9 Hz, ^4^*J* = 2.0 Hz, 1H, C25*H*), 6.92 (dd, ^3^*J* = 8.0 Hz, ^4^*J* = 1.8 Hz, 1H, C24*H*), 3.66 (s, 3H, C1*H*), 2.42 (s, 3H, C30*H*); ^13^C NMR hua.1075.2 (126 MHz, CDCl_3_) δ [ppm] = 167.9 (*C*1), 142.1 (*C*27), 141.1 (*C*12), 138.6 (*C*23), 137.9 (*C*29), 137.5 (*C*22), 135.8 (*C*26), 135.2 (*C*13), 134.8 (*C*6), 134.2 (*C*16), 133.3 (*C*11), 133.0 (*C*21), 132.9 (*C*24`), 130.9 (*C*28), 130.2 (*C*24), 128.4 (*C*10), 128.3 (*C*14), 128.2 (*C*7), 128.0 (*C*3), 127.7 (*C*5), 127.67 (*C*8), 127.6 (*C*15), 127.3 (*C*20), 126.7 (*C*9), 126.5 (*C*19), 126.4 (*C*17, *C*25`), 126.1 (*C*25), 126.0 (*C*18), 125.9 (*C*4), 125.7 (*C*31), 52.1 (*C*1), 19.1 (*C*30); ESI-MS: m/z calcd. for C_37_H_27_NNaO_2_^+^ 540.1930 found 540.1934 [M+Na^+^].

### 3.2 Preparations of Pt(II) complexes

#### 3.2.1 **Complex S-1**

Na[Pt(trzpy)Cl] (31.1 mg, 0.051 mmol, 1.6 eq.) was combined with ***S*-8** (16.9 mg, 0.032 mmol, 1.0 eq.) and AgPF_6_ (12.1 mg, 0.048 mmol, 1.5 eq.) in methanol (3.1 mL) under Ar atmosphere. After stirring at 50 °C for 2 h, the reaction mixture was filtered and the precipitate was washed with MeOH, CH_2_Cl_2_, and Et_2_O. The light yellow-greenish solid was washed with *n*-pentane. The compound was purified by size exclusion chromatography (2 cm diameter, 30 cm length, CH_2_Cl_2_). The combined fractions were extracted with MeCN/*n*-pentane, 1/2, the MeCN layer was concentrated under reduced pressure and washed with *n*-pentane. The residue was dried *in vacuo* to give a light yellow solid (***S-*1**,12.93 mg, 0.012 mmol, 38%).

m.p.: decomp. <240 °C; ν_max_ (neat) = 3064w, 2920w, 2851w, 2215m, 1730m, 1479m, 1161s, 991m, 822m, 655m; ^1^H NMR hua-444_c (600 MHz, CDCl_3_) δ [ppm] = 8.10 (dd, ^3^*J* = 7.9 Hz, 8.0 Hz, 1H, C38*H*), 8.01 (d, ^3^*J* = 8.3 Hz, 2H, C15*H*, C17*H*), 7.89 (d, ^3^*J* = 7.9 Hz, 2H, C37*H*), 7.87 (d, ^3^*J* = 8.7 Hz, 1H, C4*H*), 7.81 (d, ^3^*J* = 8.2 Hz, 1H, C7*H*), 7.77 (d, ^3^*J* = 8.7 Hz, 1H, C5*H*), 7.59 (d, ^3^*J* = 8.5 Hz, 1H, C20*H*), 7.56 (d, ^3^*J* = 7.4 Hz, 1H, C18*H*), 7.54 (d, ^3^*J* = 8.1 Hz, 1H, C10*H*), 7.51 (ddd, ^3^*J* = 8.7 Hz, 6.8 Hz, ^4^*J* = 1.2 Hz, 1H, C8*H*), 7.45 (d, ^3^*J* = 8.4 Hz, 1H, C14*H*), 7.45 – 7.43 (m, 1H, C19*H*), 7.41 (dd, ^3^*J* = 7.5 Hz, ^4^*J* = 2.1 Hz, 1H, C25`*H*), 7.39 (ddd, ^3^*J* = 8.3 Hz, 6.8 Hz, ^4^*J* = 1.3 Hz, 1H, C9*H*), 7.32 (dd, ^3^*J* = 8.0 Hz, ^4^*J* = 1.8 Hz, 1H, C24`*H*), 7.30 (s, 2H, C28*H*), 7.15 (dd, ^3^*J* = 8.0 Hz, ^4^*J* = 2.1 Hz, 1H, C25*H*), 6.97 (dd, ^3^*J* = 8.0 Hz, ^4^*J* = 1.8 Hz, 1H, C24*H*), 3.68 (s, 3H, C1*H*), 2.60 (s, 3H, C30*H*); ^13^C NMR hua-444_c (126 MHz, CDCl_3_) δ [ppm] = 167.9 (*C*1), 164.7 (*C*35), 149.0 (*C*36), 144.9 (*C*38), 143.3 (*C*27), 142.1 (*C*12), 139.3 (*C*23), 137.5 (*C*29), 137.31 (*C*22), 137.27 (*C*26), 135.8 (*C*13), 135.0 (*C*34), 134.8 (*C*6), 133.3 (*C*16), 133.0 (*C*11), 132.8 (*C*21), 131.1 (*C*24`), 130.4 (*C*24), 128.4 (*C*17), 128.3 (*C*10), 128.2 (*C*14), 128.0 (*C*7), 127.8 (*C*3), 127.7 (*C*5), 127.66 (*C*8), 127.5 (*C*15), 126.8 (*C*28), 126.7 (*C*20), 126.6 (*C*9), 126.4 (*C*19), 126.3 (*C*25`), 126.2 (*C*25), 125.96 (*C*18), 125.8 (*C*4), 124.1 (*C*31), 119.7 (*C*33), 119.2 (*C*37), 52.2 (*C*1), 18.9 (*C*30); ^19^F NMR (376 MHz, CDCl_3_) δ [ppm] = ‑64.6 (s, 6F, C34*F*); ESI-MS: m/z calcd. for C_48_H_31_F_6_N_8_O_2_Pt 1060.2117 found 1060.2101 [M–H^-^] Anal. Calcd. For C_48_H_30_F_6_N_8_O_2_Pt: C, 54.39; H, 2.85; N, 10.57. Found: C, 47.45; H, 2.81; N, 10.58.

#### 3.2.2 **Pt(trzpy)(monodent-RNC) (9)**

**Na[Pt(trzpy)Cl]** (28.4 mg, 0.05 mmol, 1.0 eq.) was combined with **monodent‑RNC** (13.2 mg, 0.04 mmol, 1.0 eq) and AgPF_6_ (12.9 mg, 0.05 mmol, 1.2 eq.) in methanol (2.5 mL). The mixture was stirred at 65 °C under Ar atmosphere. After stirring for 48 h, LCMS confirmed full conversion of the ligand. The reaction mixture was filtered over a syringe filter and concentrated under reduced pressure. The compound was purified by column chromatography (silica, 0 - 100% EtOAc in cyclohexane) to give a yellow solid (**9**, 22.8 mg, 0.03 mmol, 65%).

ν_max_ (neat) = 2967w, 2361w, 2214m, 1596m, 1478m, 1157s, 745s; ^1^H NMR (400 MHz, CDCl_3_) δ [ppm] = 8.14 (dd, ^3^*J* = 7.9 Hz, 1H), 7.89 (d, ^3^*J* = 7.9 Hz, 2H), 7.43 (s, 2H), 6.69 (d, ^3^*J* = 2.2 Hz, 2H), 6.52 (ddd, ^4^*J* = 2.2 Hz, 1H), 3.87 (s, 6H), 1.42 (s, 7H), 1.41 (s, 6H); ESI-MS: m/z calcd. for C_34_H_32_F_6_N_9_O_2_Pt^+^ 907.2228 found 907.2215 [M+MeCN]; Anal. Calcd. For C_32_H_28_F_6_N_8_O_2_Pt: C, 44.40; H, 3.26; N, 12.94. Found: C, 46.53; H, 4.15; N, 11.47.

## 4. Additional photophysical data


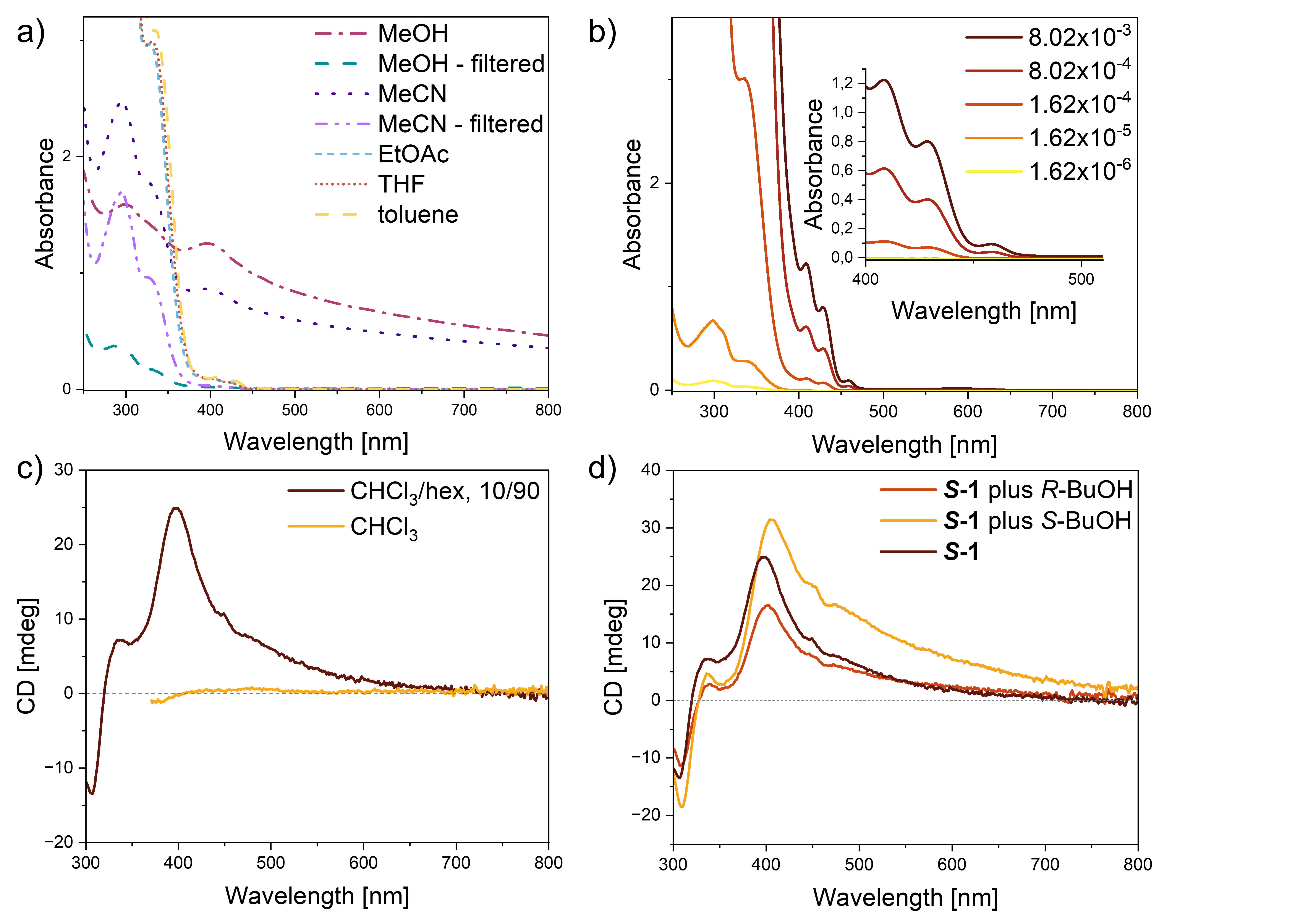


**Figure S4.** a) UV-vis absorption spectra of complex **S-1** 2×10^-5^ M in different solvents and in CHCl_3_ at given concentrations (b). CD spectra of **S-1** 2×10^-5^ M in CHCl_3_ (c) and in CHCl_3_/n-hexane, 1/9 (d), upon addition of excess R/S 2-BuOH (0.55 mmol) (d).


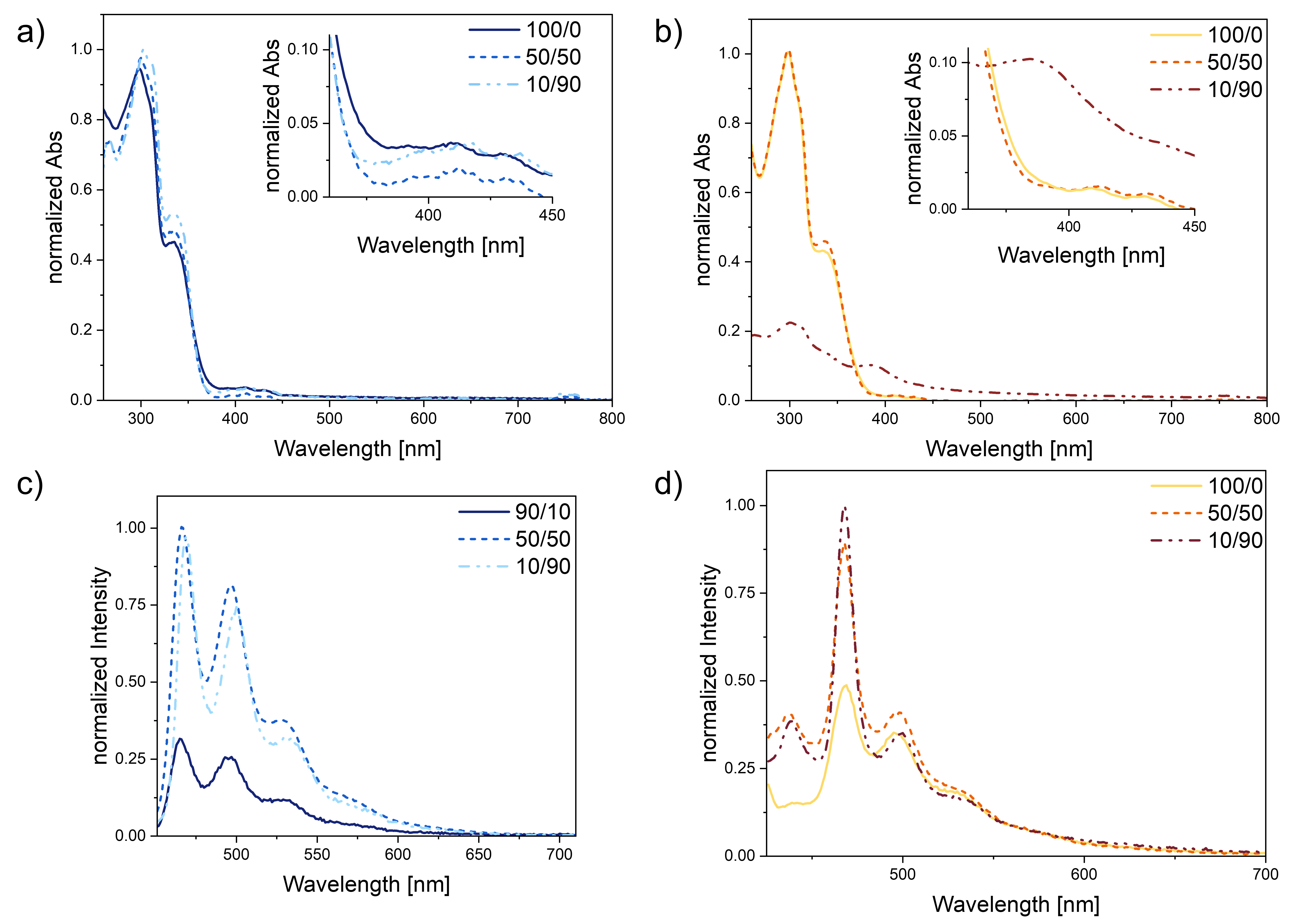


**Figure S5.** UV-vis absorption spectra of reference complex **9** (a) and **S-1** (b)
2×10^-5^ M in given ratios of CHCl_3_/n-hexane. Steady state emission spectra of **9** (c) and **S-1** (d) 2×10^-5^ M in given ratios of CHCl_3_/n-hexane.


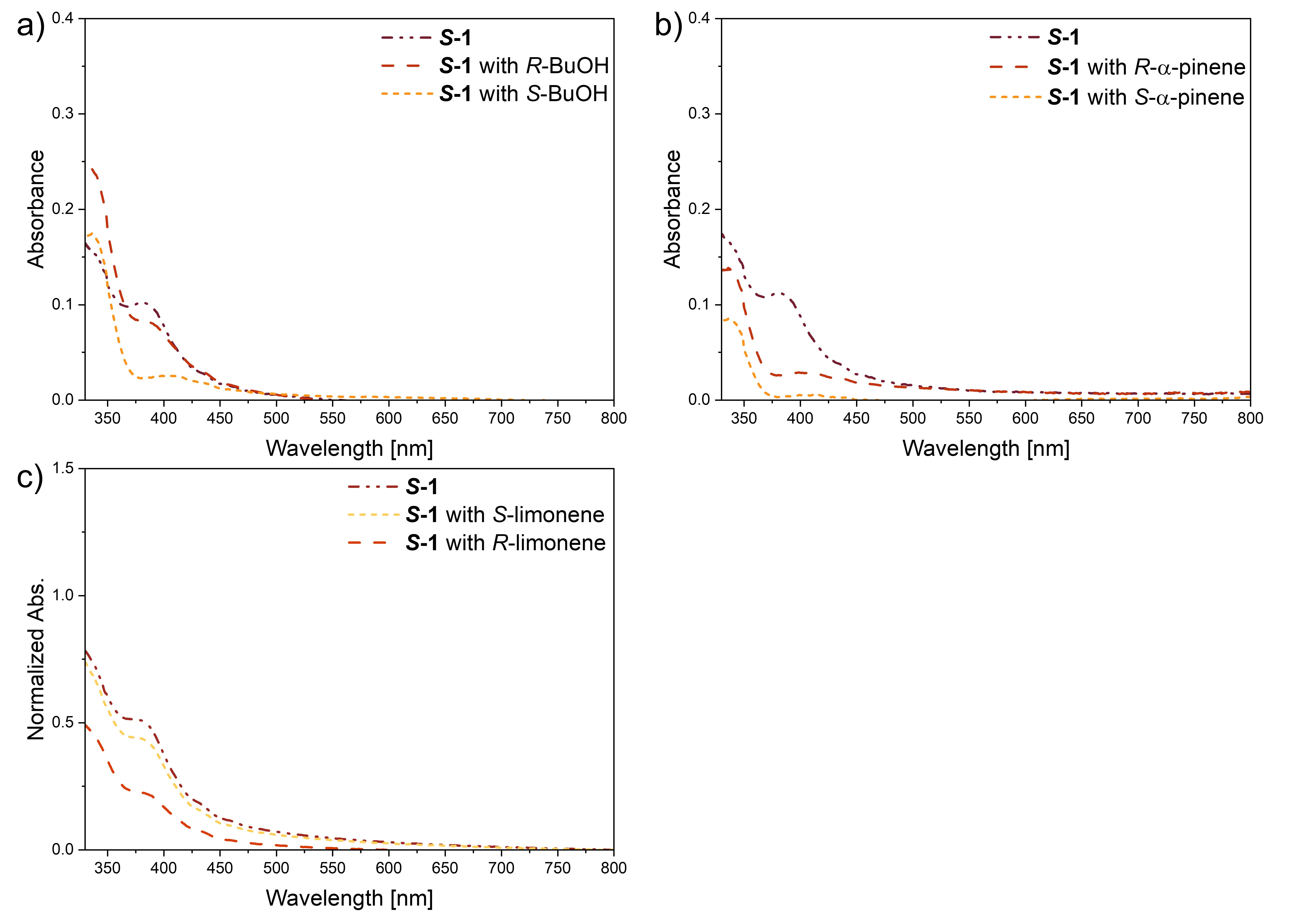


**Figure S6.** UV-vis absorption spectra of aggregated **S-1**, 2×10^-5^ M in CHCl_3_/n-hexane, 1/9, upon addition of excess R/S-enantiomers of 2-butanol (a), α-pinene (b), and limonene (c).


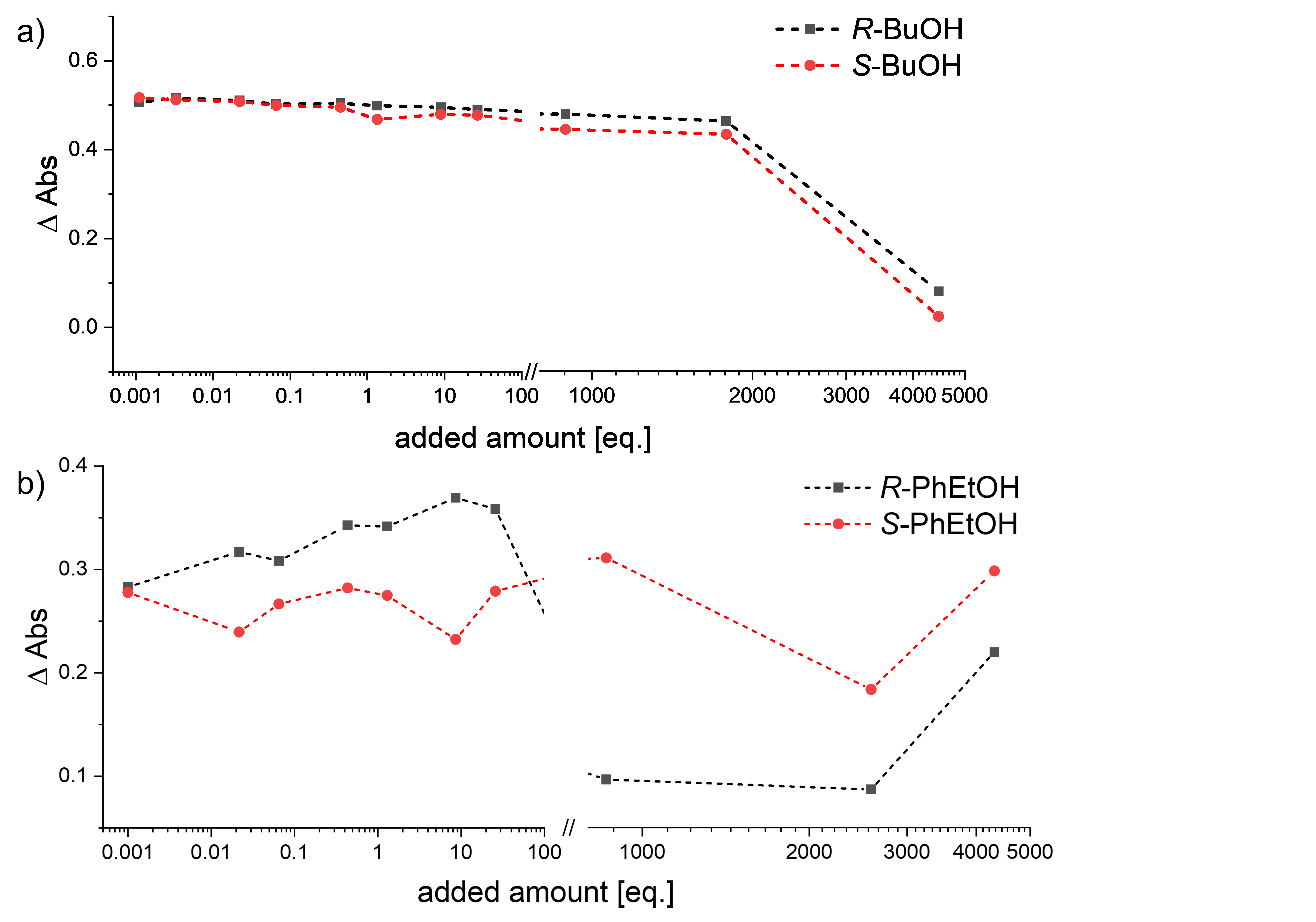


**Figure S7.** Representation of absorbance changes of **S-1** (2×10^-5^ M in CHCl_3_/n-hexane, 1/9) at 392 nm upon addition of given amounts R/S-2-BuOH (a) and R/S-1-PhEtOH (b) displayed on a logarithmic x-axis. The LOD values for 2-BuOH and PhEtOH were 1.34 equiv. (corresponding to 1.3 × 10^-5^ M) and 0.022 equiv. (corresponding to 2.2 × 10^-7^ M), respectively.


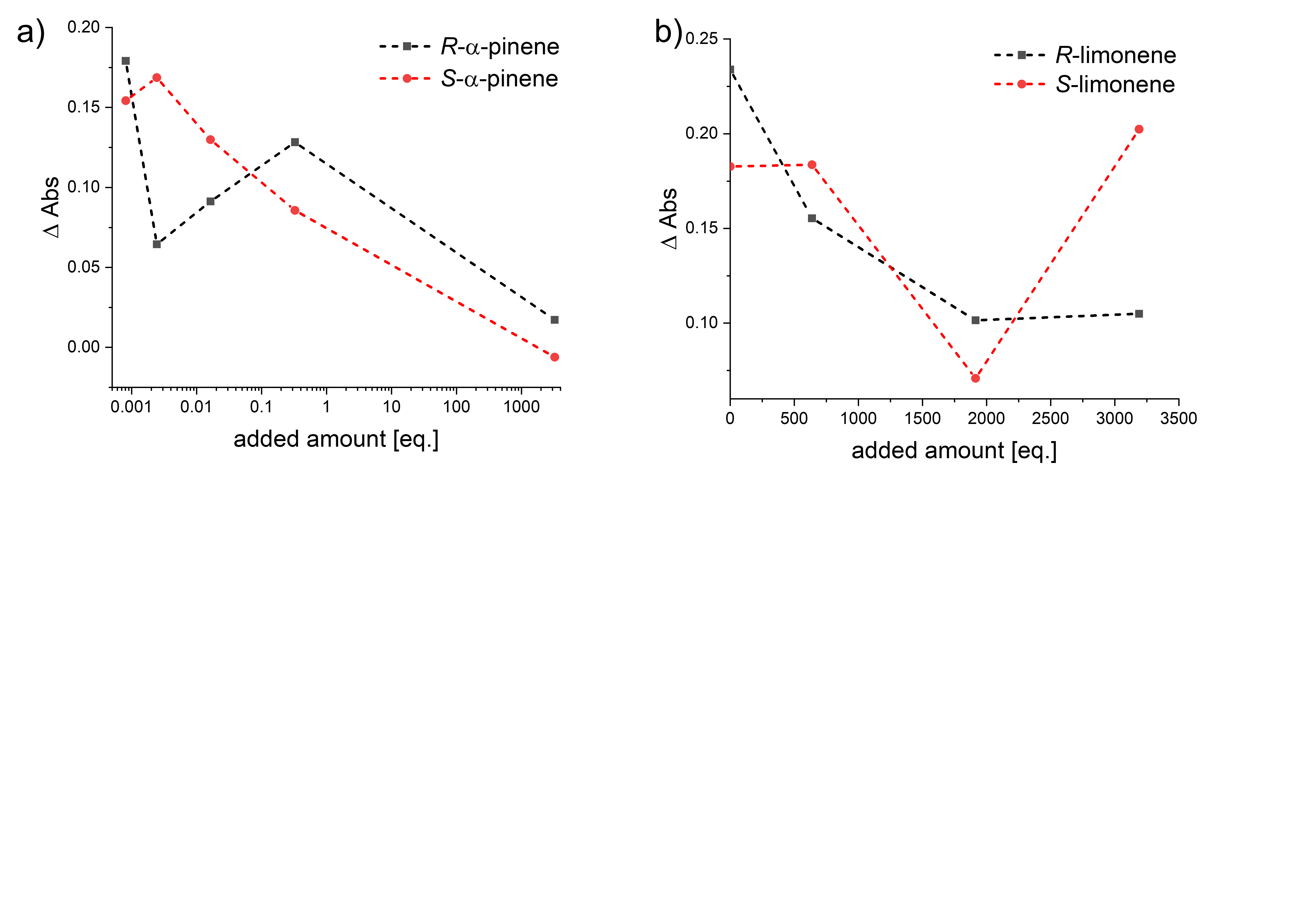


**Figure S8.** Representation of absorbance changes of **S-1** (2×10^-5^ M in CHCl_3_/n-hexane, 1/9) at 392 nm upon addition of given amounts R/S-α-pinene (a) and R/S limonene (b) displayed on a logarithmic x-axis. The LOD values for pinene and limonene were both at 0.0024 equiv., which corresponds to 2.4×10^-8^ M.


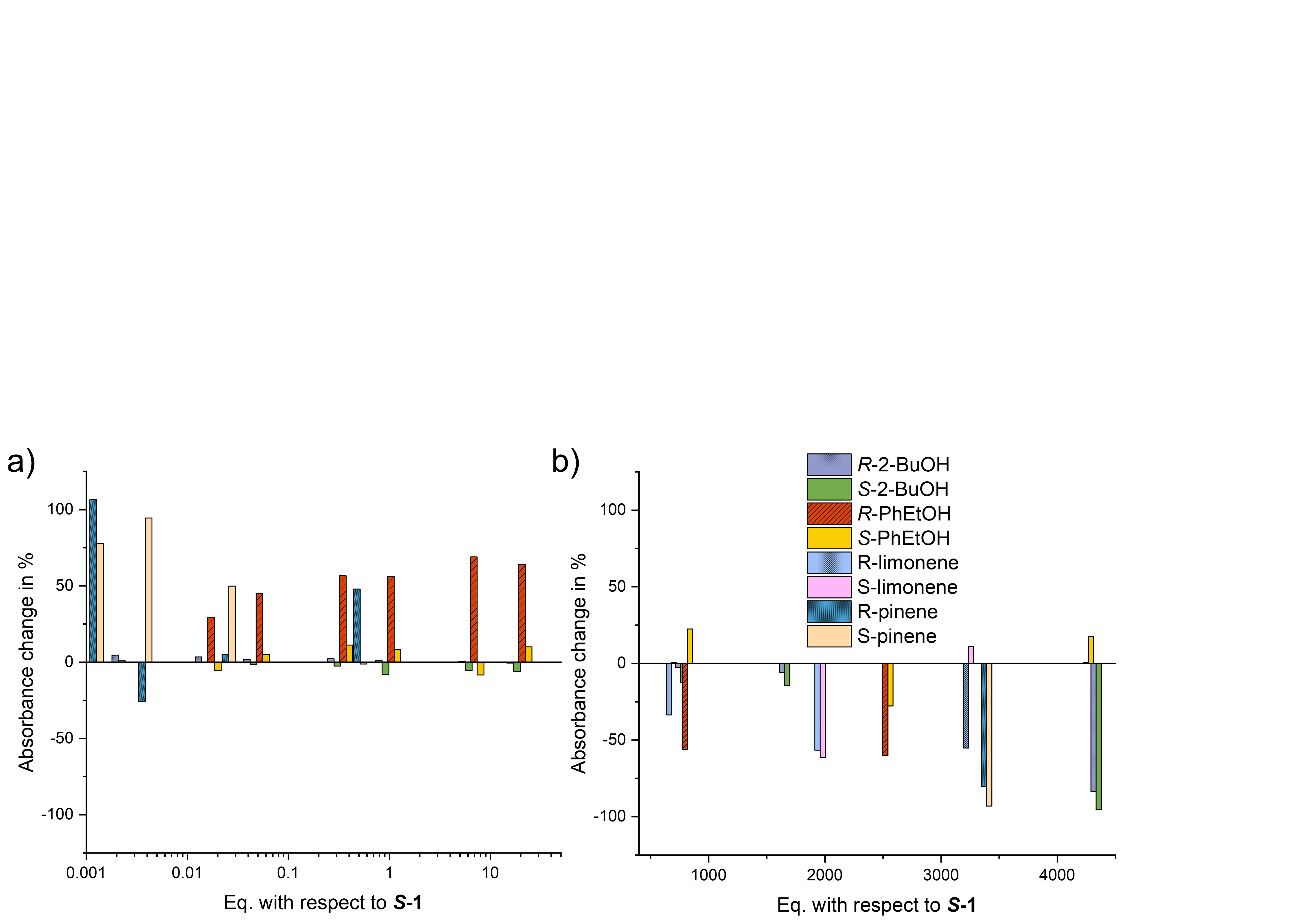


**Figure S9.** Percentual absorbance changes of **S-1** upon addition of given analyte amounts a) on a logarithmic scale up to 20 eq. and b) on a linear scale for large analyte excess.

## 5. Scanning electron microscope (SEM) images of ***S*-1**

a) b)


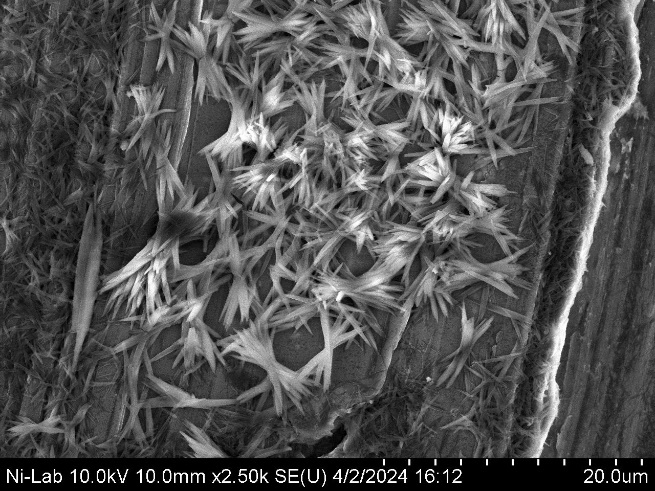

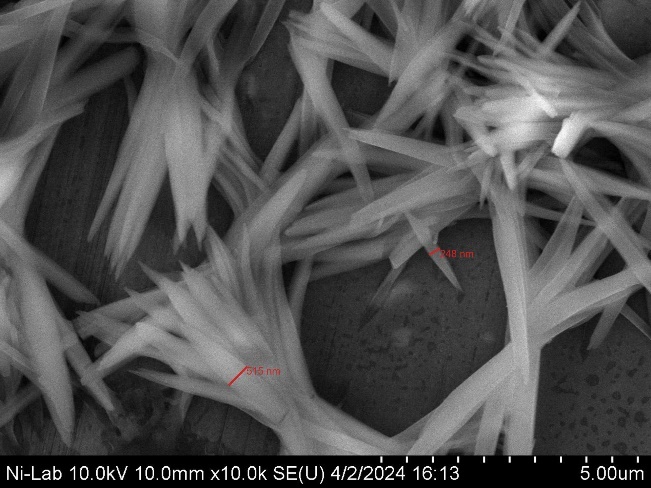


**Figure S10.** SEM images of complex **S-1** developed from a CHCl_3_ solution (1×10^-3^ M) drop-casted onto an Al-carrier. Scale bars are indicated on the bottom right of each image.

a) b)


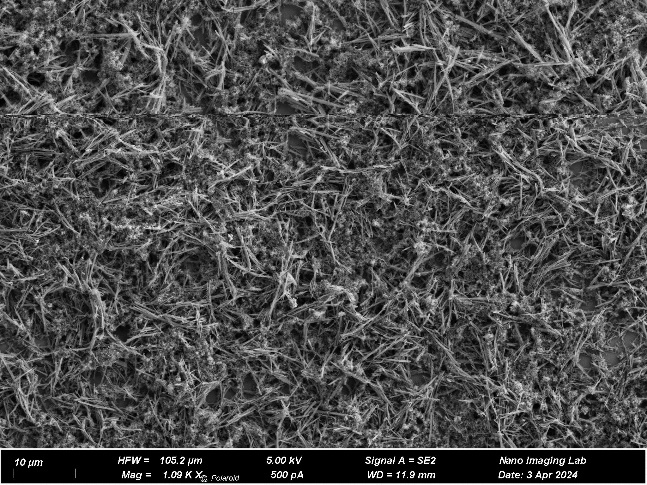

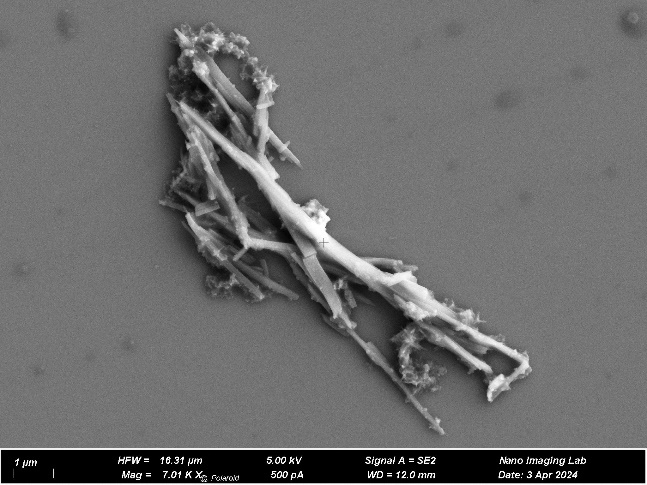


**Figure S11.** SEM images of **S-1** developed from a CHCl_3_/n-hexane suspension (1×10^-3^ M) drop-casted onto a Si-carrier (a) and more diluted on a Si carrier (b). Scale bars are indicated on the bottom left of each image.

a) b)


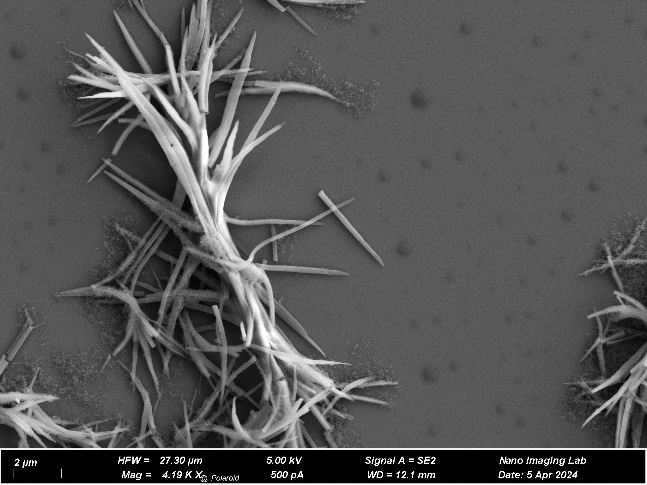

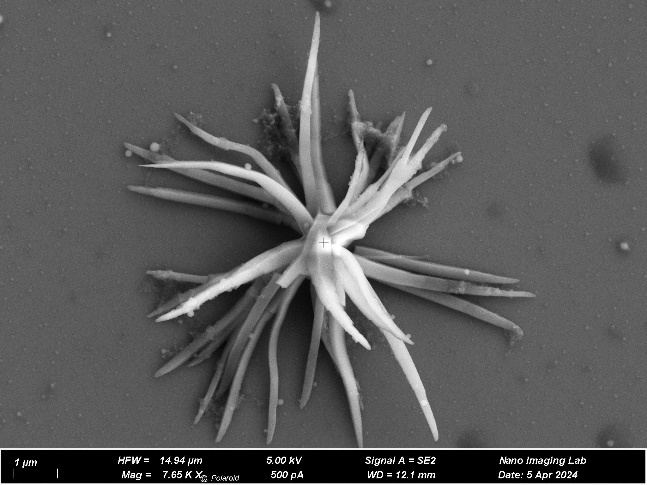


**Figure S12.** SEM images of ***S*-1** developed from a CHCl_3_/*n*-hexane suspension
(1×10^-5^ M) drop-casted onto a Si-carrier. Scale bars are indicated on the bottom left of each image.

## 6. ^1^H NMR comparisons

### 6.1 NMR titrations of aggregated ***S*-1** with *R*/*S* 2-BuOH


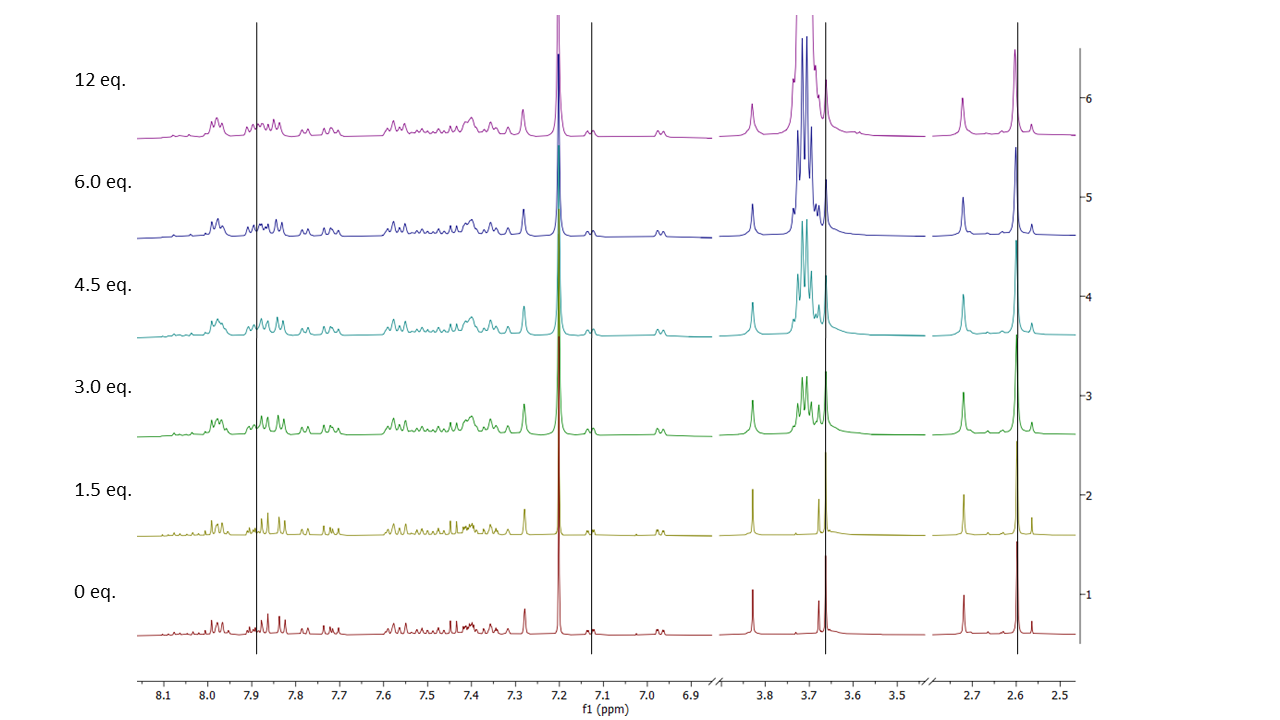


**Figure S13.** ^1^H NMR spectra of complex **S-1** in CHCl_3_/C_6_D_12_, 1/1 (bottom) with increasing amounts of R-BuOH (1.5, 3.0, 4.5, 6.0, 12 eq.) displayed from bottom up.


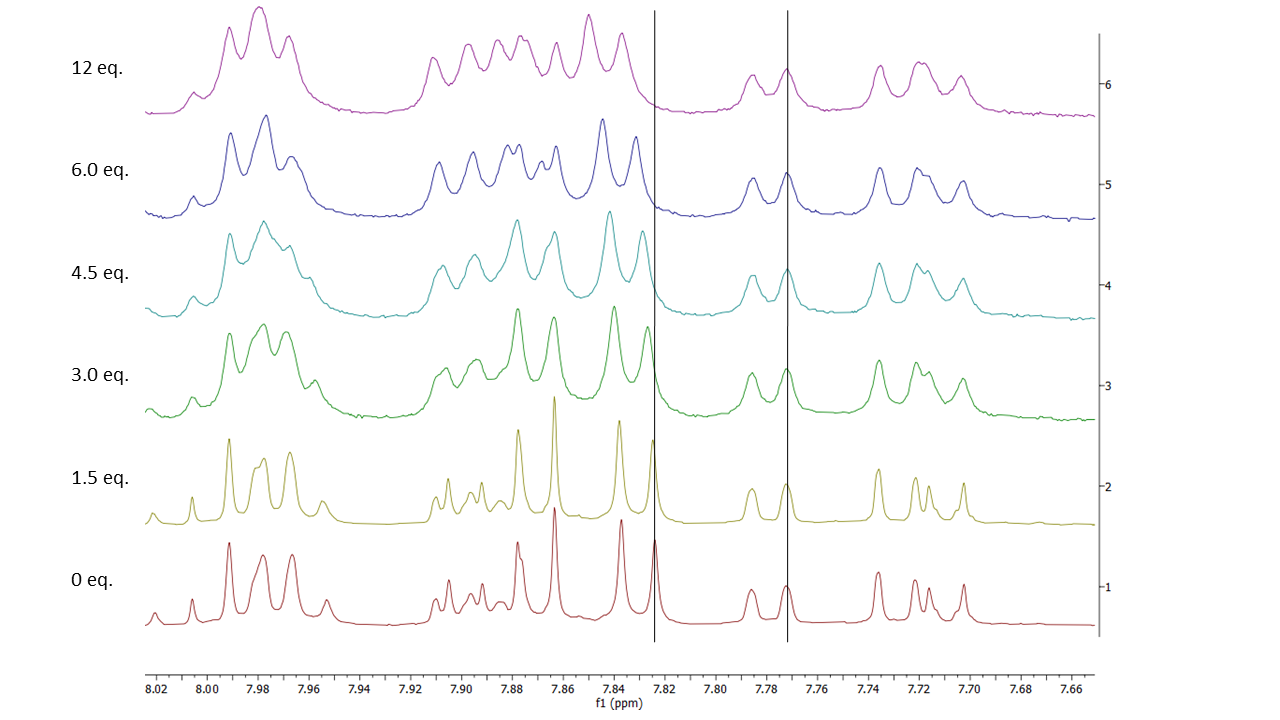


**Figure S14.** Inset of the ^1^H NMR spectra of **S-1** in CHCl_3_/C_6_D_12_ in the range of 8.02 – 7.65 ppm showing the titration of R-BuOH (0, 1.5, 3, 4.5, 6, 12 in eq.) from bottom to top. The black vertical lines help estimating how much the signals shift and broaden upon addition.

### 6.2 NMR spectra before- and after sensing and recovery


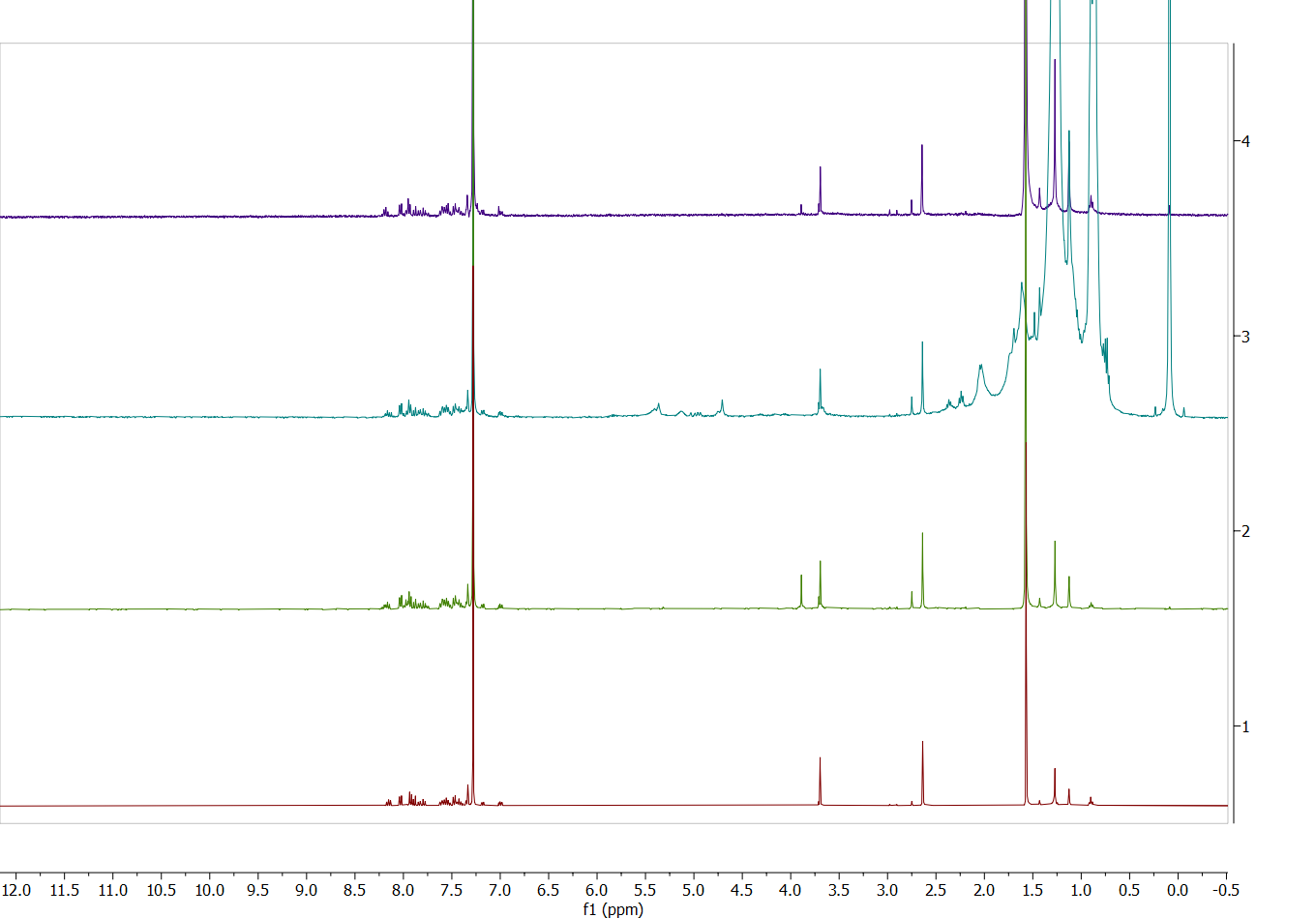


**Figure S15.** ^1^H NMR spectra of **S-1** isolated (1), recovered after sensing R/S 2-BuOH and R/S α-pinene from two reuse cycles (2). ^1^H NMR spectra of **S-1** after sensing R/S limonene in the third reuse cycle with residual analyte (3), and after sensing R/S limonene and being washed according to the method described in section 1.2, showing the start of the fourth reuse cycle.


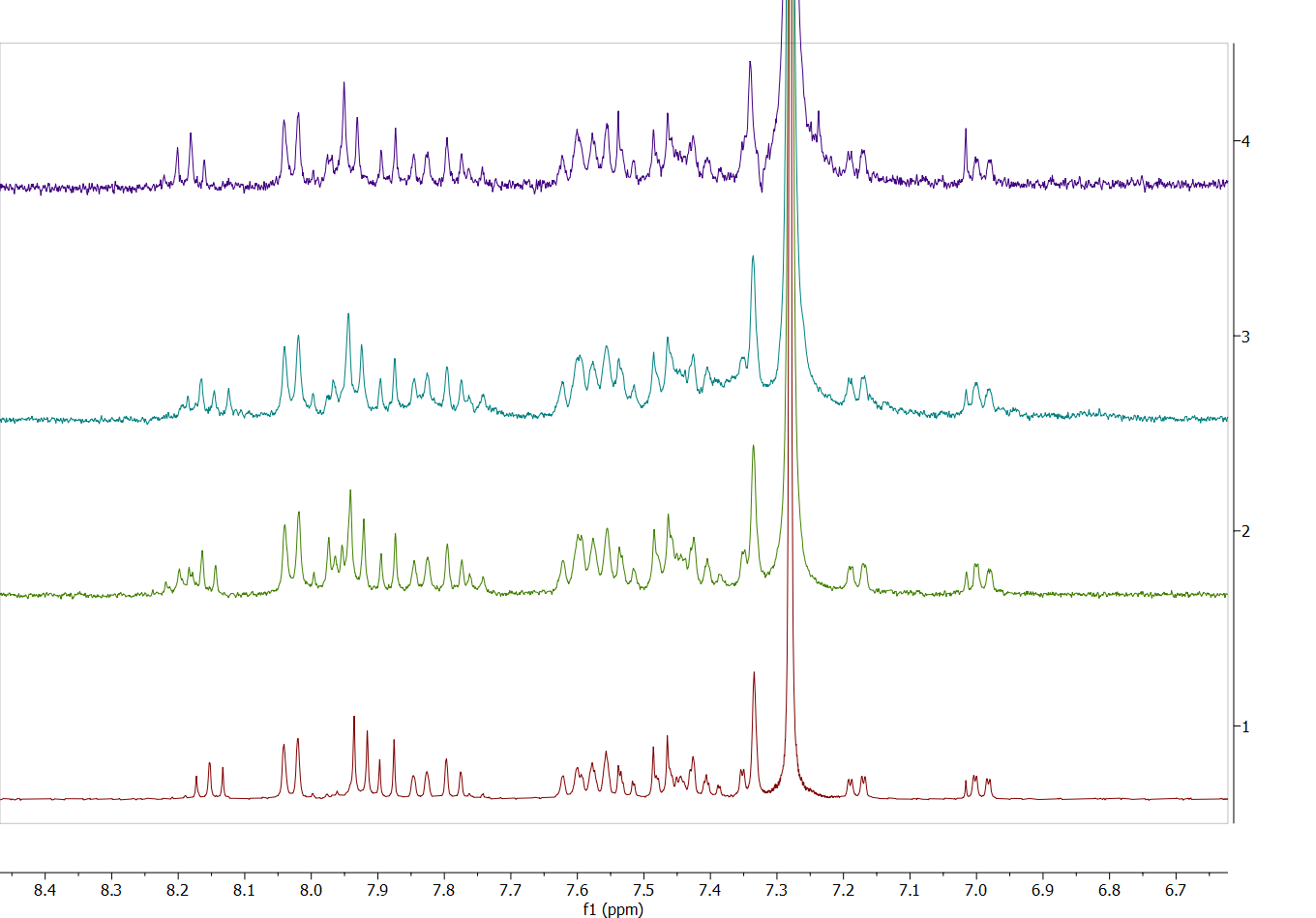


**Figure S16.** Aromatic region of the ^1^H NMR spectra of **S-1** isolated (1), recovered after sensing R/S 2-BuOH and R/S α-pinene from two reuse cycles (2). ^1^H NMR spectra of **S-1** after sensing R/S limonene in the third reuse cycle with residual analyte (3), and after sensing R/S limonene and being washed according to the method described in section 1.2, showing the start of the fourth reuse cycle.

## 7. NMR and HRMS data

### 7.1 All intermediates and the isocyanide ligand **8**

#### 7.1.1 **2**


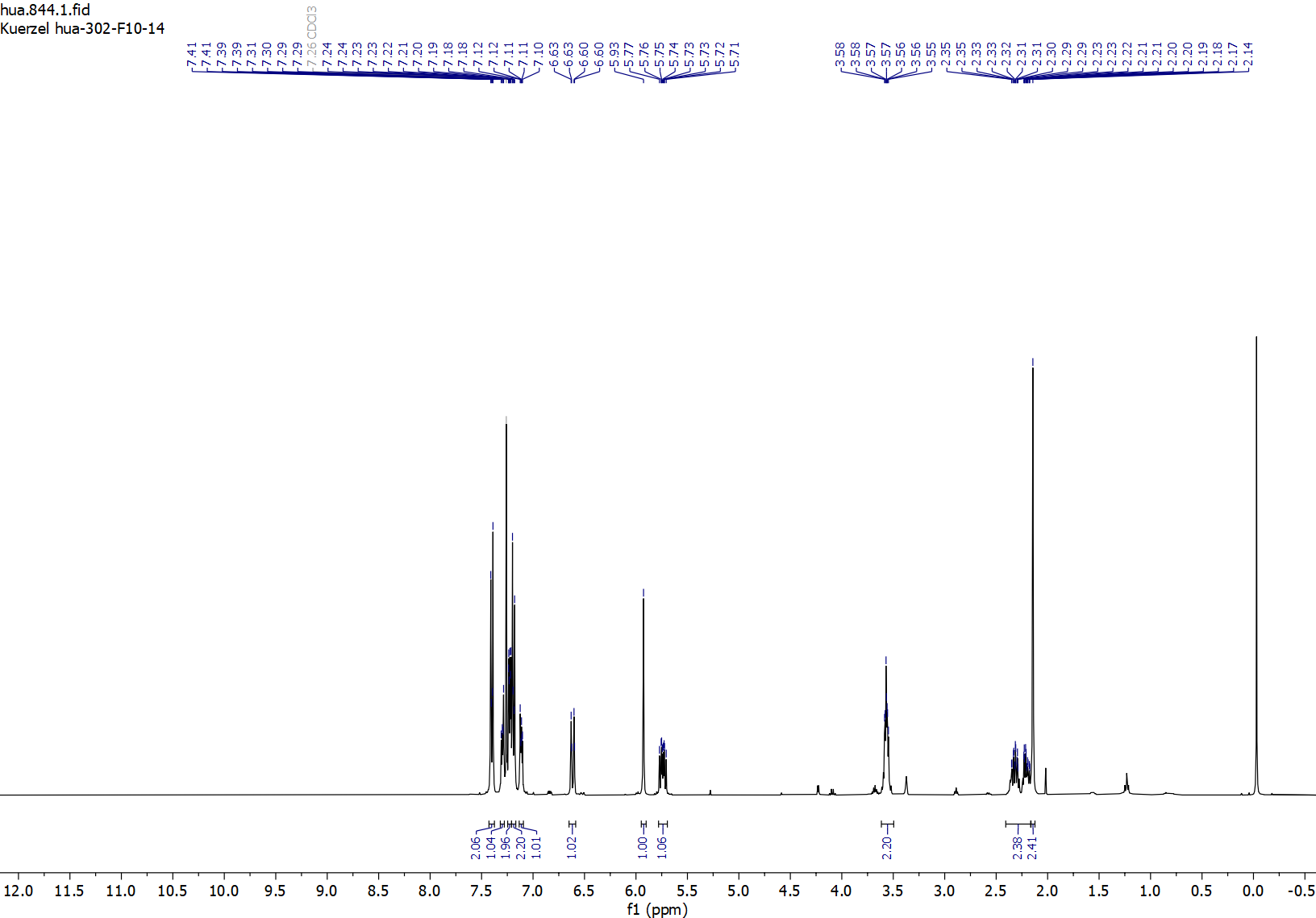


**Figure S17.** ^1^H NMR spectrum of **2** in CDCl_3_.


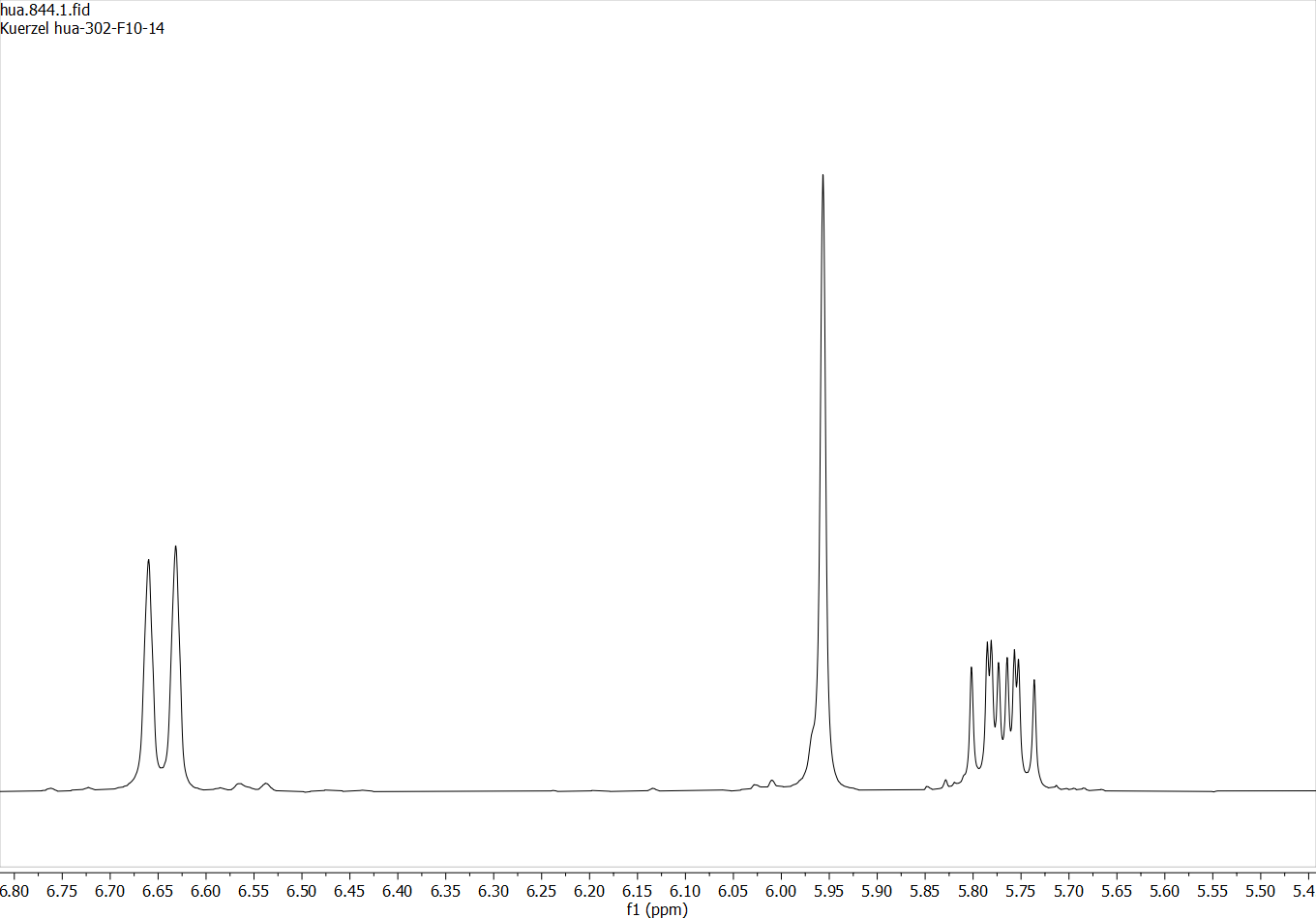


**Figure S18.** Focused ^1^H NMR spectrum of **2** in CDCl_3_.


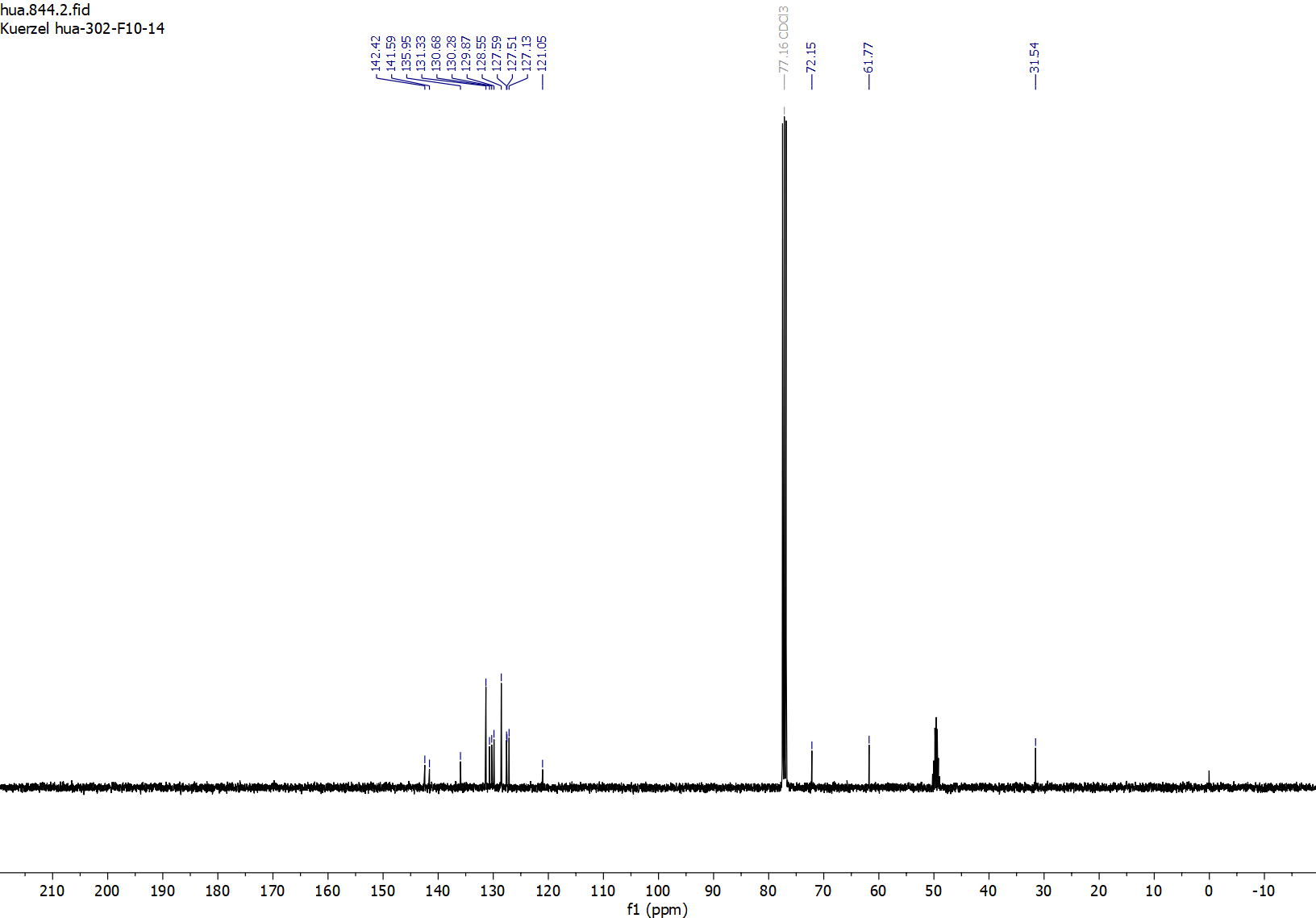


**Figure S19.** ^13^C NMR spectrum of **2** in CDCl_3_.


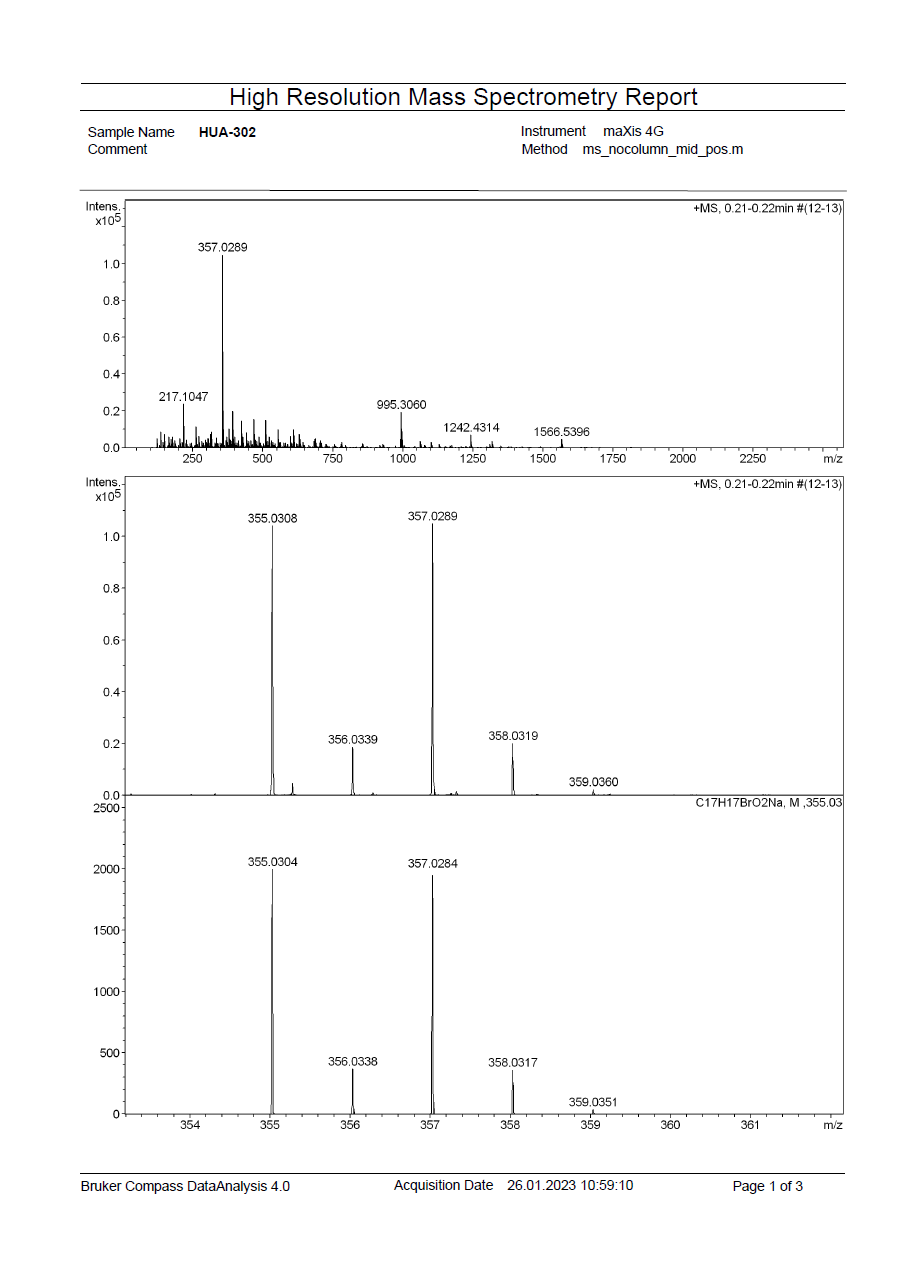


**Figure S20**. HRMS spectra of **2.**


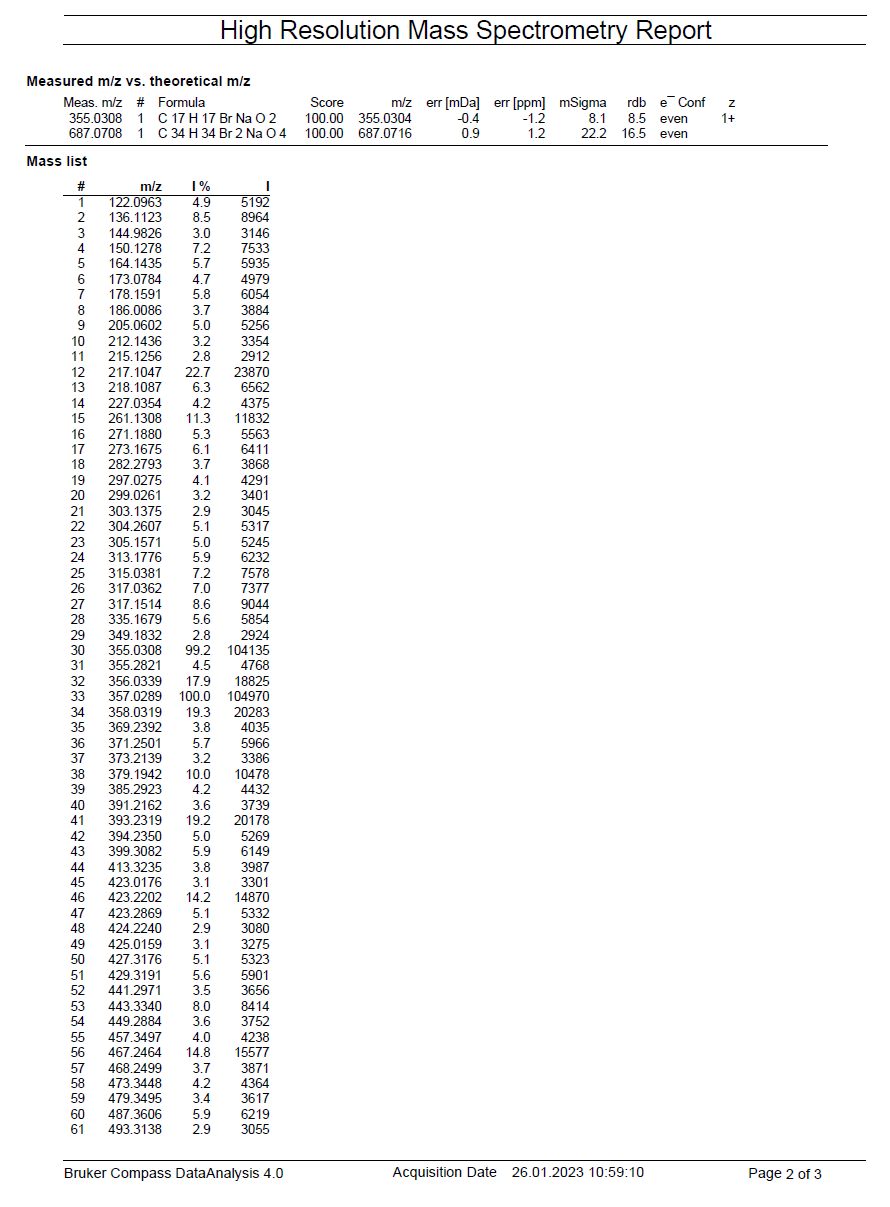


**Figure S21.** HRMS report of **2.**

#### 7.1.2 **3**


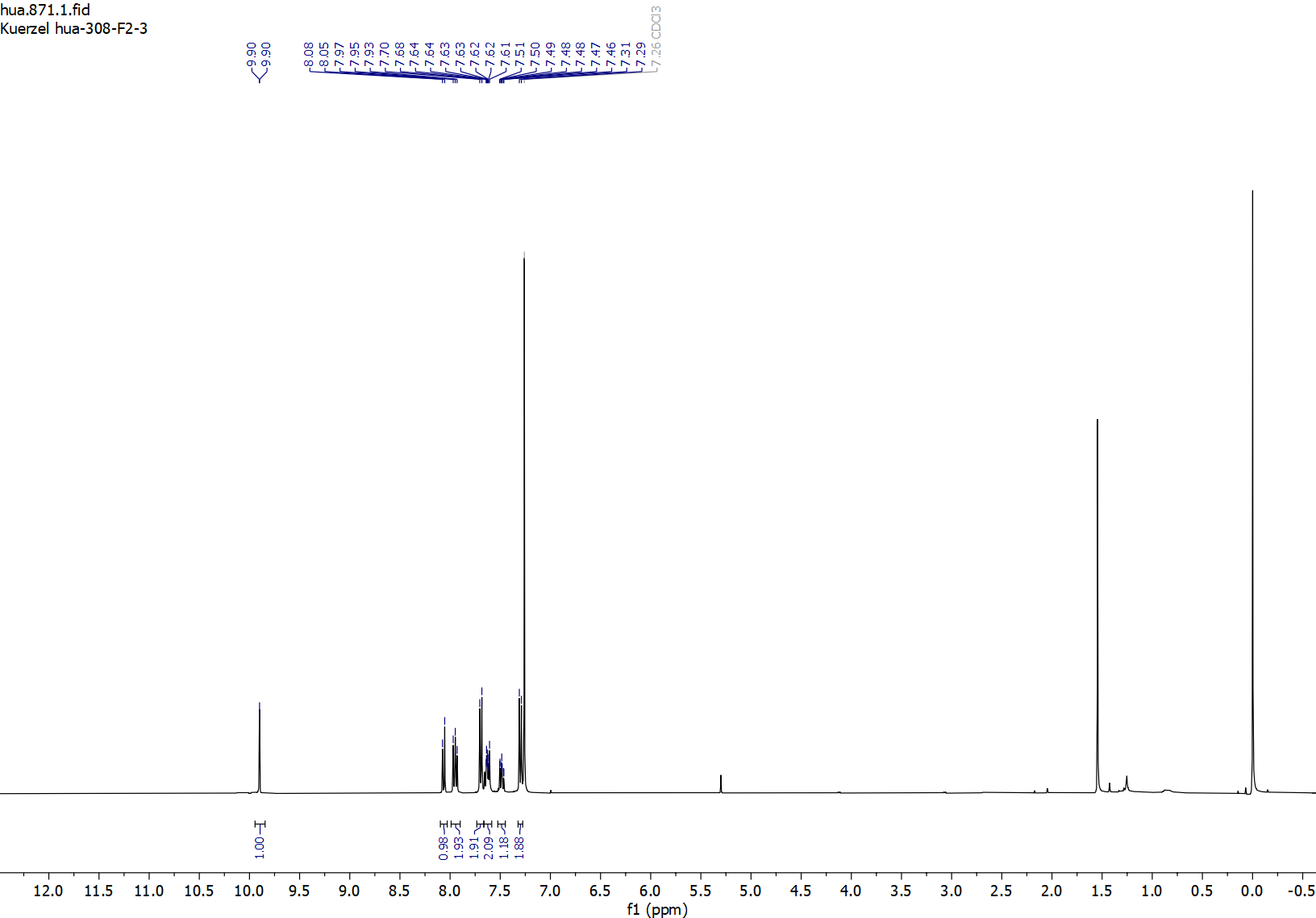


**Figure S22.** ^1^H NMR spectrum of **3** in CDCl_3_.


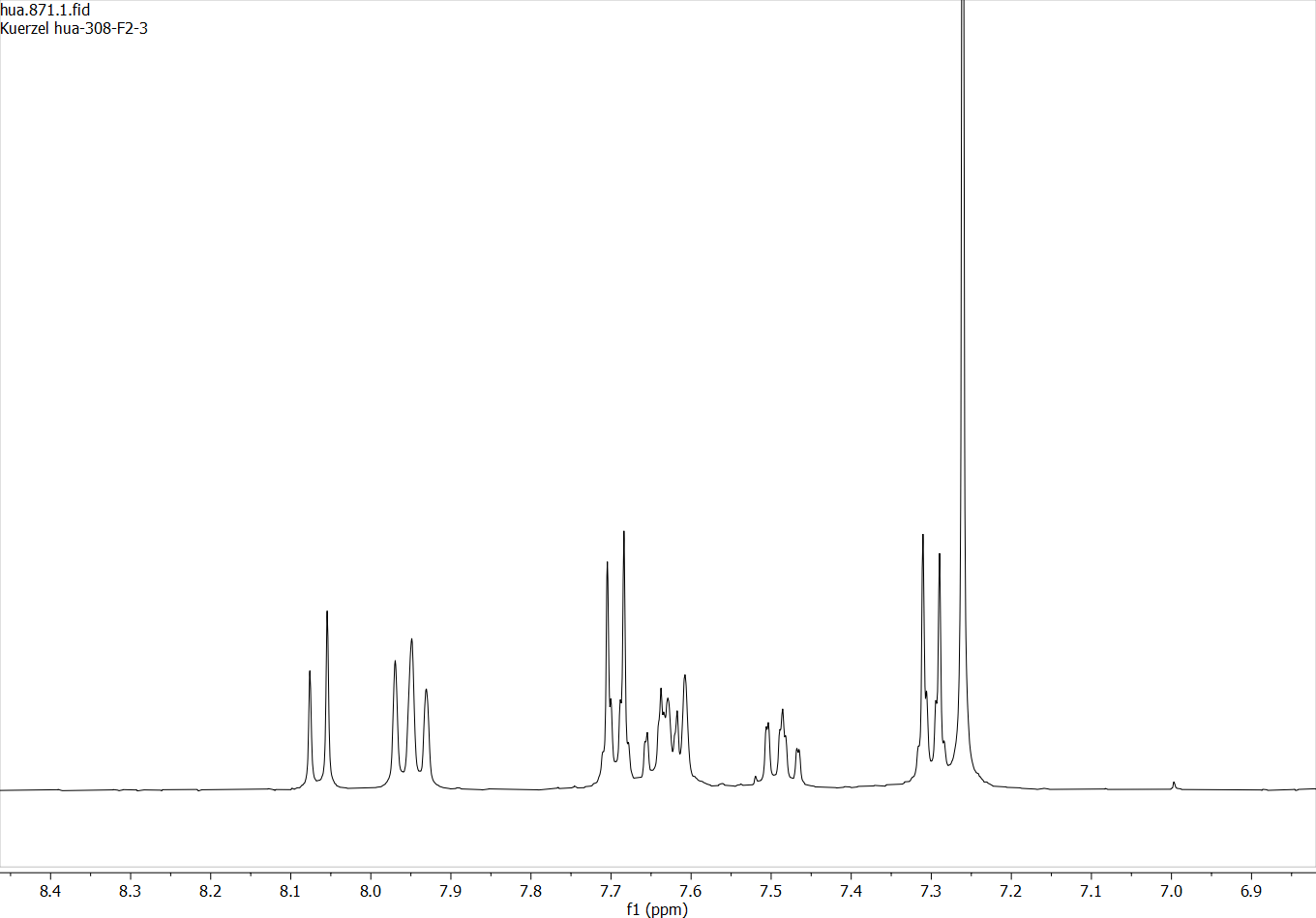


**Figure S23.** Aromatic region of the ^1^H NMR spectrum of **3** in CDCl_3_.


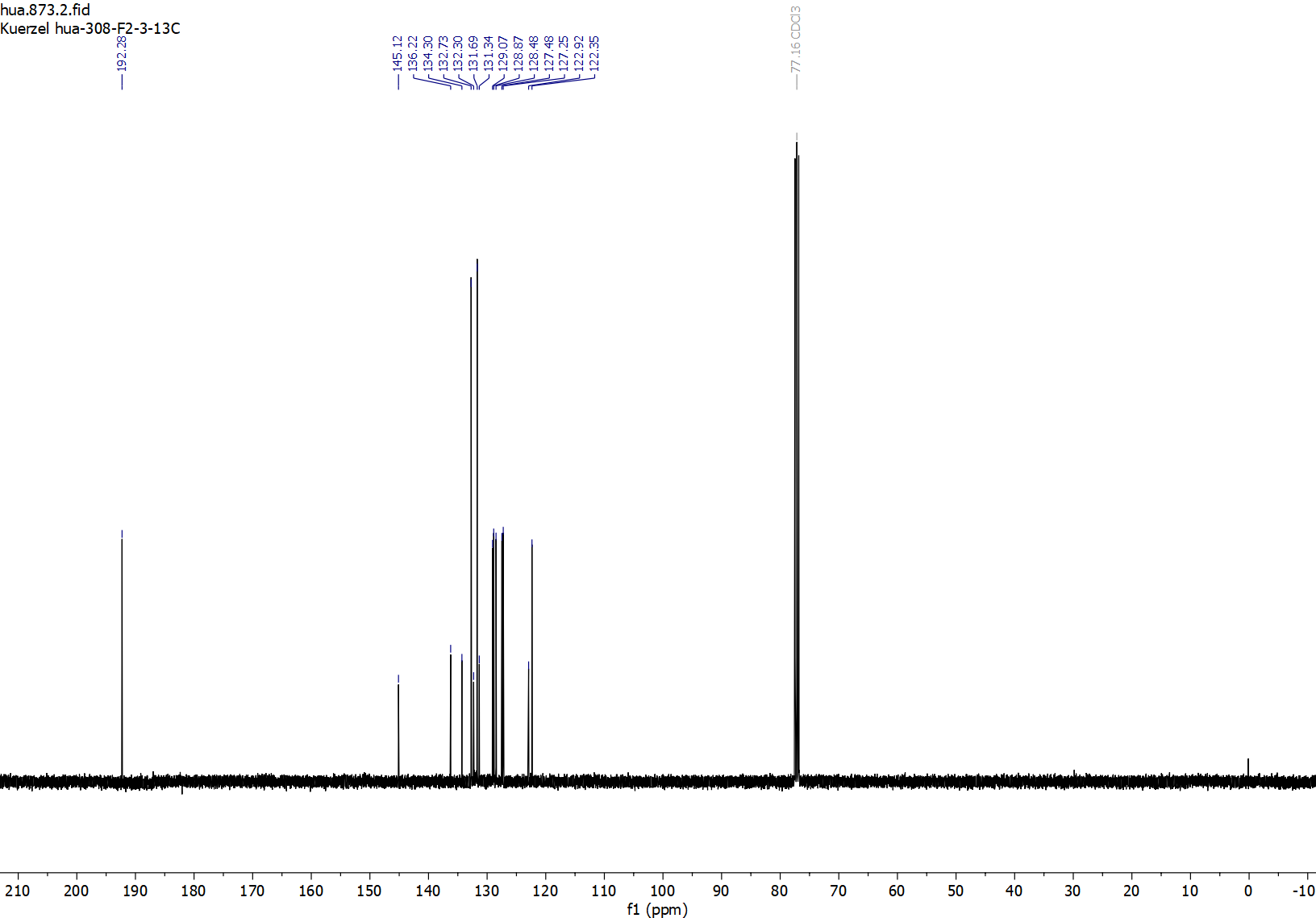


**Figure S24.** ^13^C NMR spectrum of **3** in CDCl_3_.


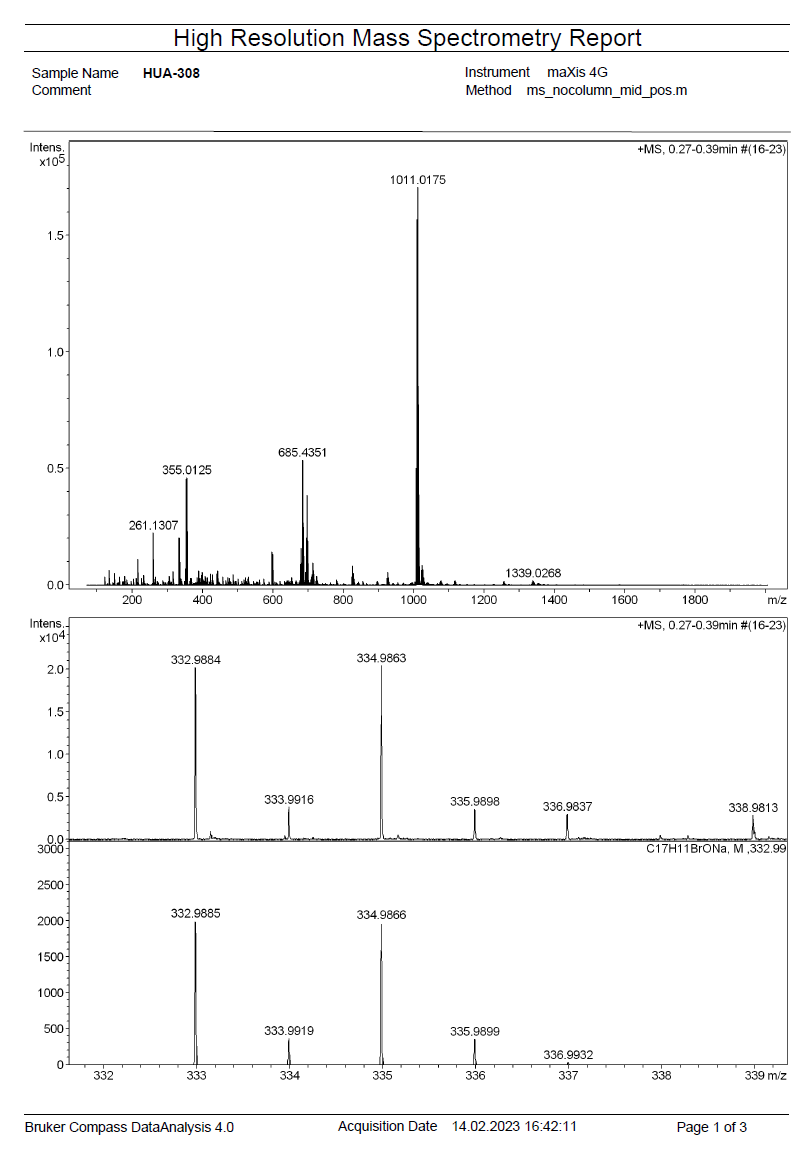


**Figure S25.** HRMS spectra of **3.**


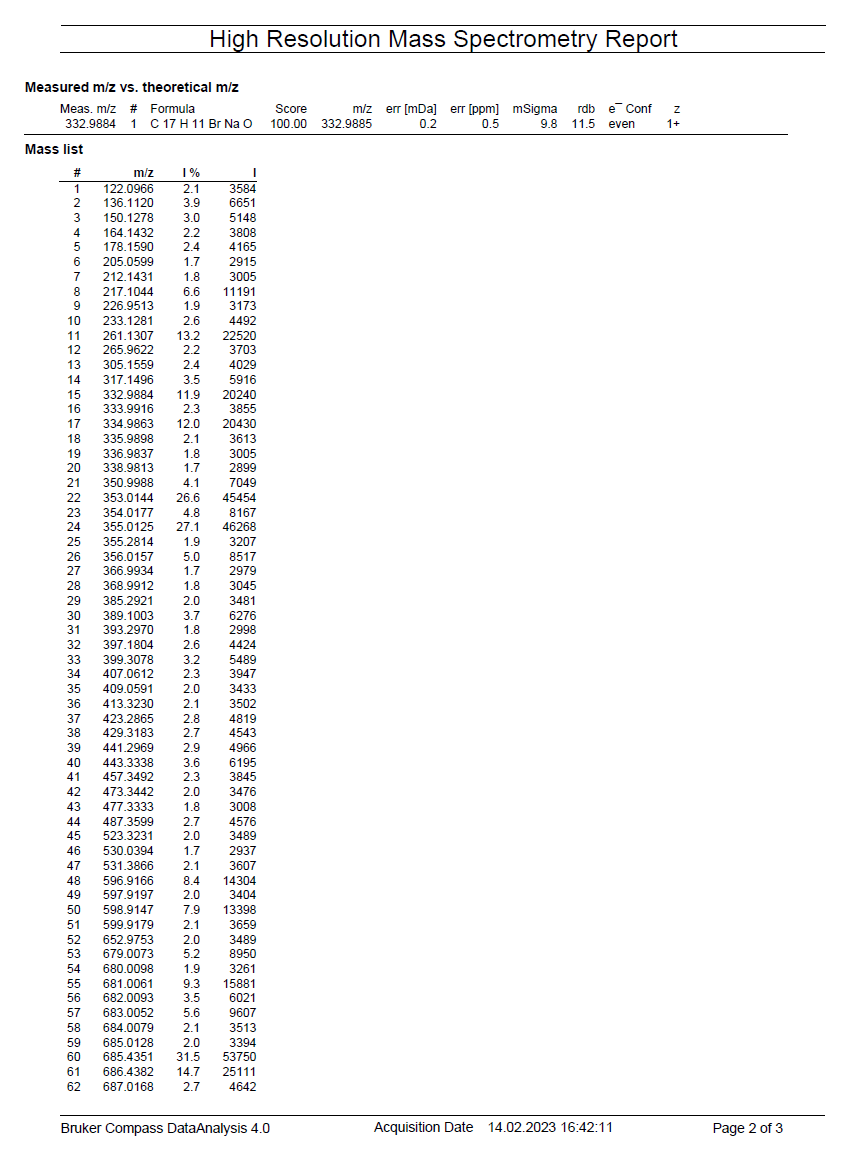


**Figure S26.** HRMS report of **3.**

#### 7.1.3 **4**


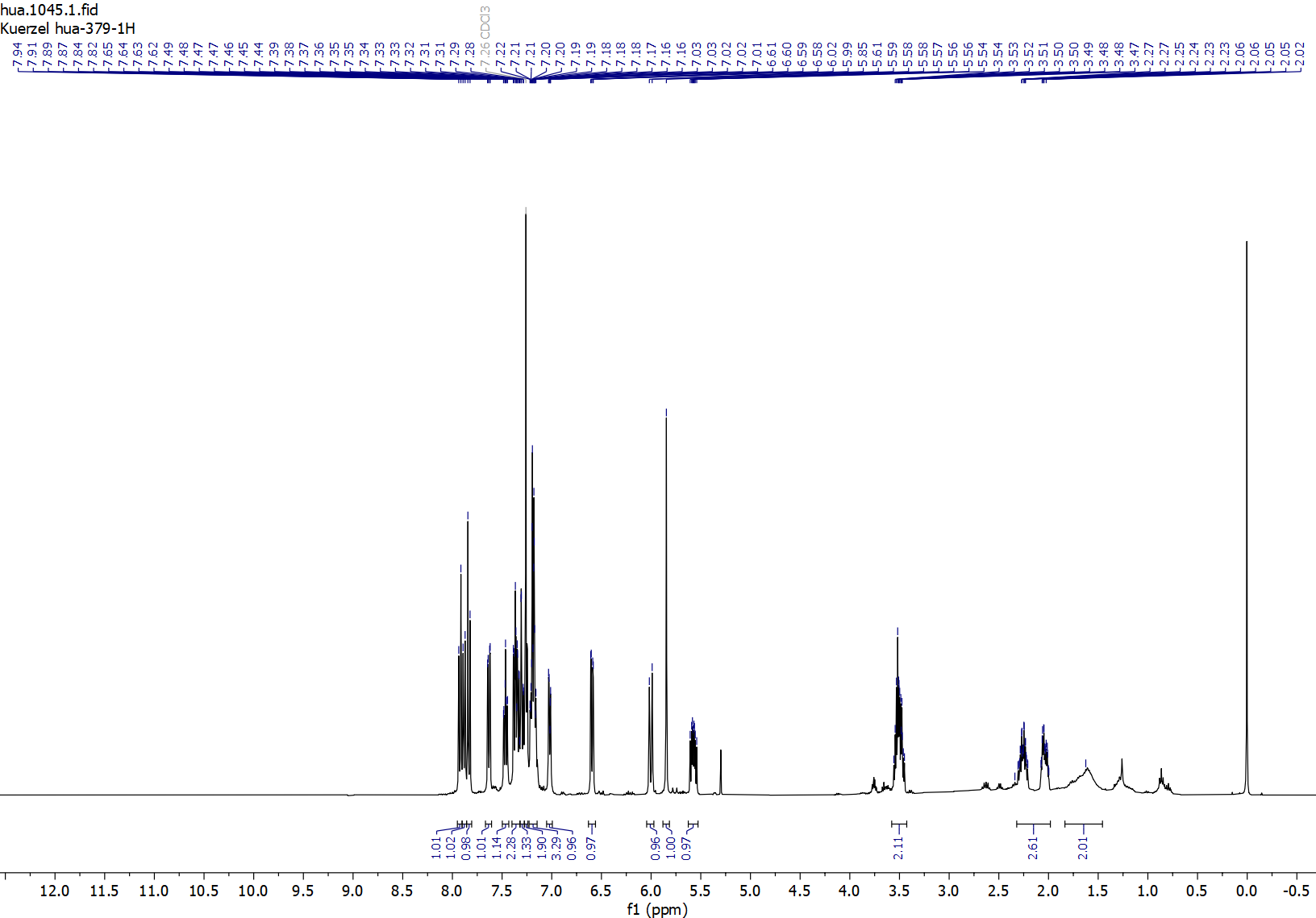


**Figure S27.** ^1^H NMR spectrum of **4** in CDCl_3_.


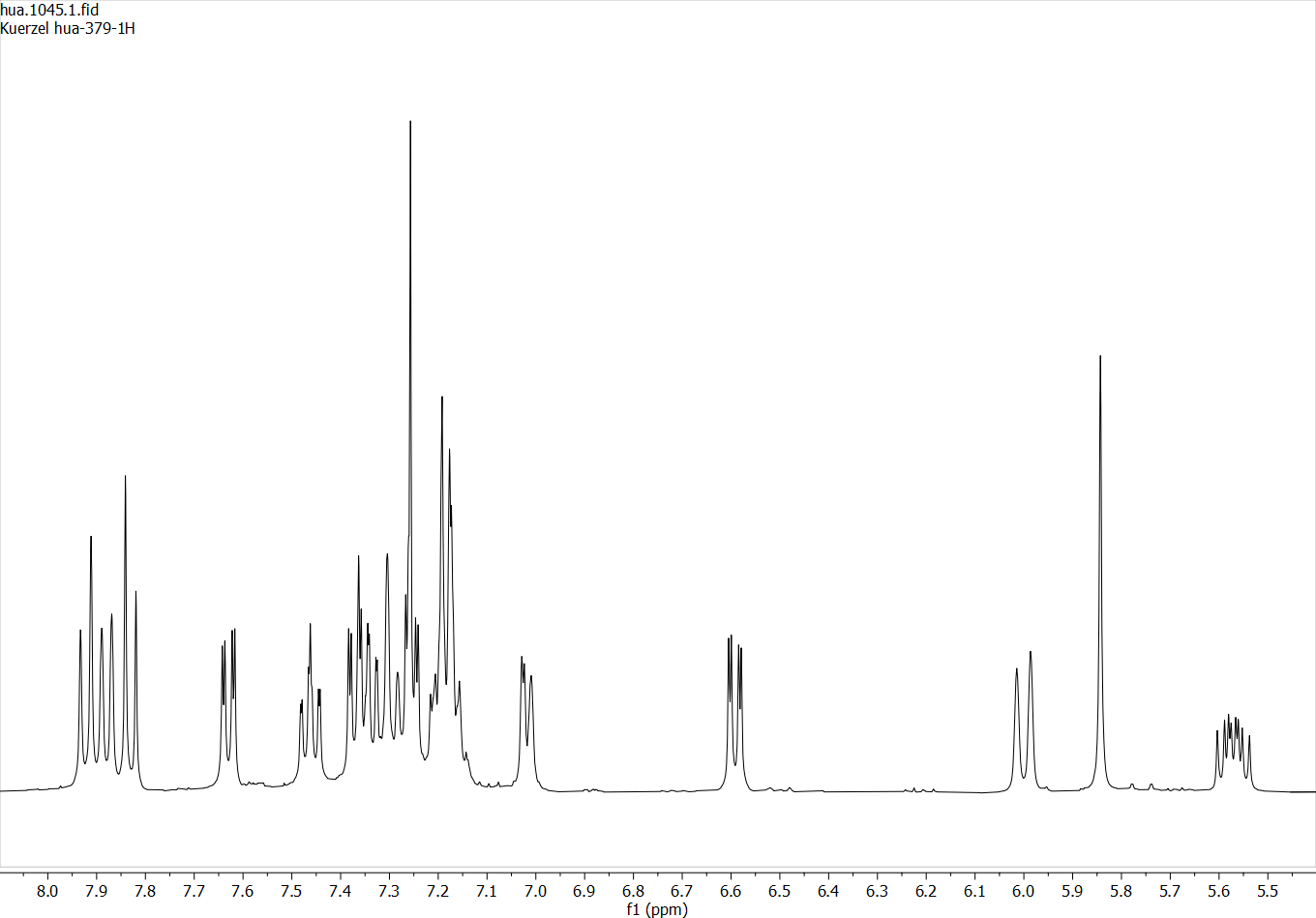


**Figure S28.** Aromatic region of the ^1^H NMR spectrum of **4** in CDCl_3_.


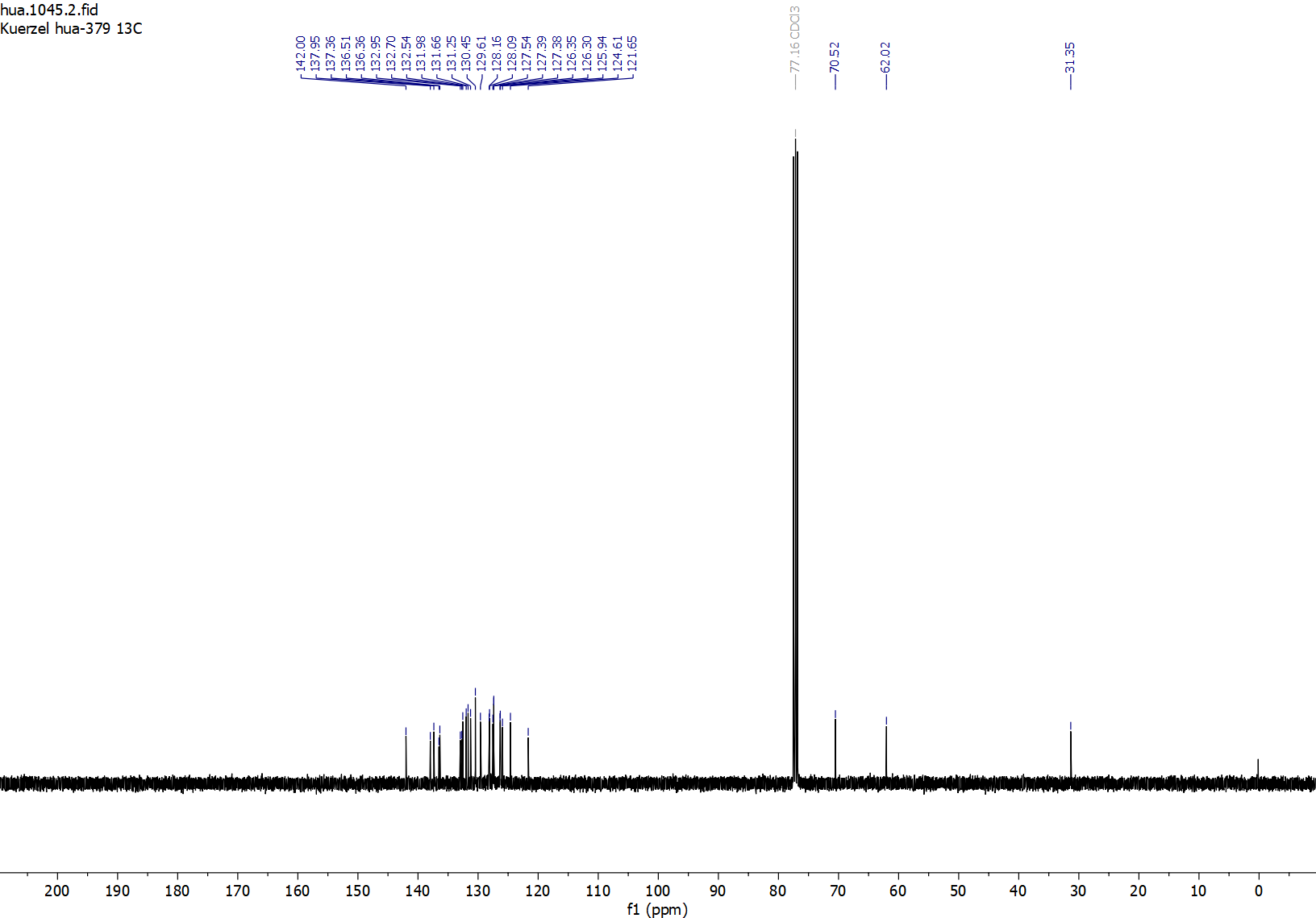


**Figure S29.** ^13^C NMR spectrum of **4** in CDCl_3_.


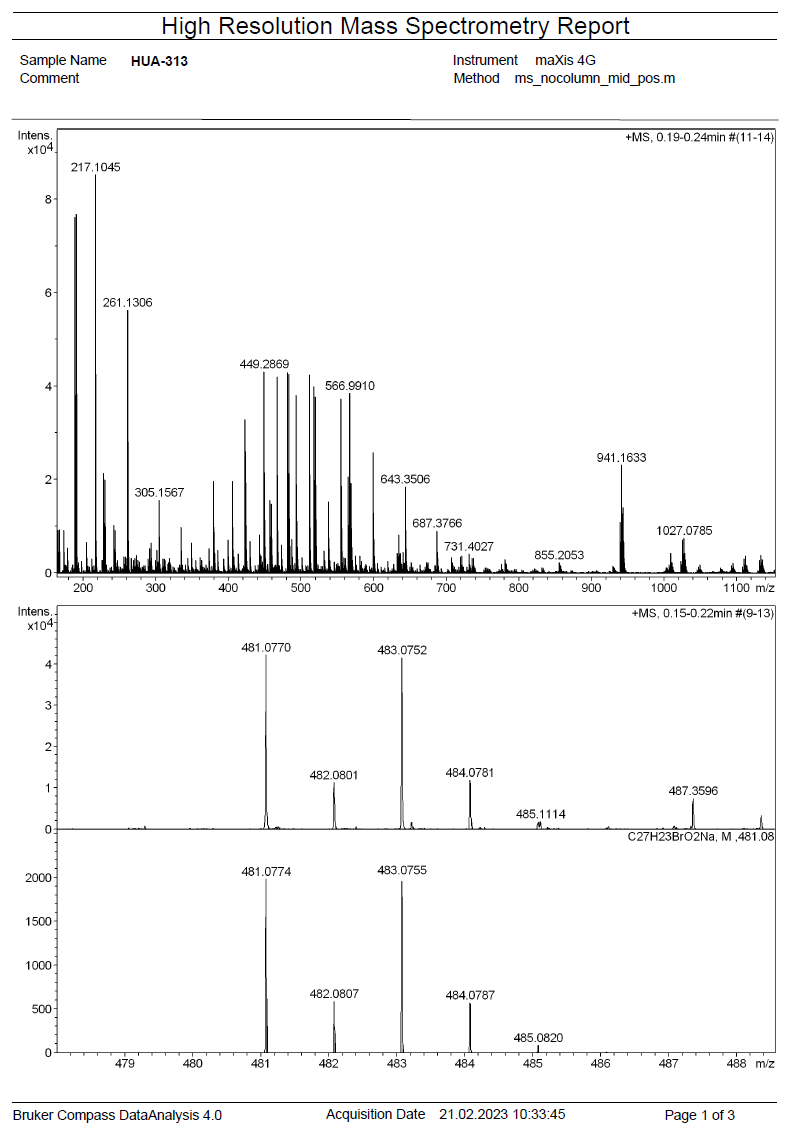


**Figure S30.** HRMS spectra of **4.**


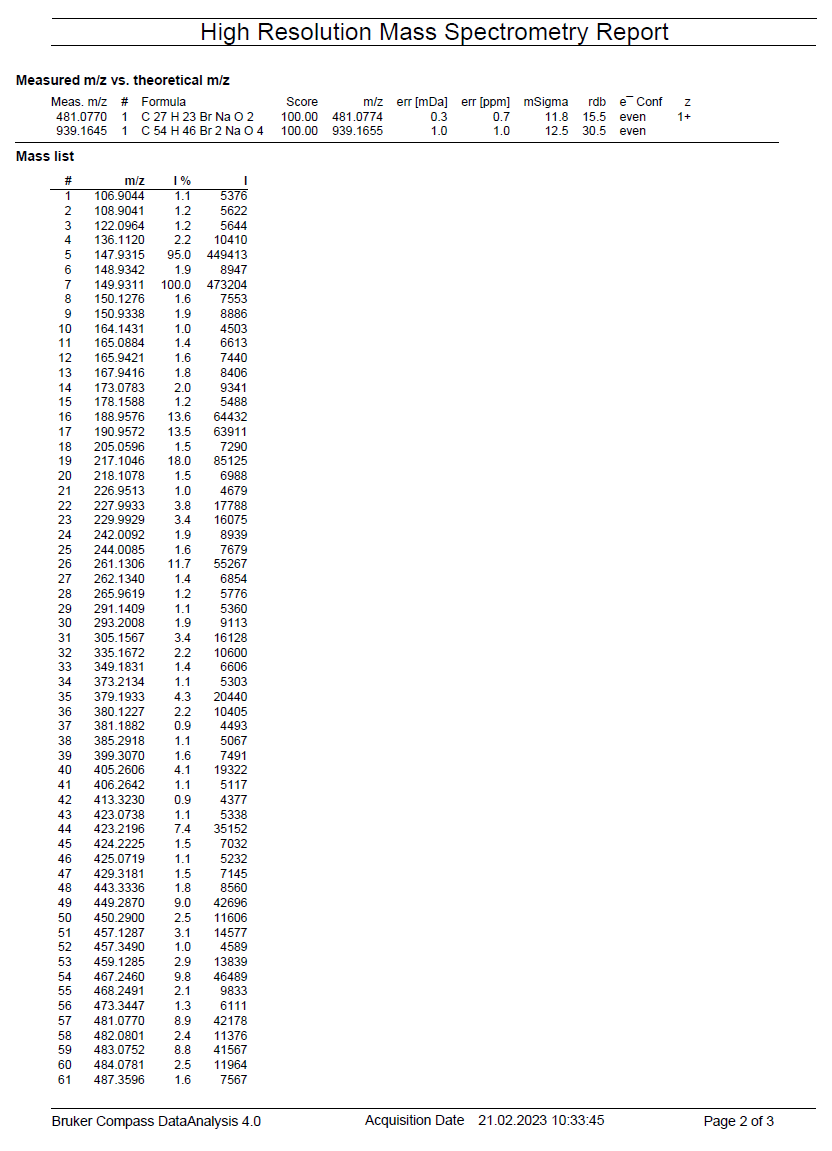


**Figure S31.** HRMS report of **4.**

#### 7.1.4 **S-5**


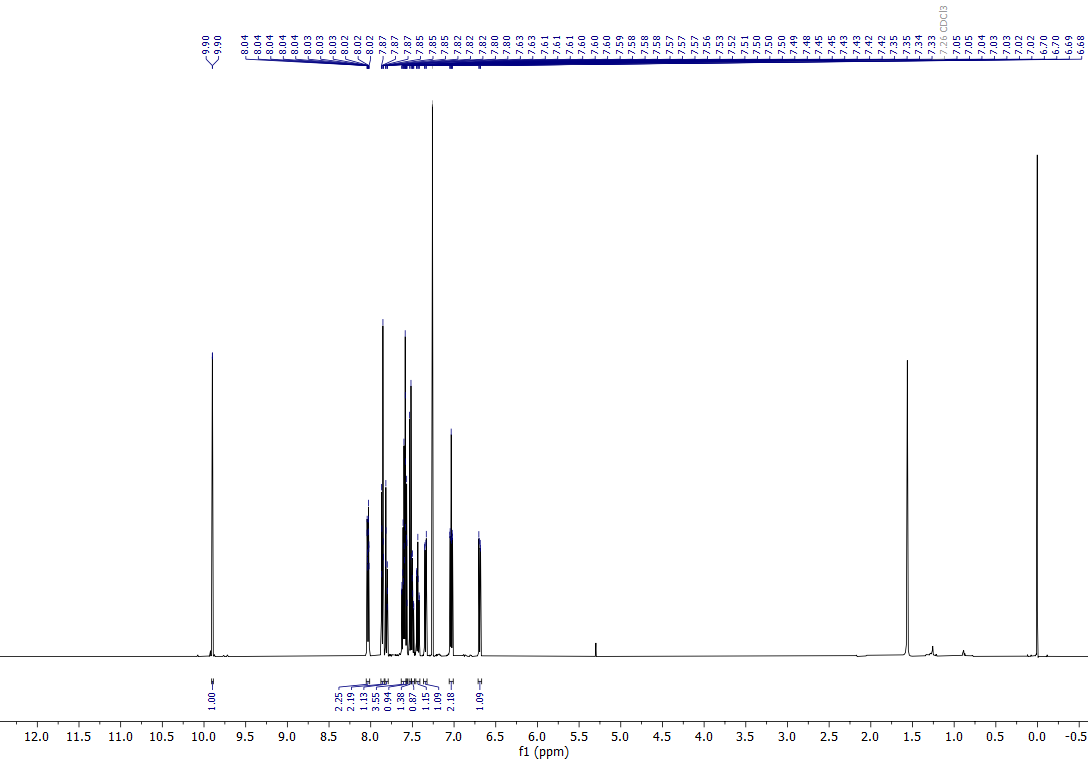


**Figure S32.** ^1^H NMR spectrum of **S-5** in CDCl_3_.


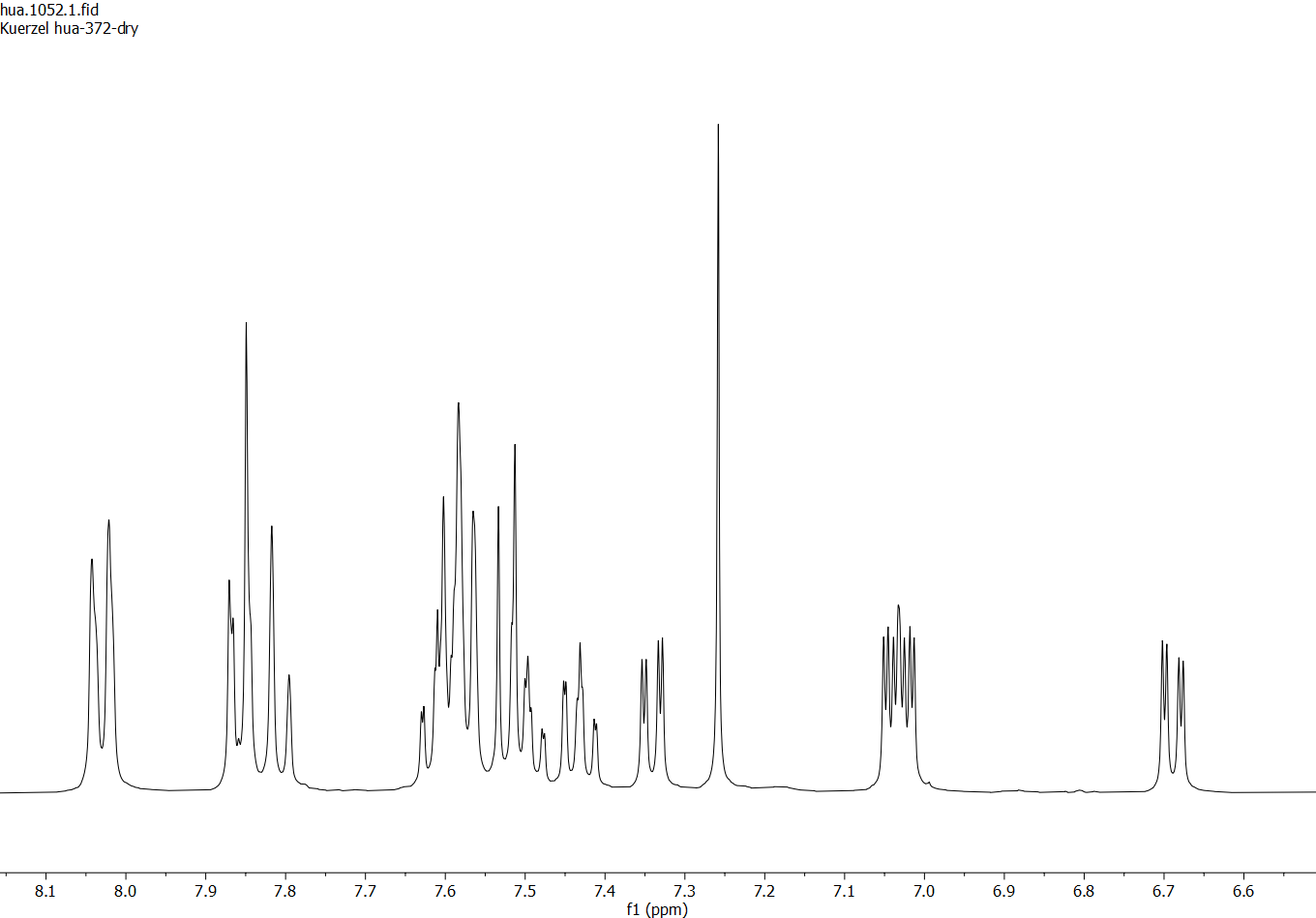


**Figure S33.** Aromatic region of the ^1^H NMR spectrum of **S-5** in CDCl_3_.


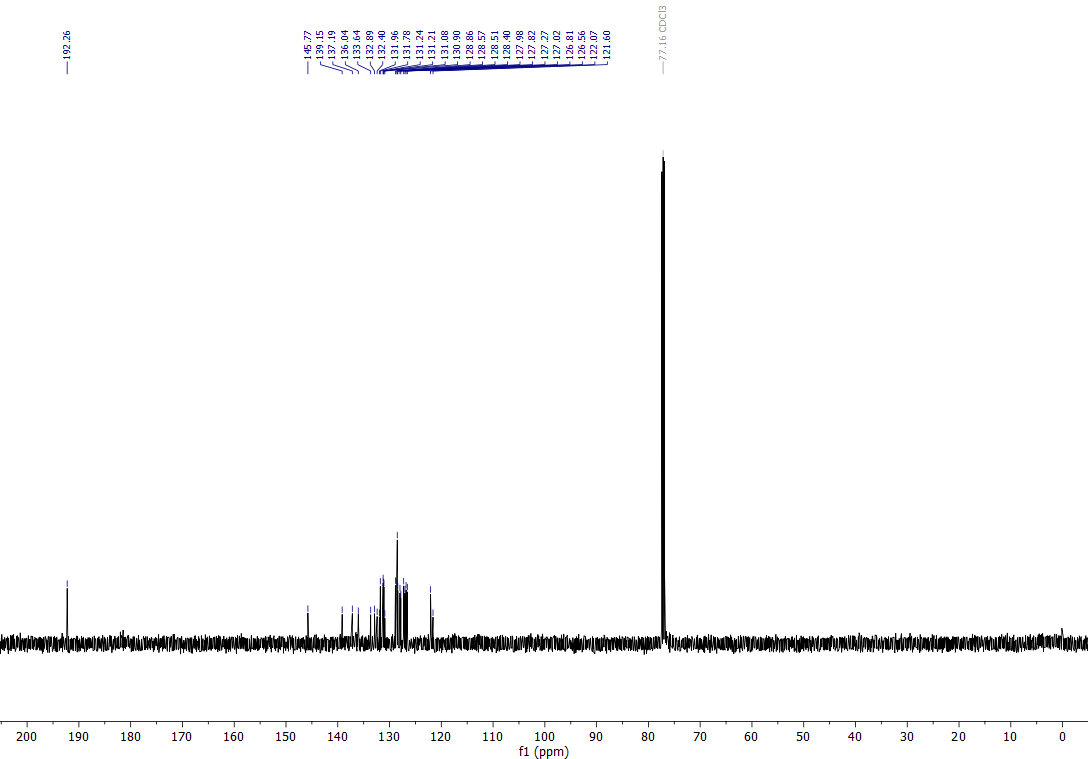


**Figure S34.** ^13^C NMR spectrum of **S-5** in CDCl_3_.


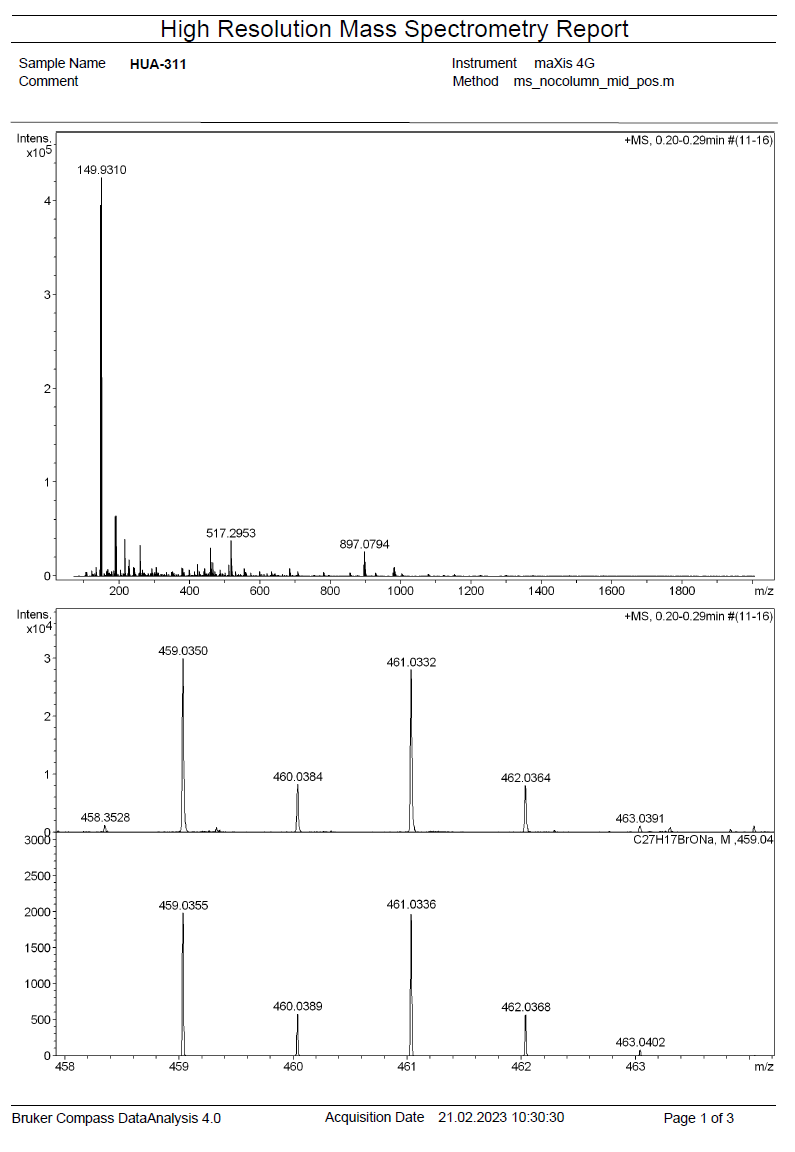


**Figure S35.** HRMS spectra of **S-5.**


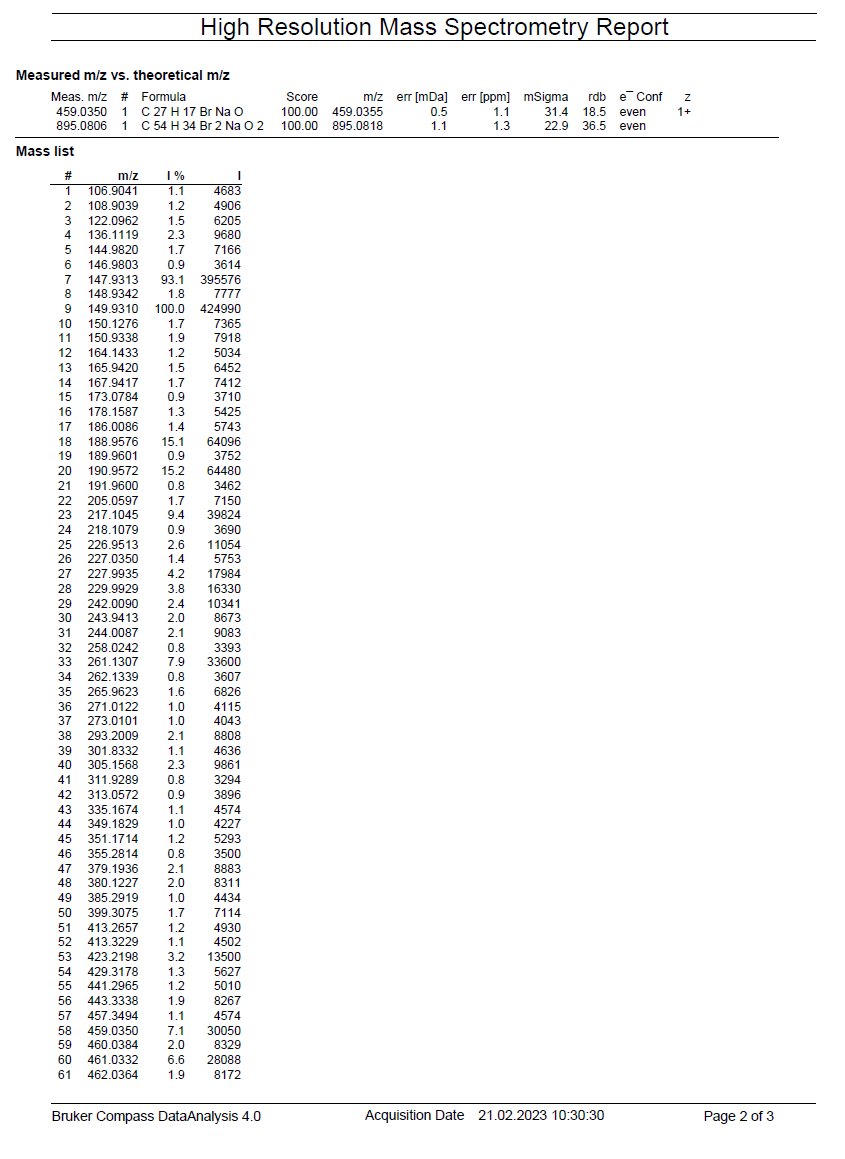


**Figure S36.** HRMS report of **S-5.**

#### 7.1.5 **10**

**
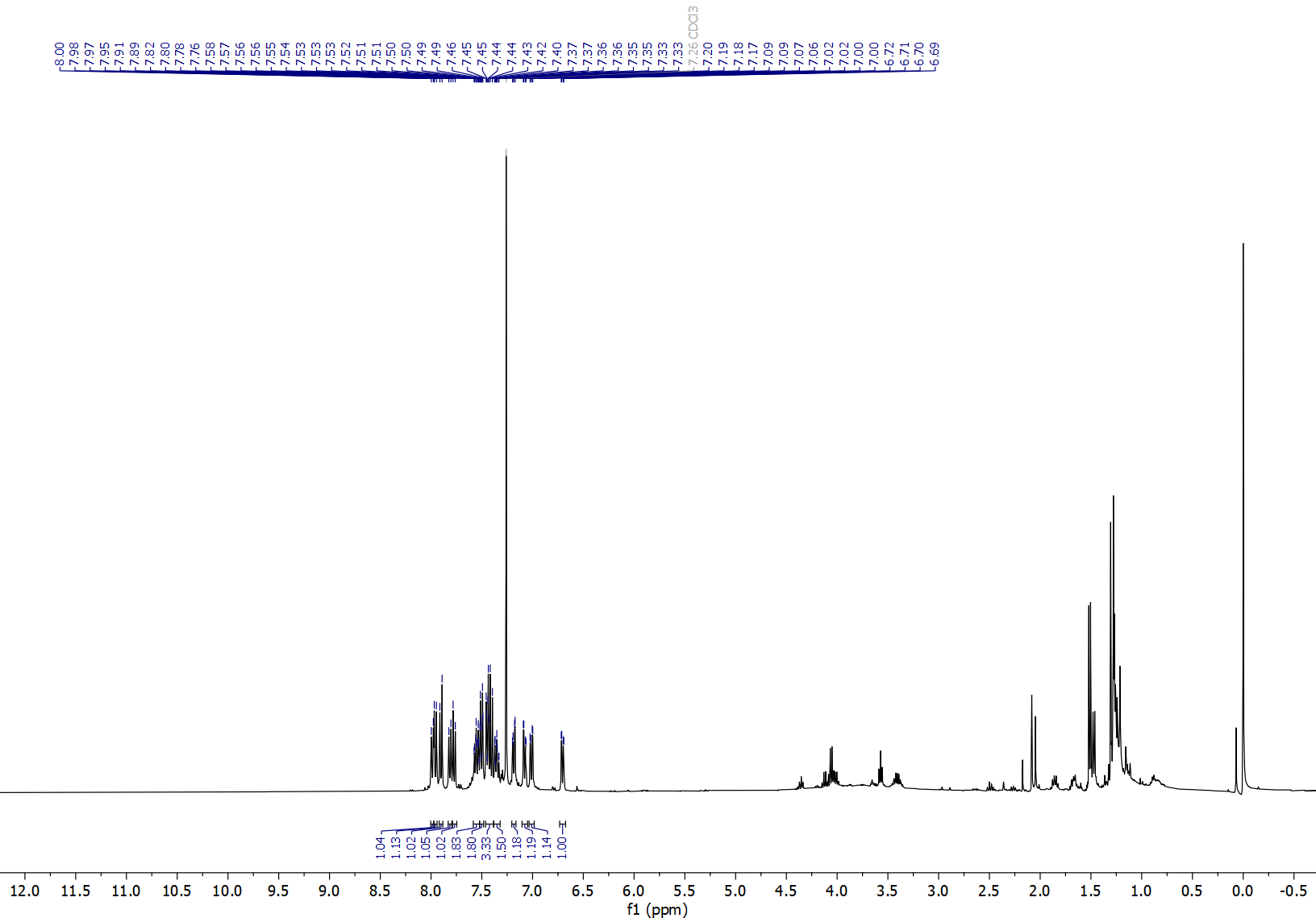
**

**Figure S37.** ^1^H NMR spectrum of **10** in CDCl_3_.


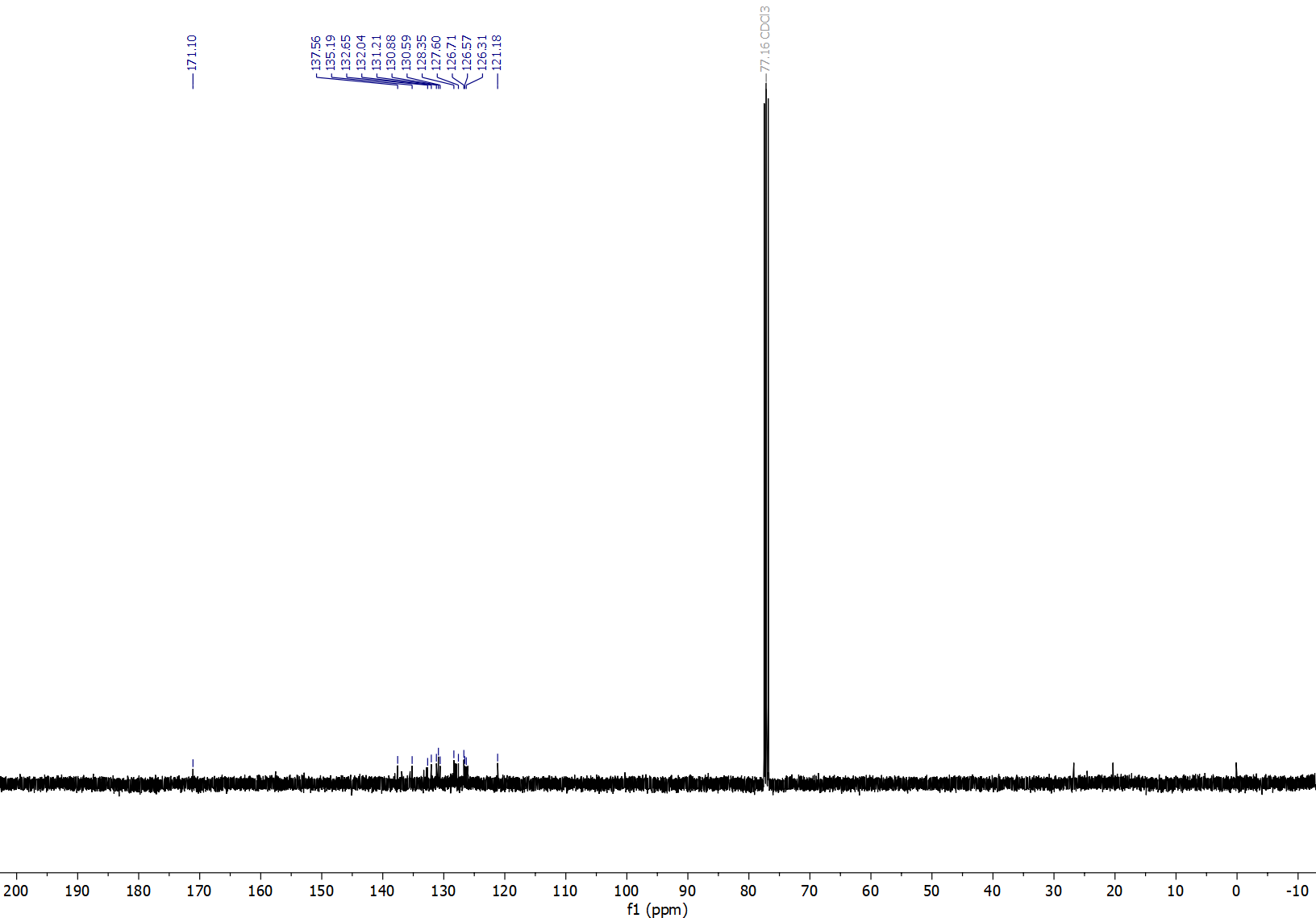


**Figure S38.** ^13^C NMR spectrum of **10** in CDCl_3_.

#### 7.1.6 **S-6**


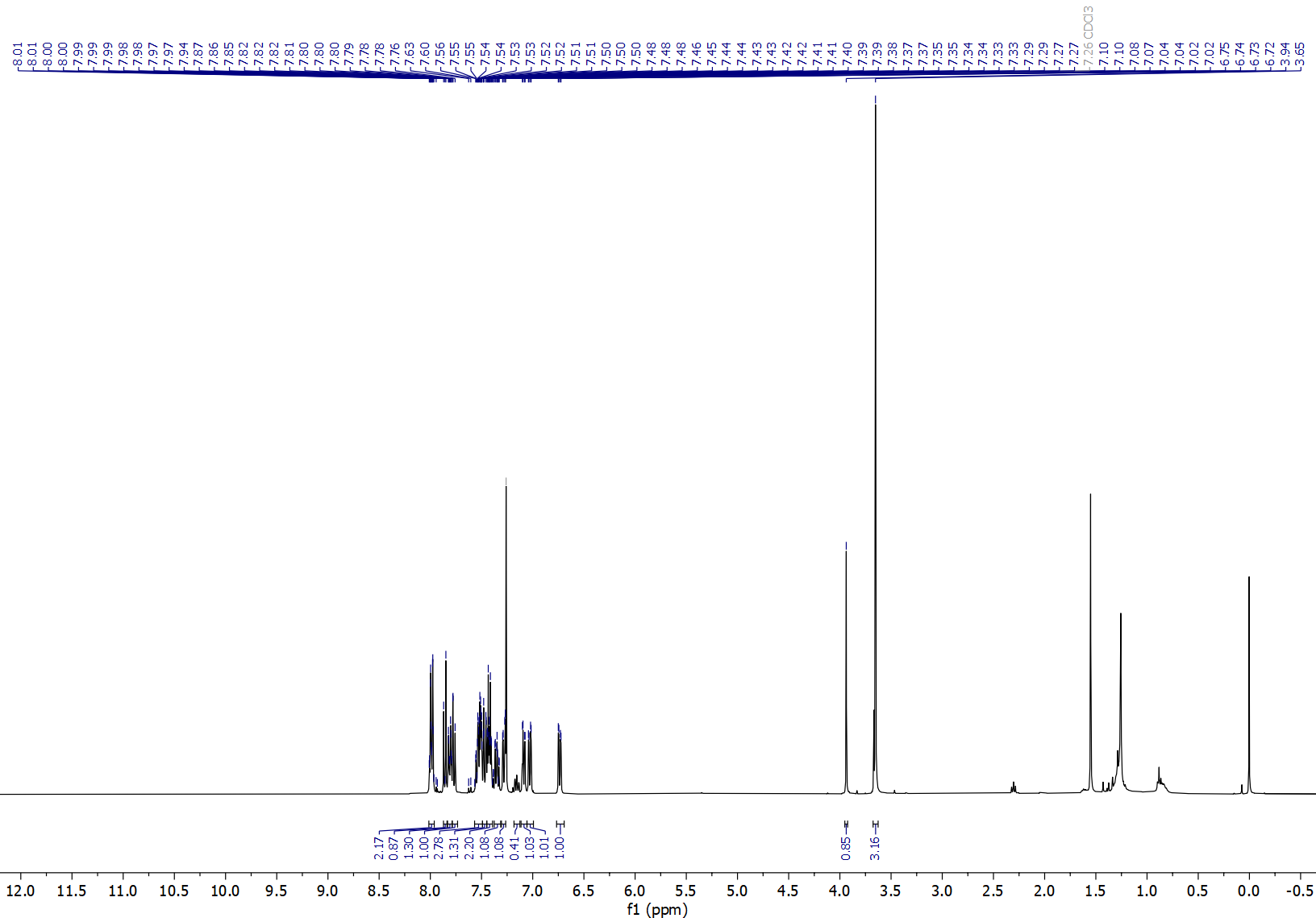


**Figure S39.** ^1^H NMR spectrum of **S-6** in CDCl_3_.


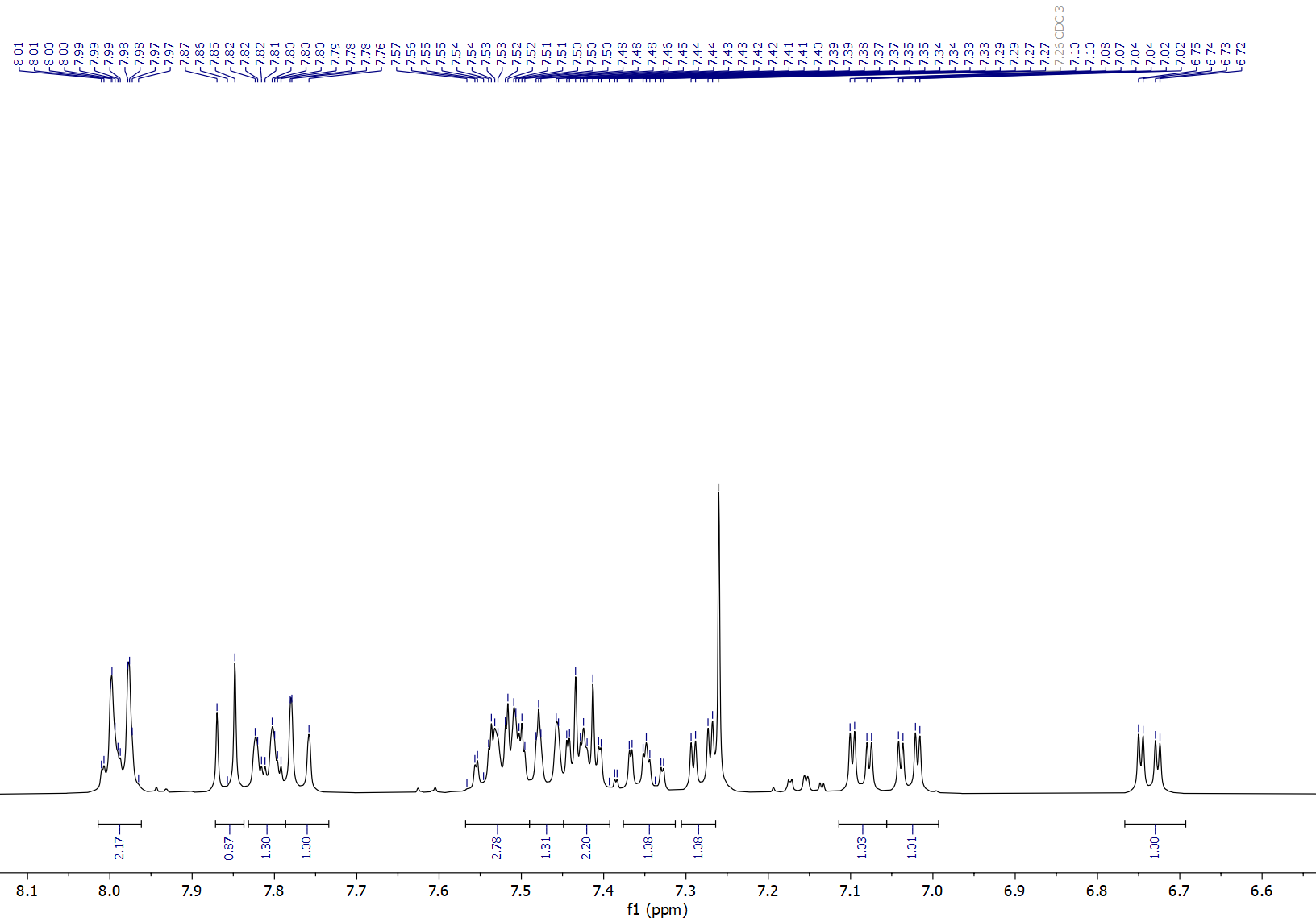


**Figure S40.** Aromatic region of the ^1^H NMR spectrum of **S-6** in CDCl_3_.


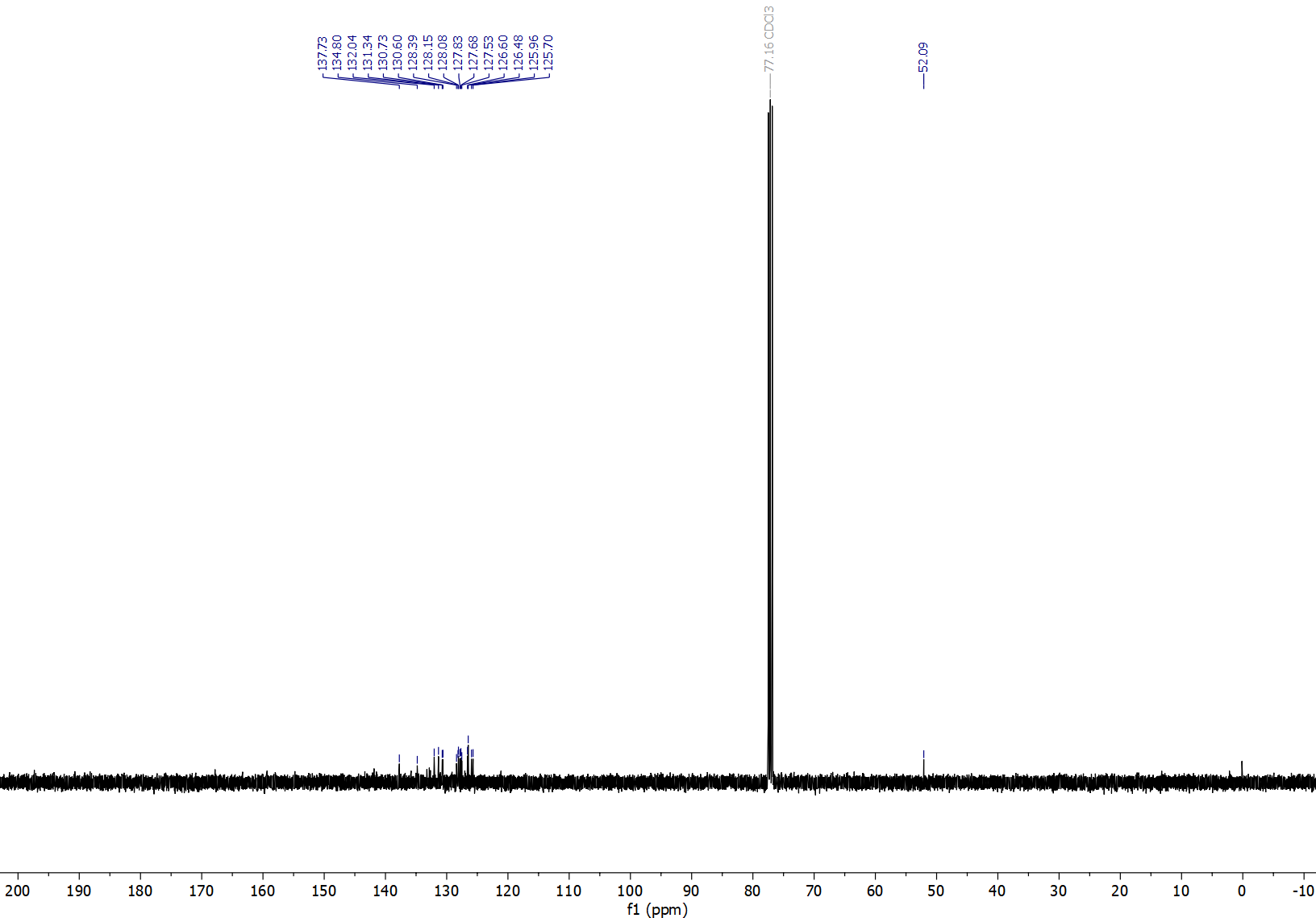


**Figure S41**. ^13^C NMR spectrum of **S-6** in CDCl_3_.


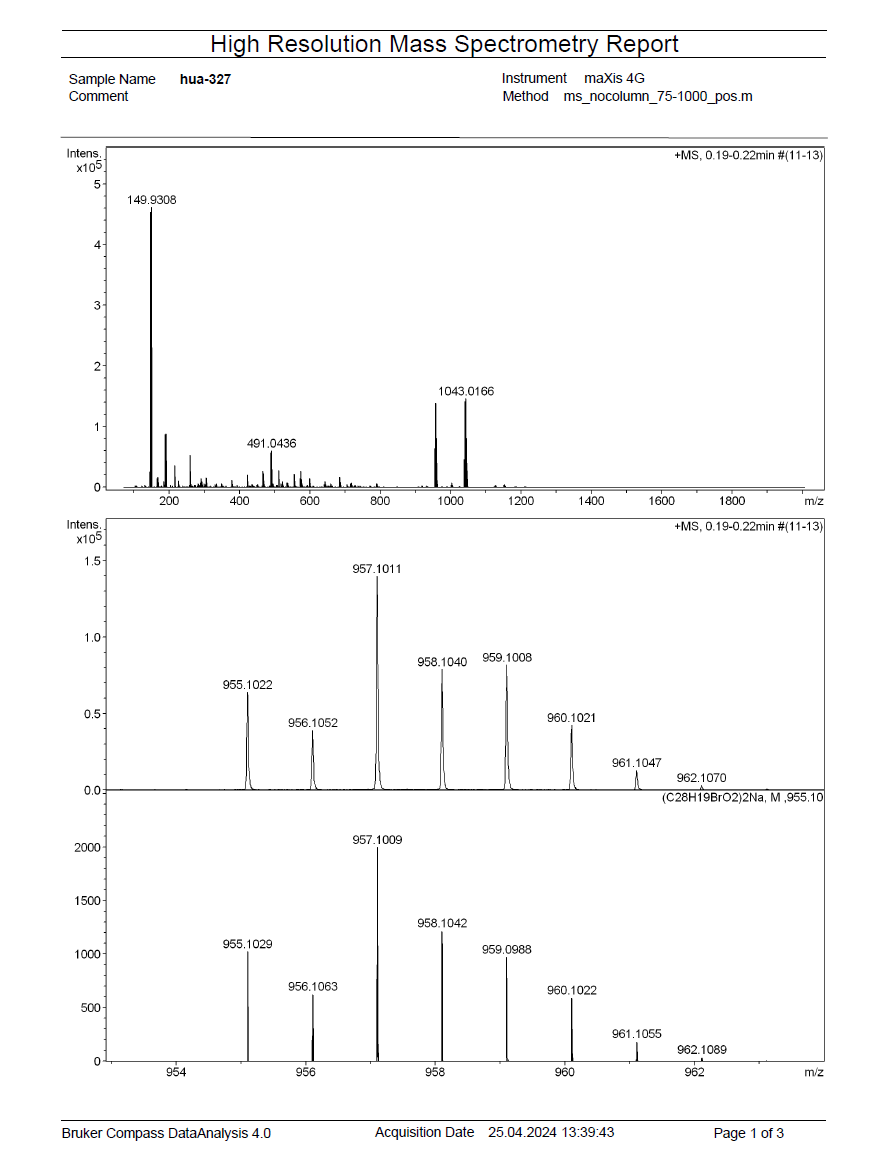


**Figure S42**. HRMS spectra of **S-6.**


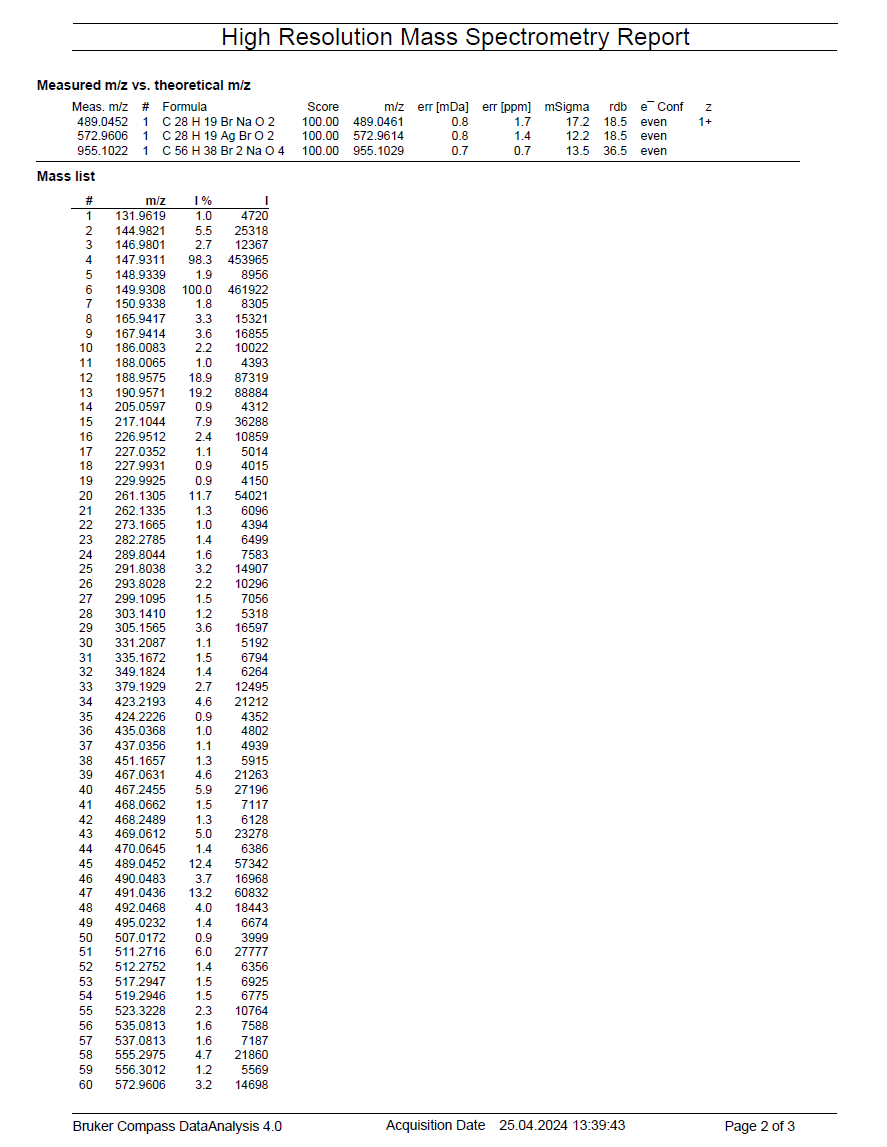


**Figure S43.** HRMS report of **S-6.**

#### 7.1.7 **11**


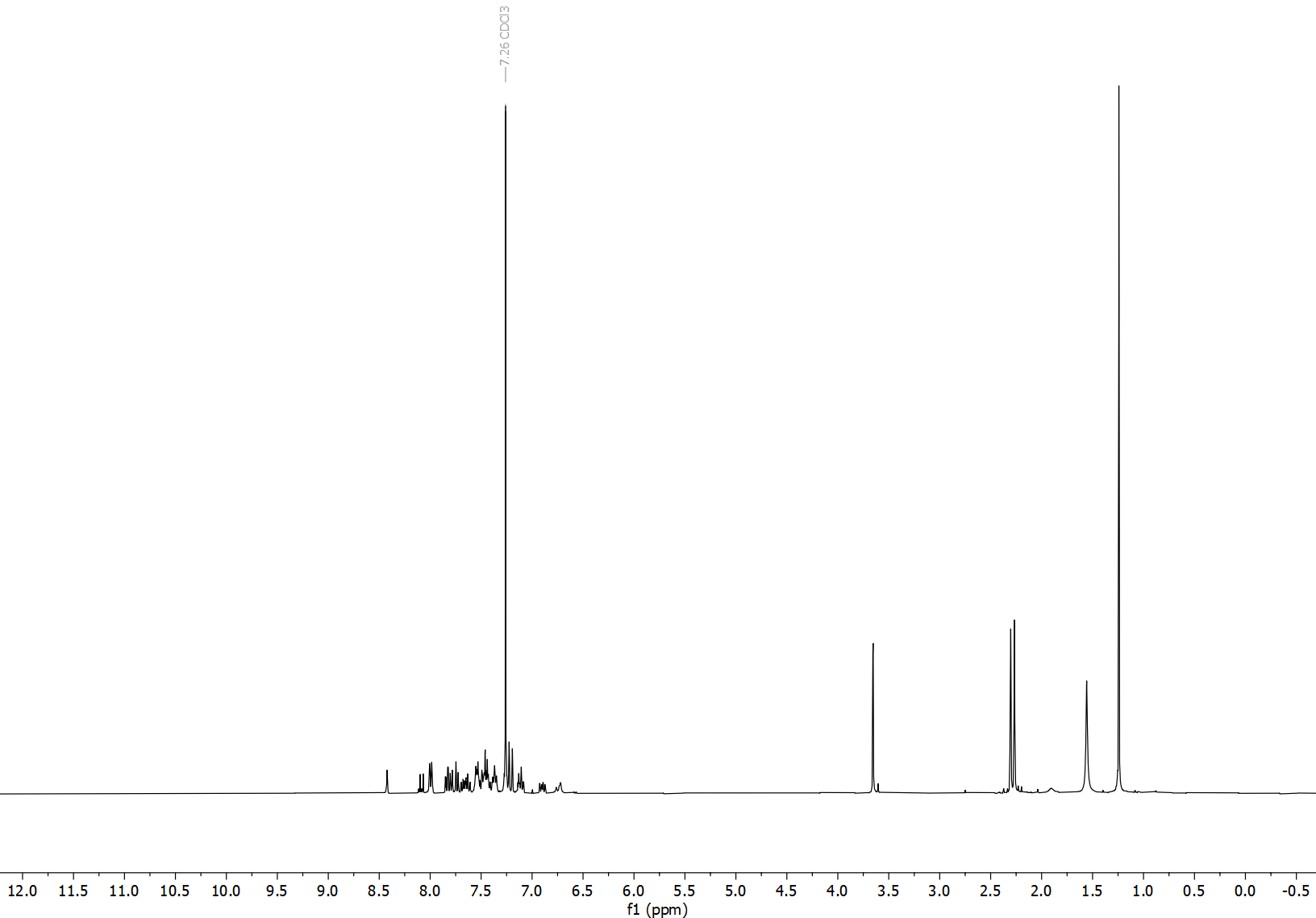


**Figure S44.** ^1^H NMR spectrum of **11** in CDCl_3_.


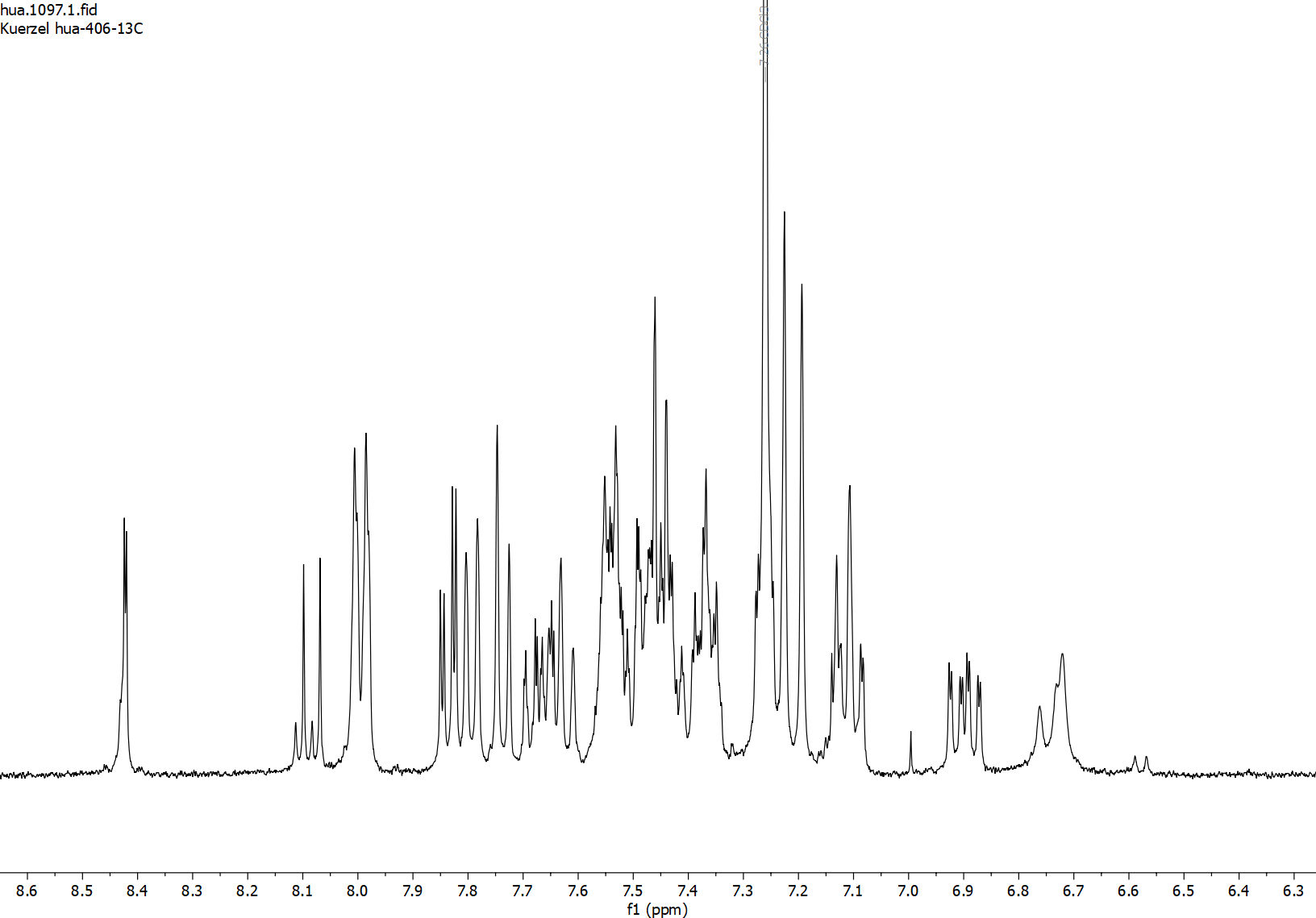


**Figure S45.** Aromatic region of the ^1^H NMR spectrum of **11** in CDCl_3_.


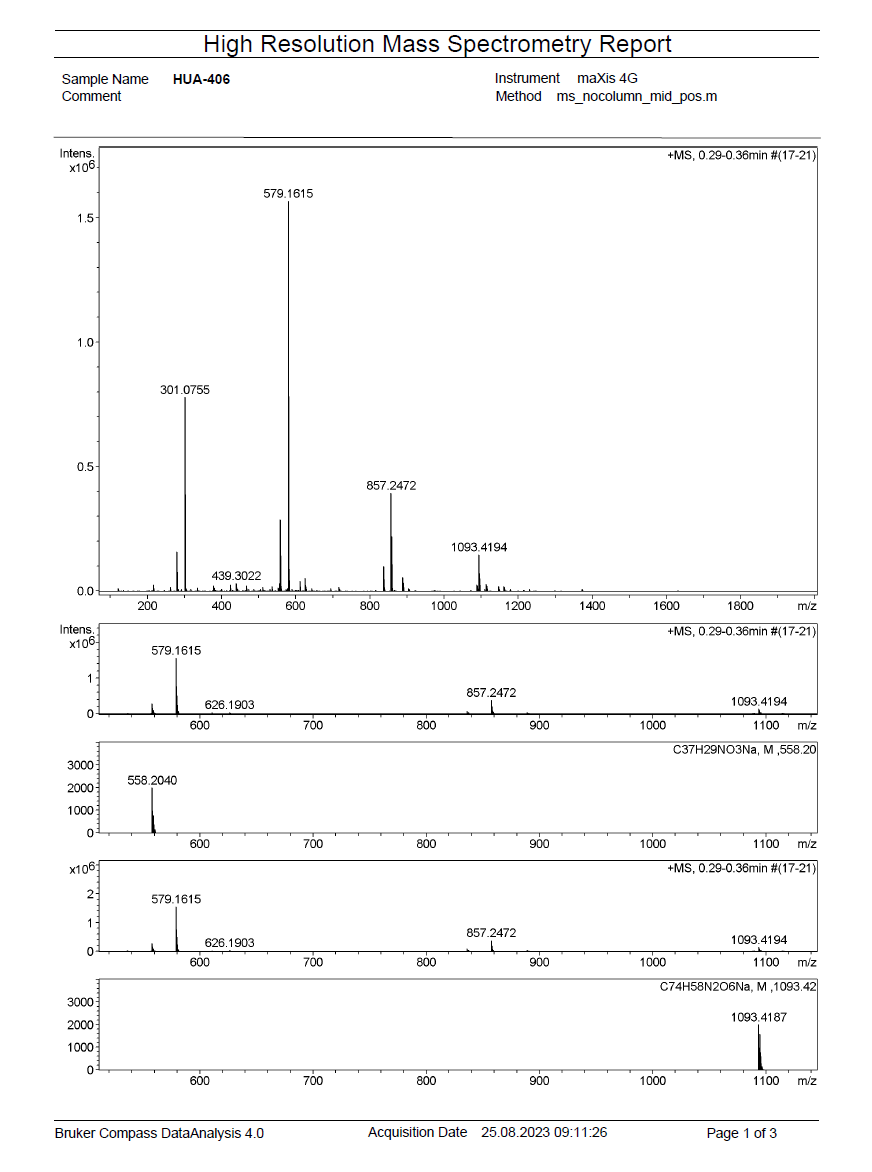


**Figure S46.** HRMS spectra of **11.**


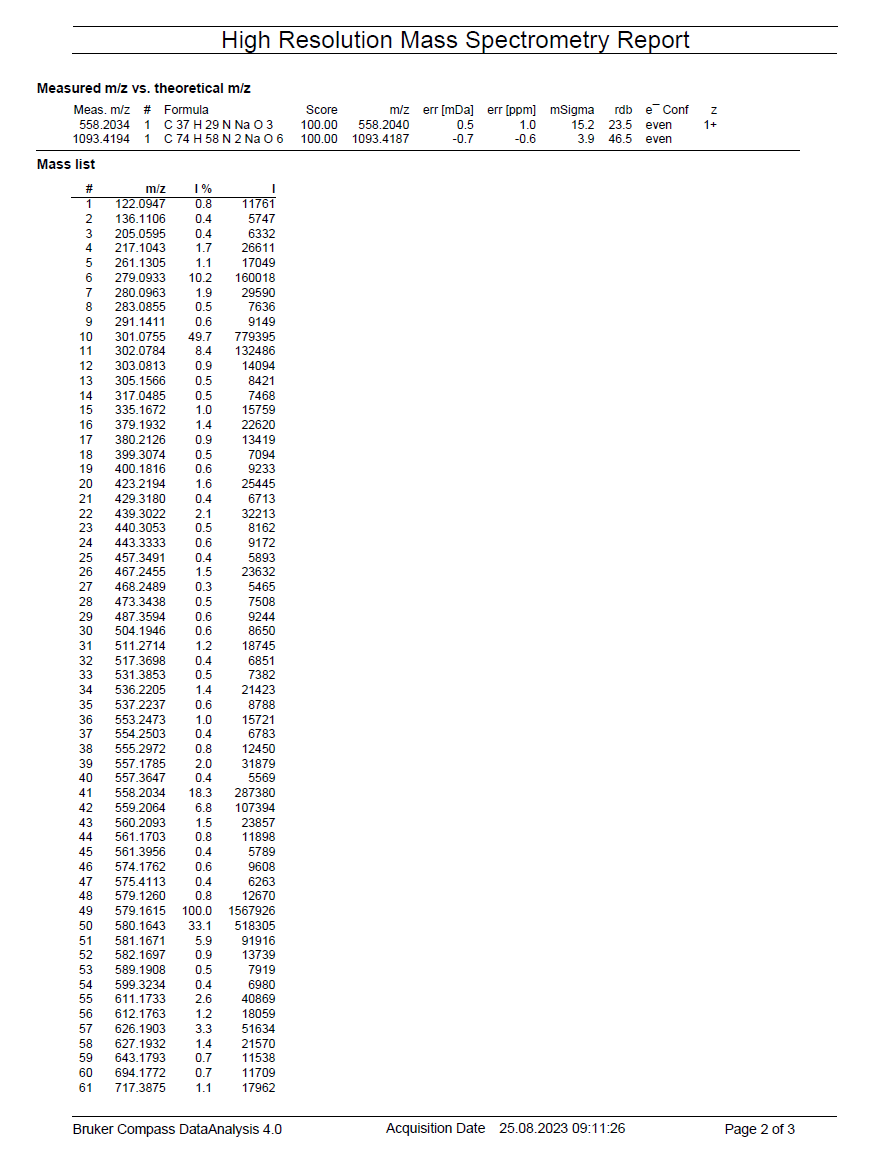


**Figure S47.** HRMS report of **11.**

#### 7.1.8 **S-8**


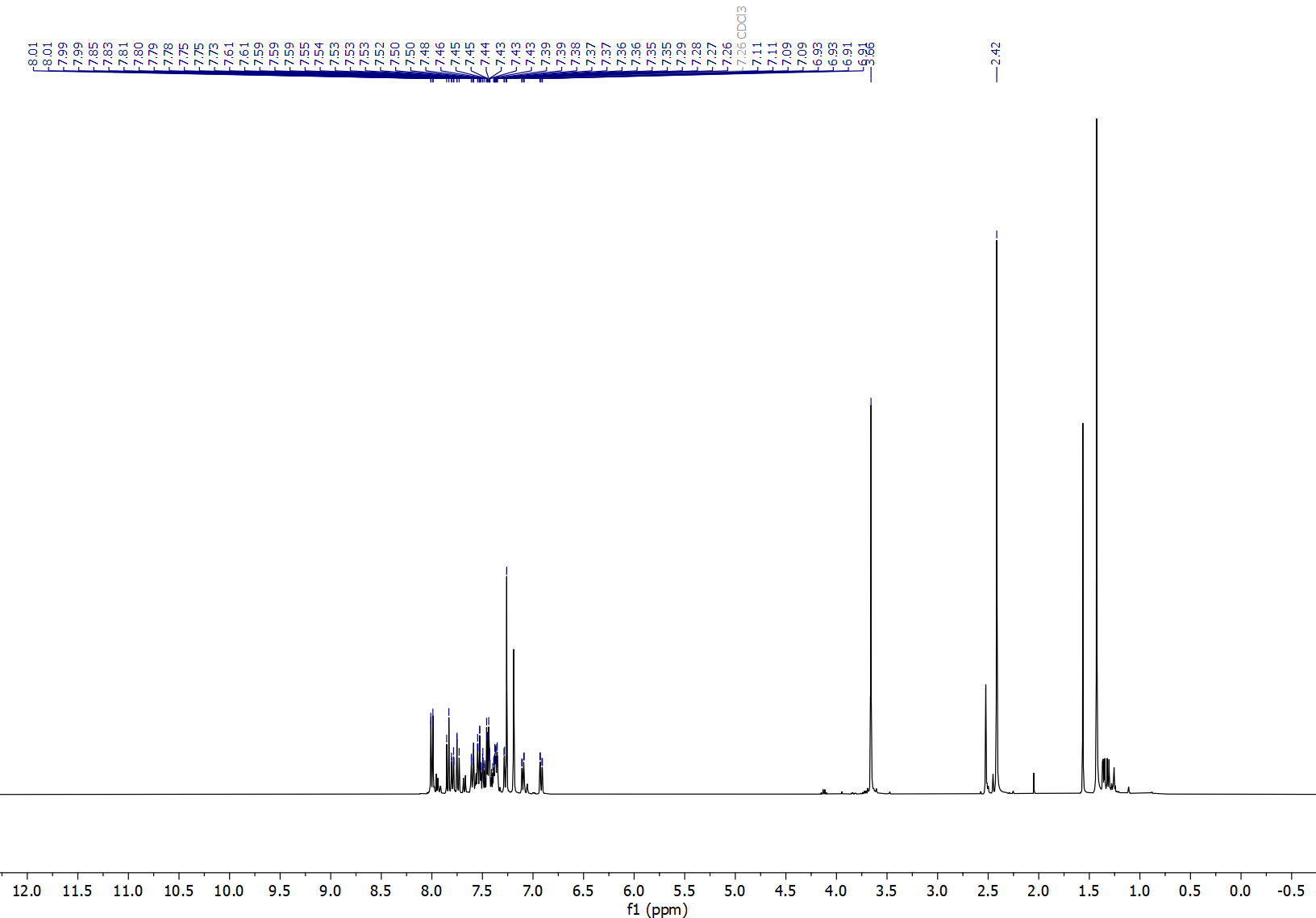


**Figure S48.** ^1^H NMR spectrum of **S-8** in CDCl_3_.


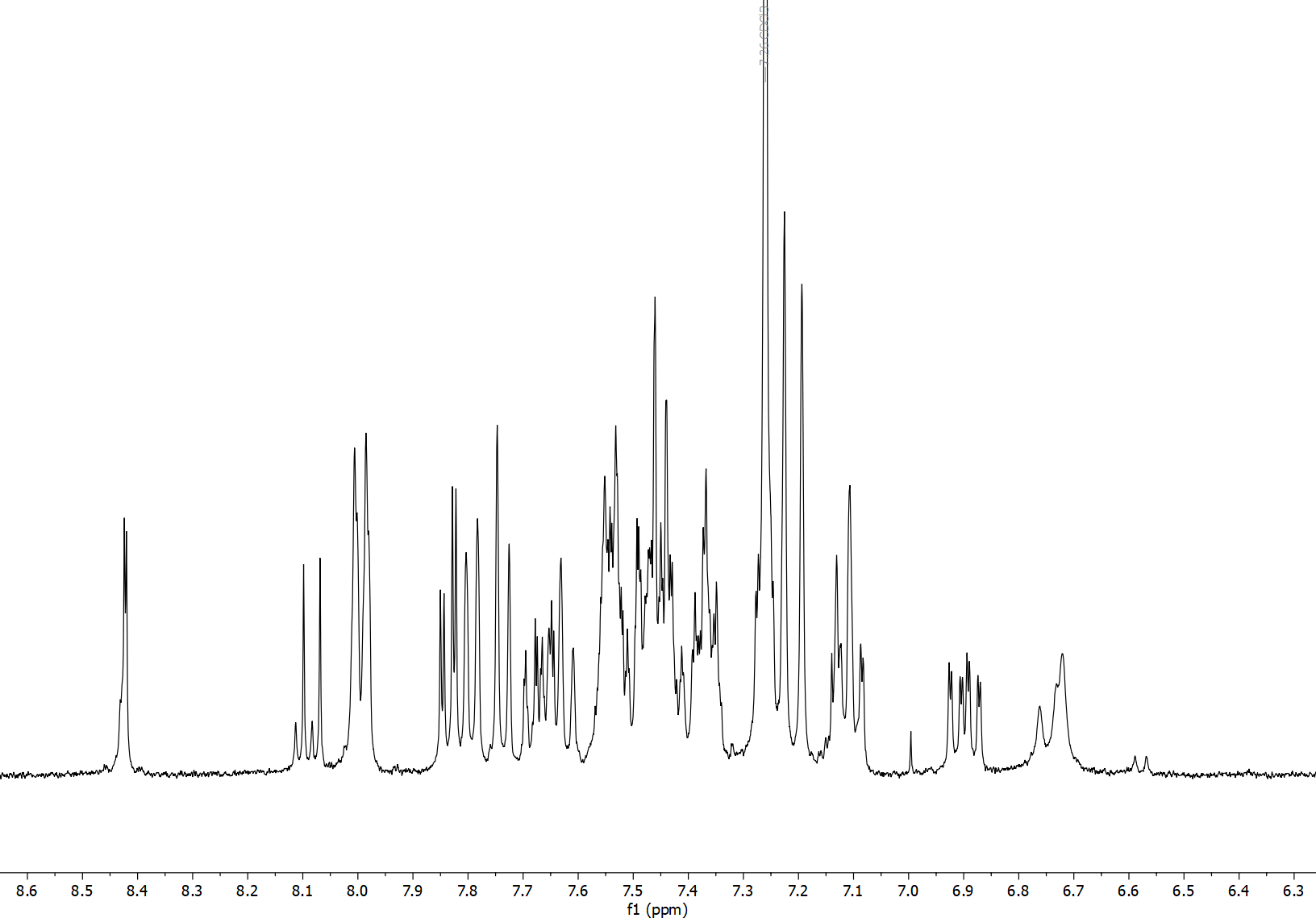


**Figure S49.** Aromatic region of the ^1^H NMR spectrum of **S-8** in CDCl_3_.


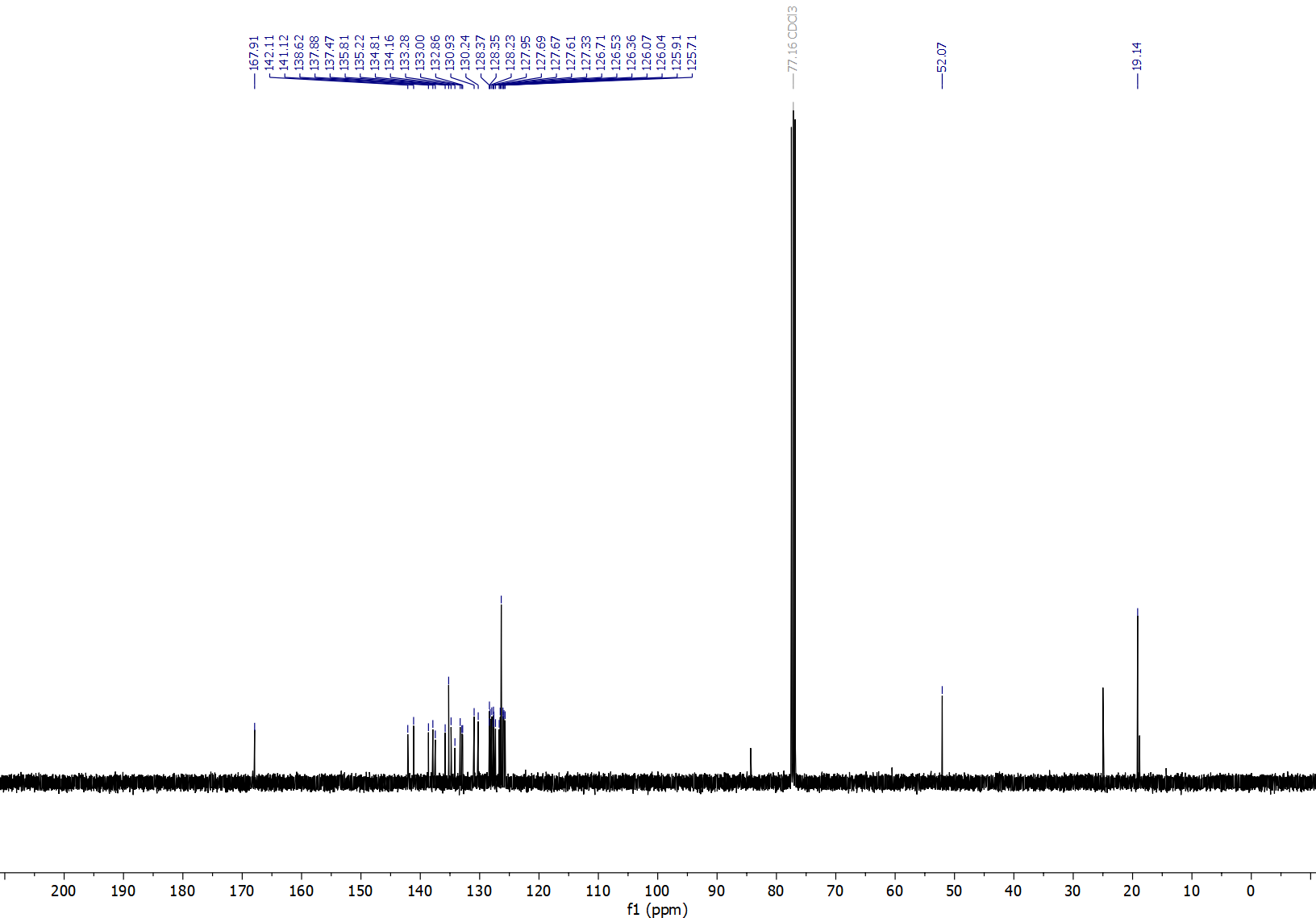


**Figure S50.** ^13^C NMR spectrum of **S-8** in CDCl_3_.


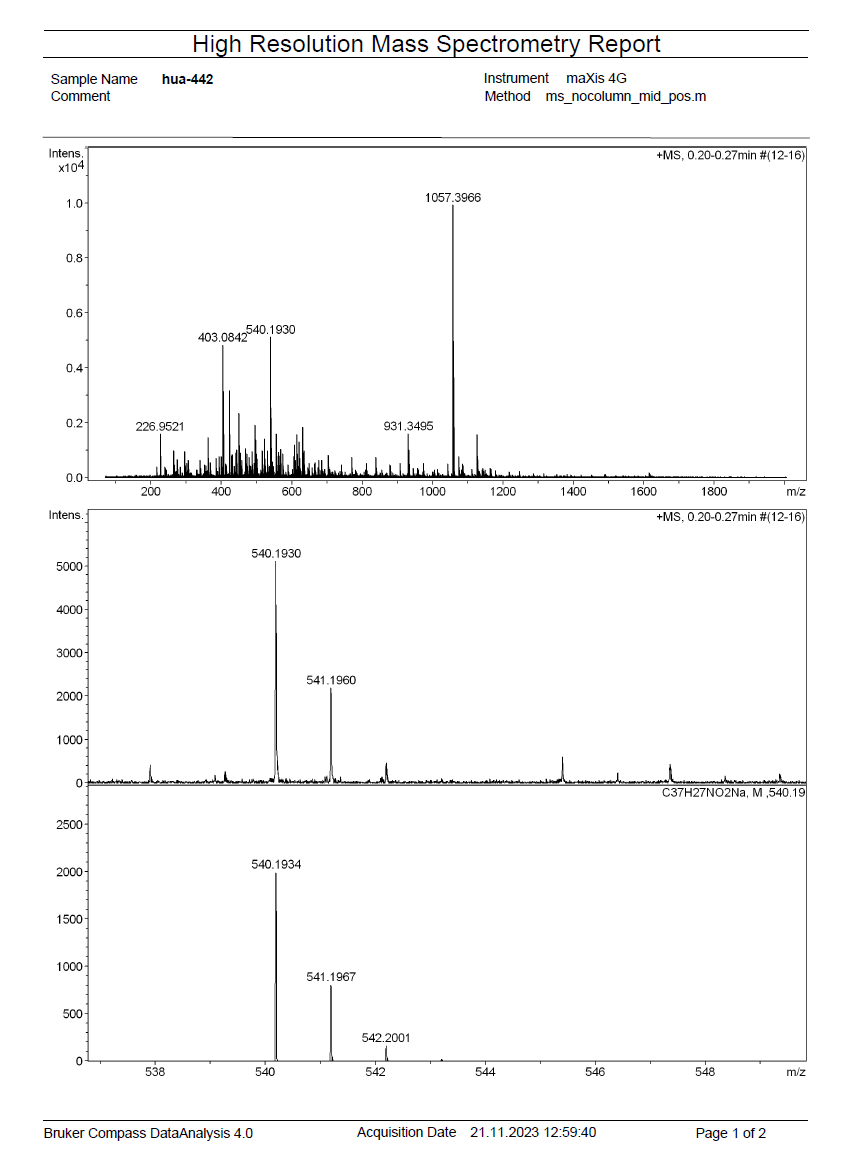


**Figure S51.** HRMS spectra of **S-8.**


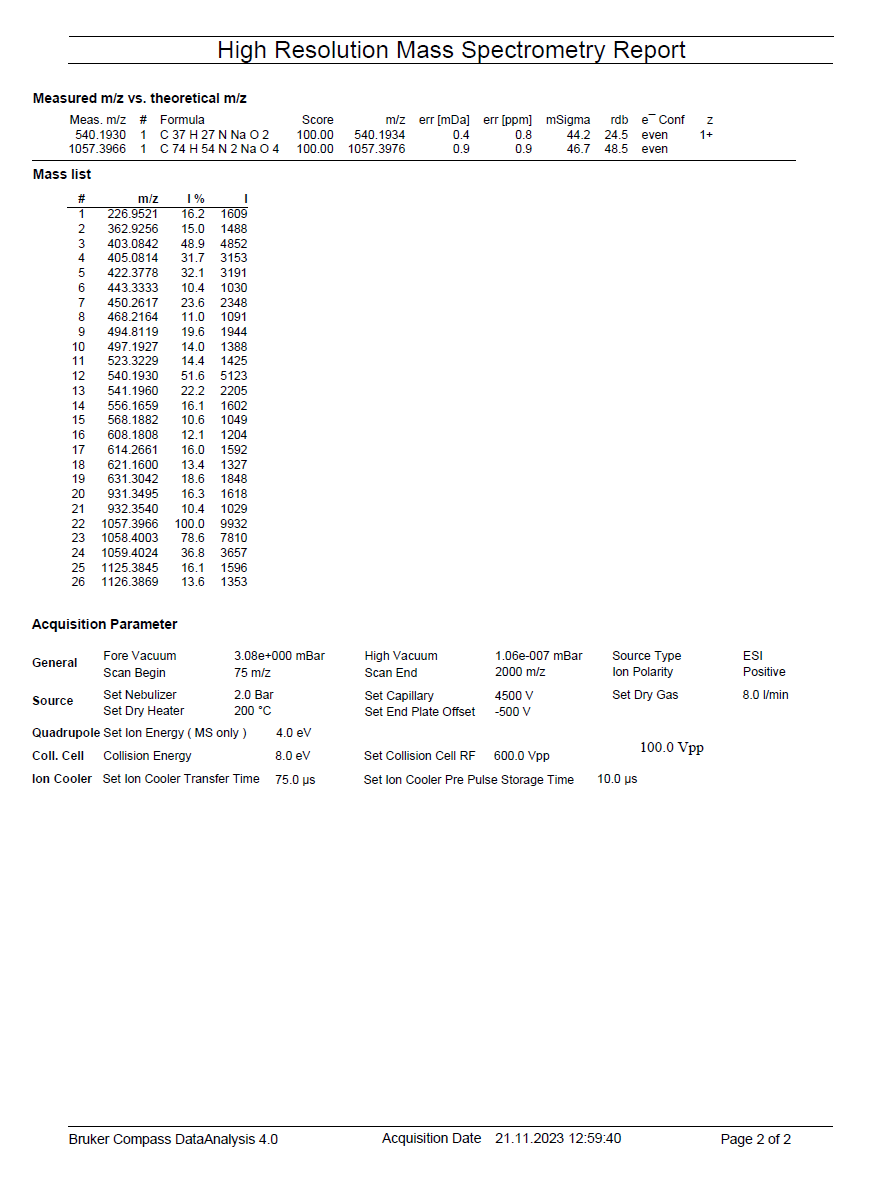


**Figure S52.** HRMS report of **S-8.**

### 7.2 Pt(II) complexes **9** and ***S*-1**

#### 7.2.1 **9** (reference complex)


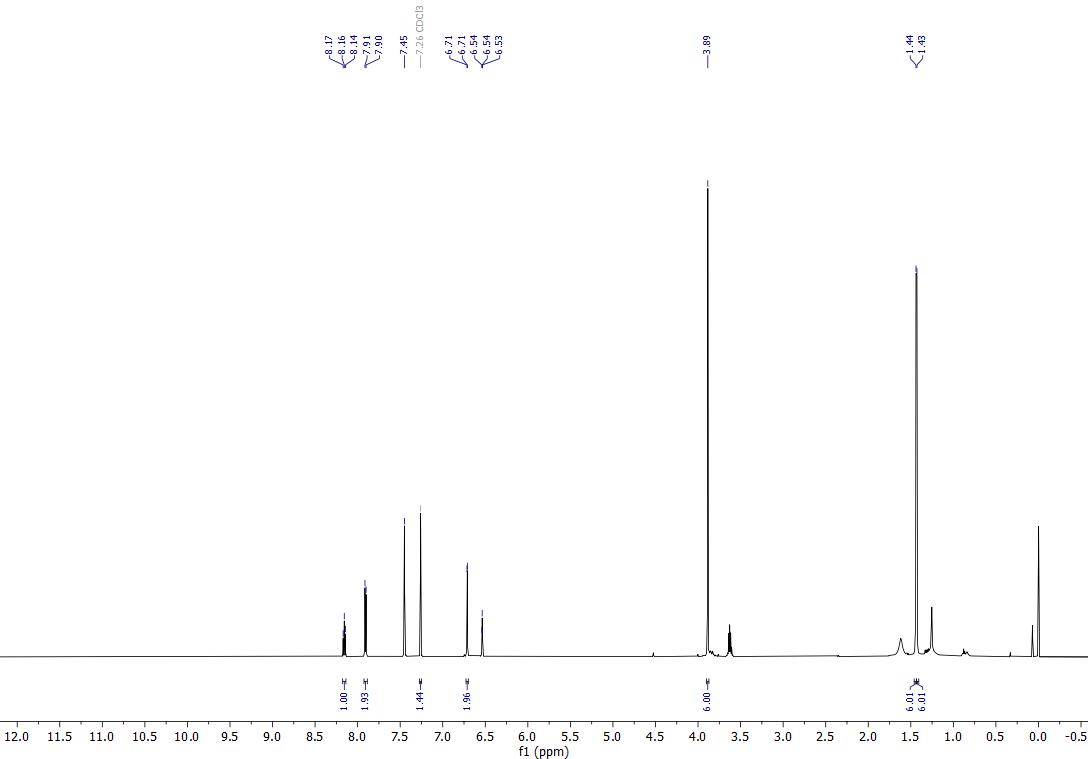


**Figure S53.** ^1^H NMR spectrum of **9** in CDCl_3_.


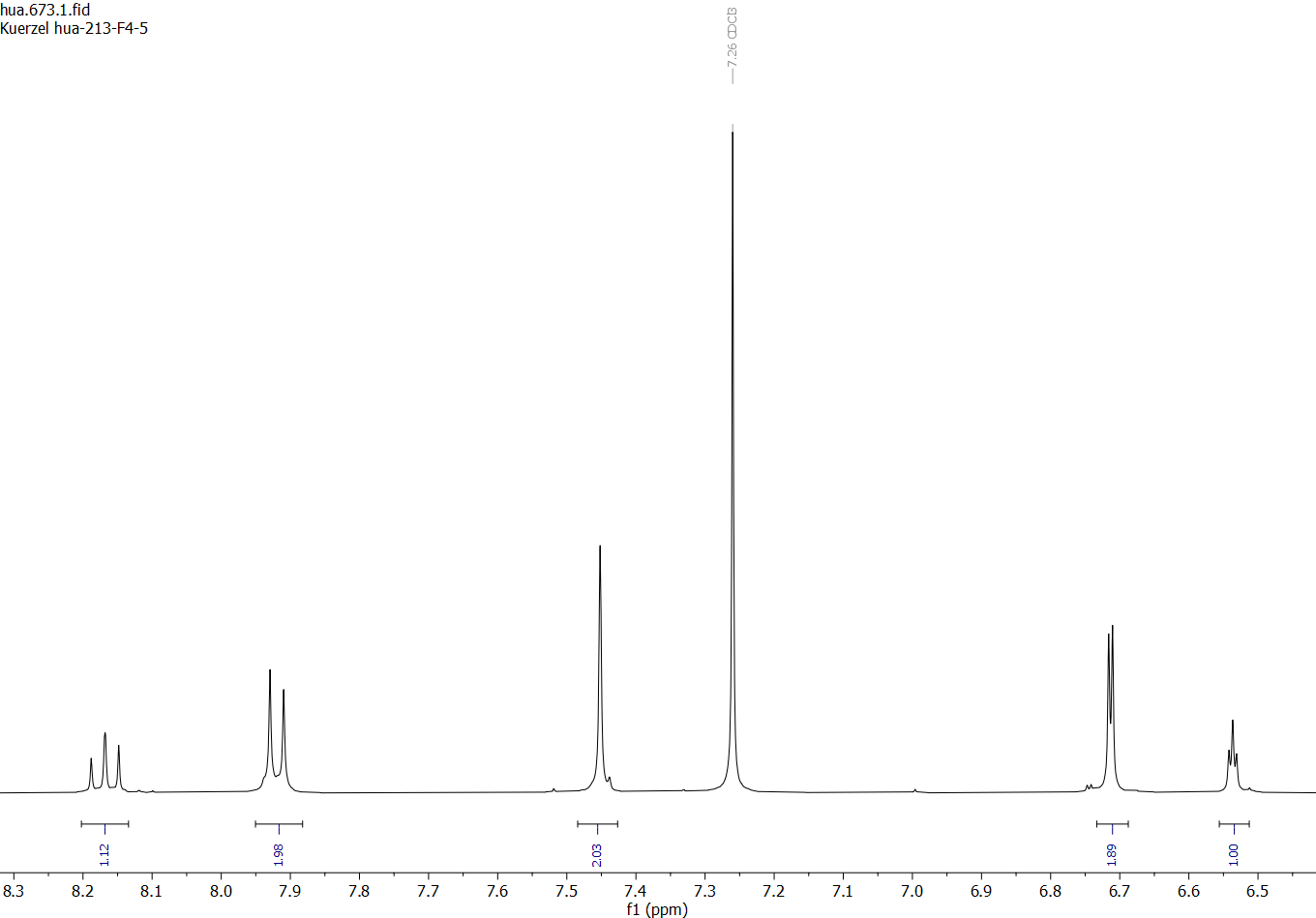


**Figure 54.** Aromatic region of the ^1^H NMR spectrum of **9** in CDCl_3_.


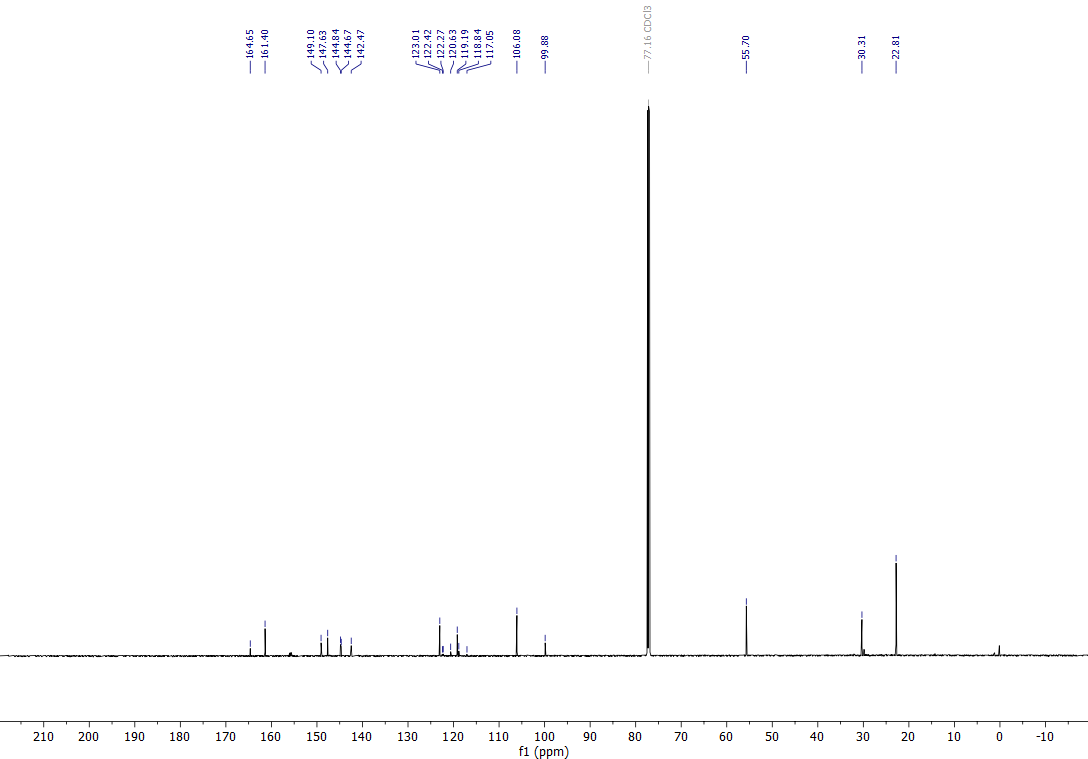


**Figure S55.** ^13^C NMR spectrum of **9** in CDCl_3_.


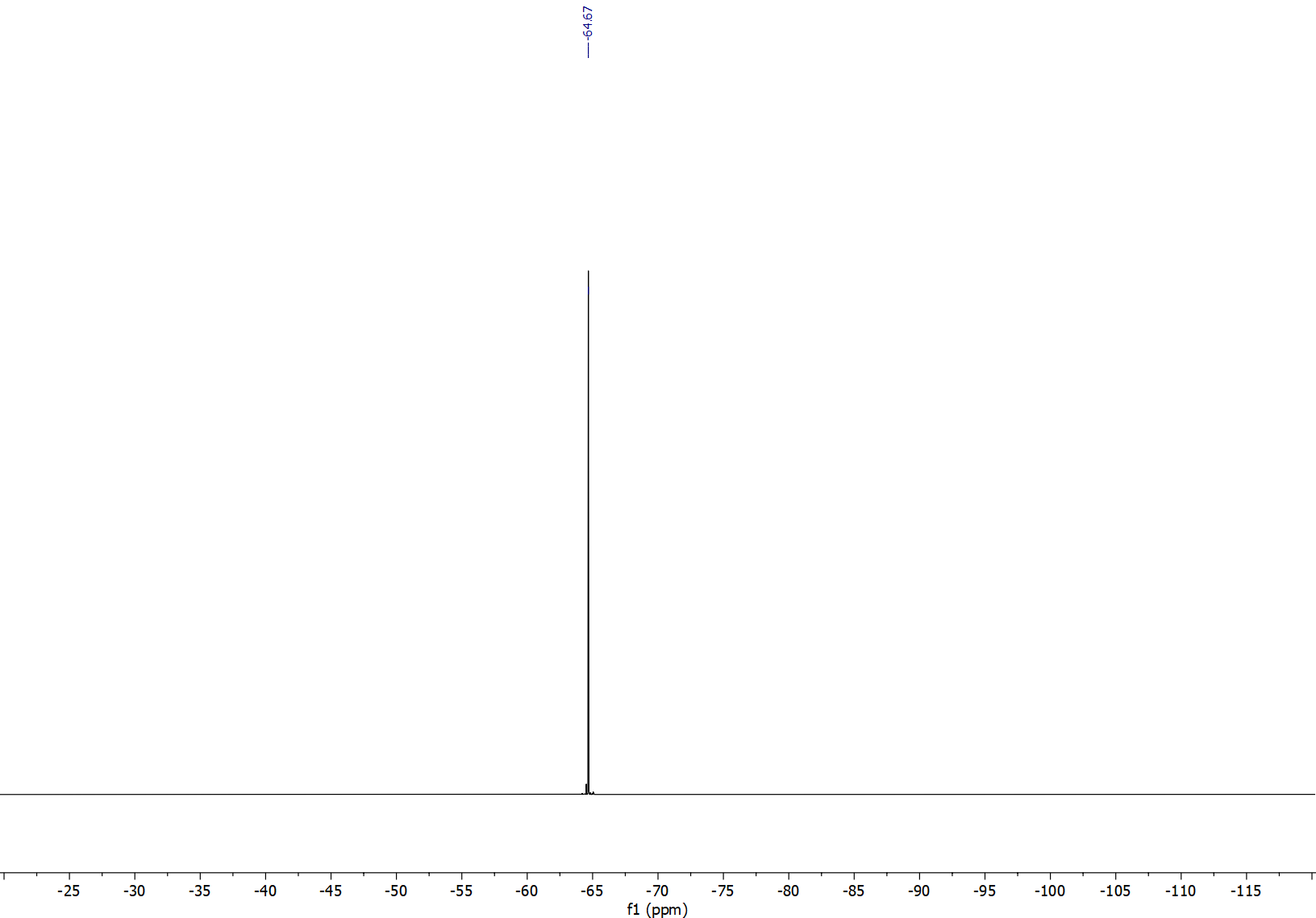


**Figure S56.** ^19^F NMR spectrum of **9** in CDCl_3_.


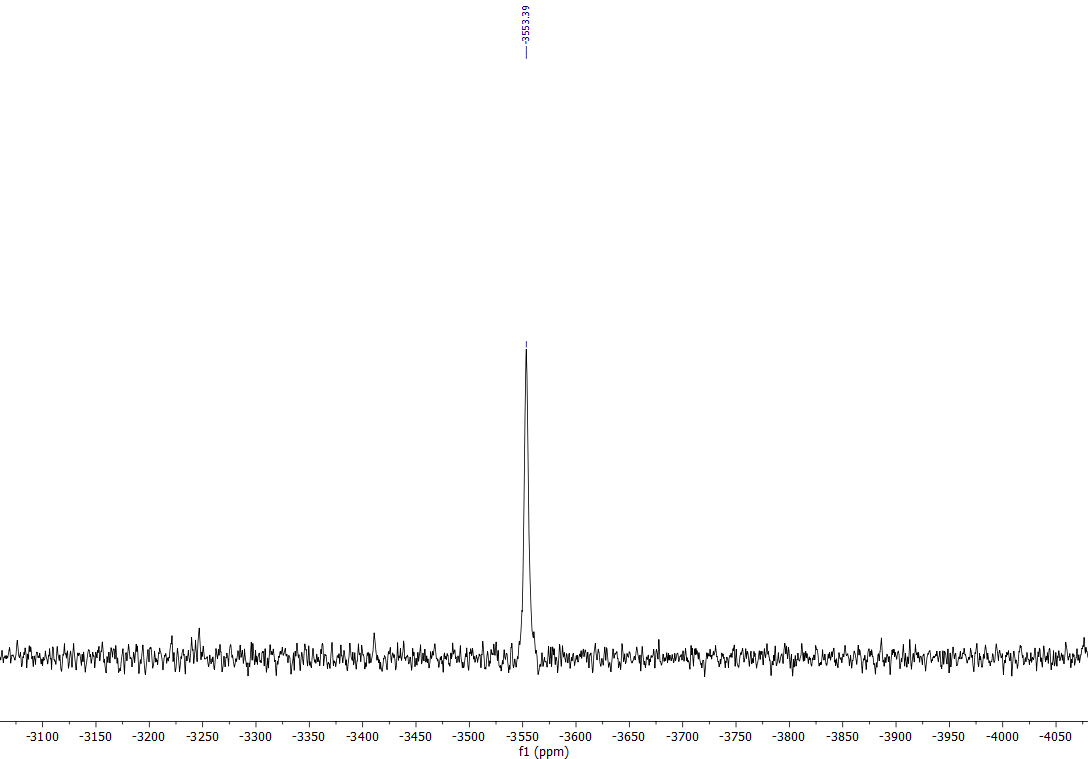


**Figure S57.** ^195^Pt NMR spectrum of **9** in CDCl_3_.


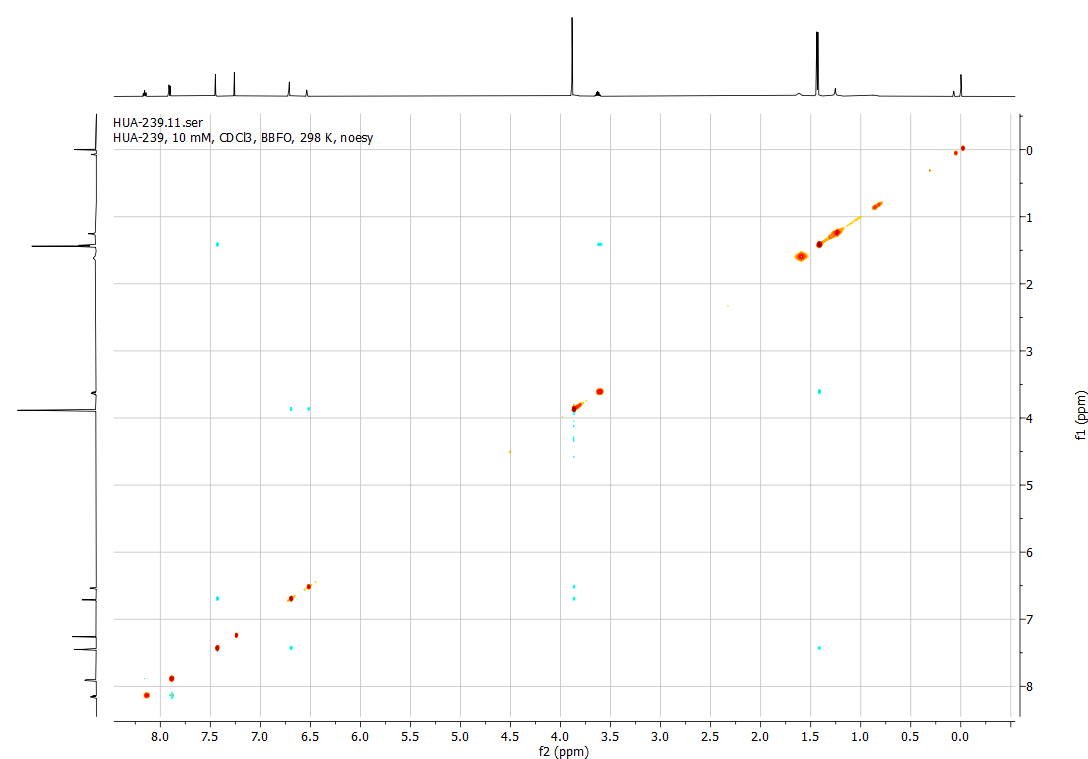


**Figure S58.** NOESY spectrum of **9** in CDCl_3_.


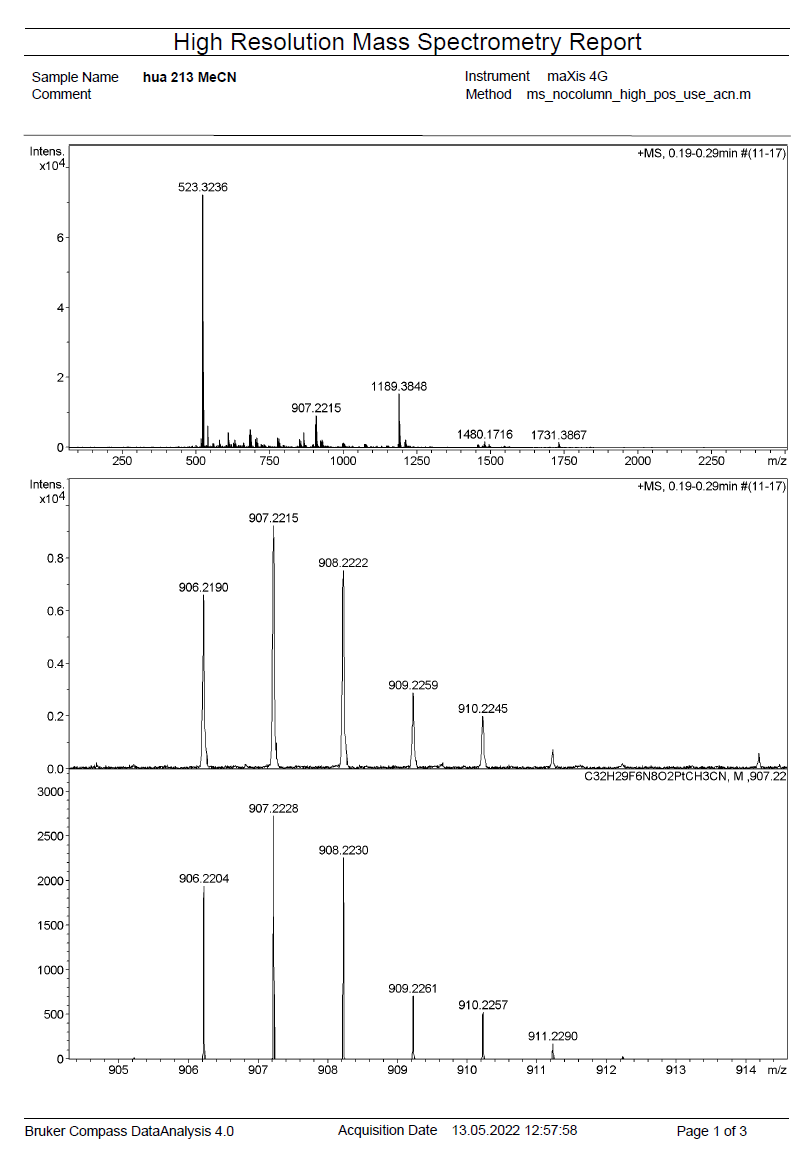


**Figure S59.** HRMS spectra of **9.**


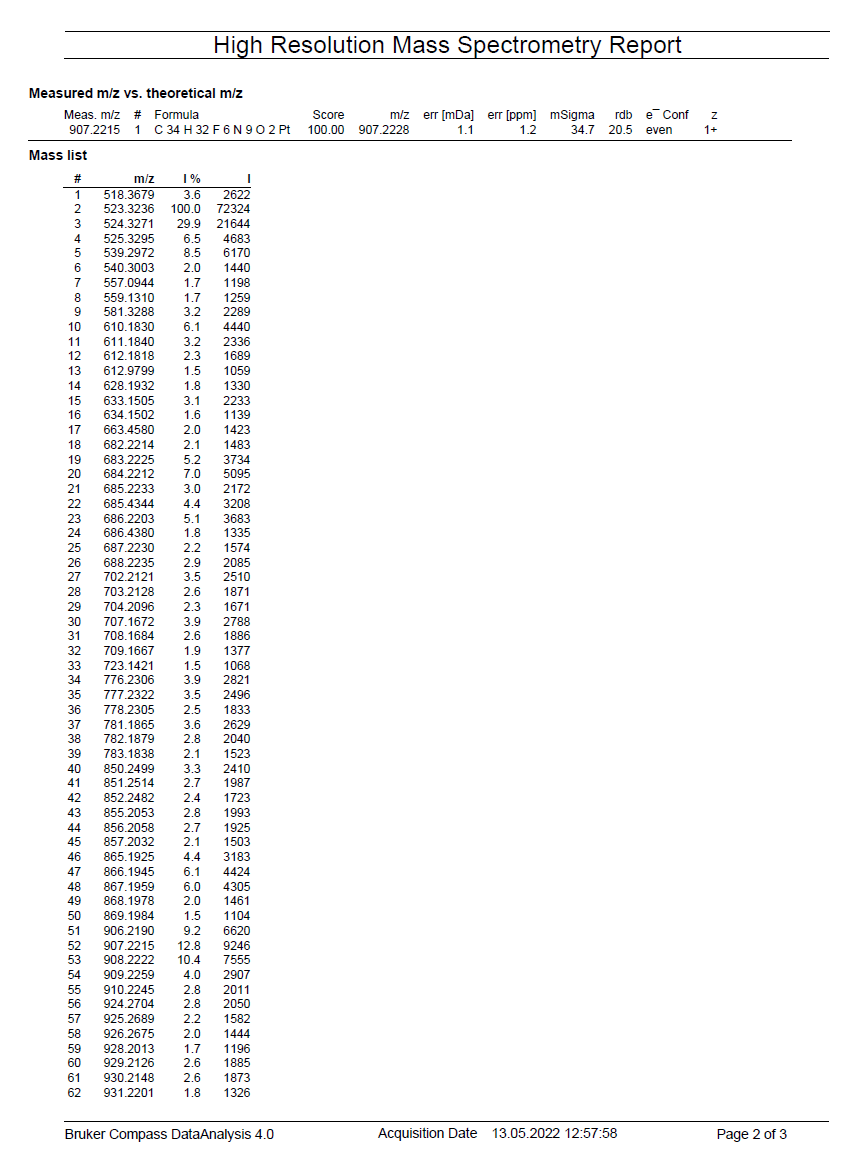


**Figure 60.** HRMS report of **9.**

#### 7.2.2 Complex **S-1**


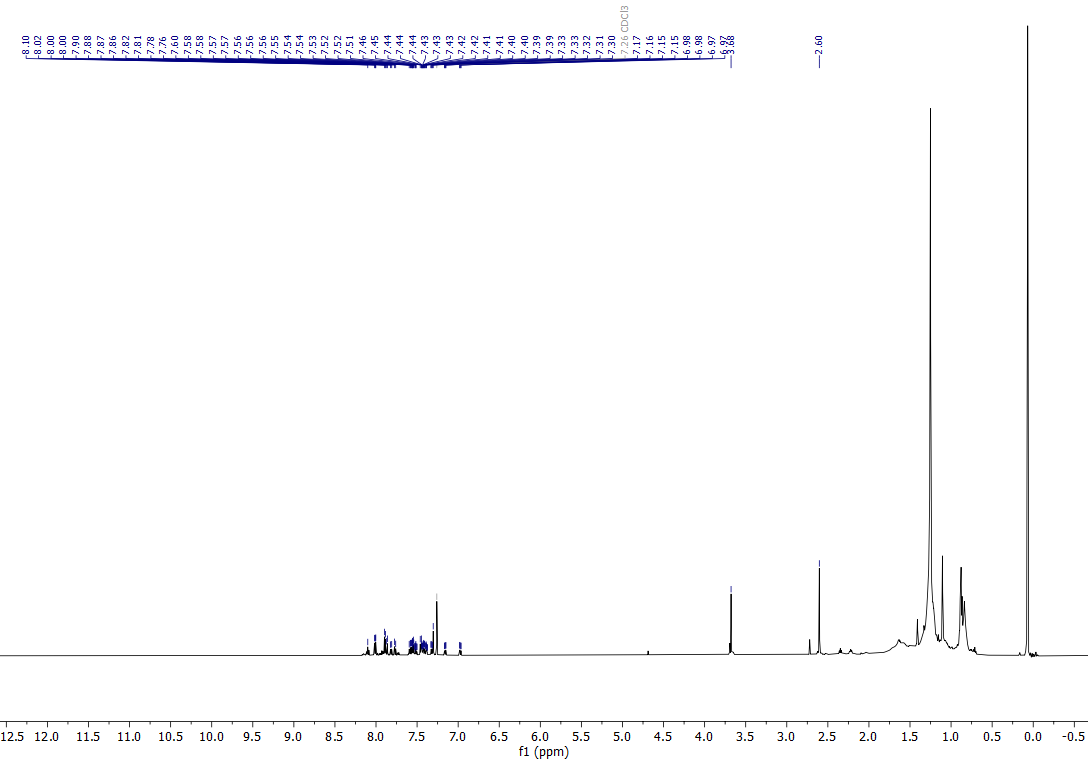


**Figure S61.** ^1^H NMR spectrum of **S-1** in CDCl_3_.


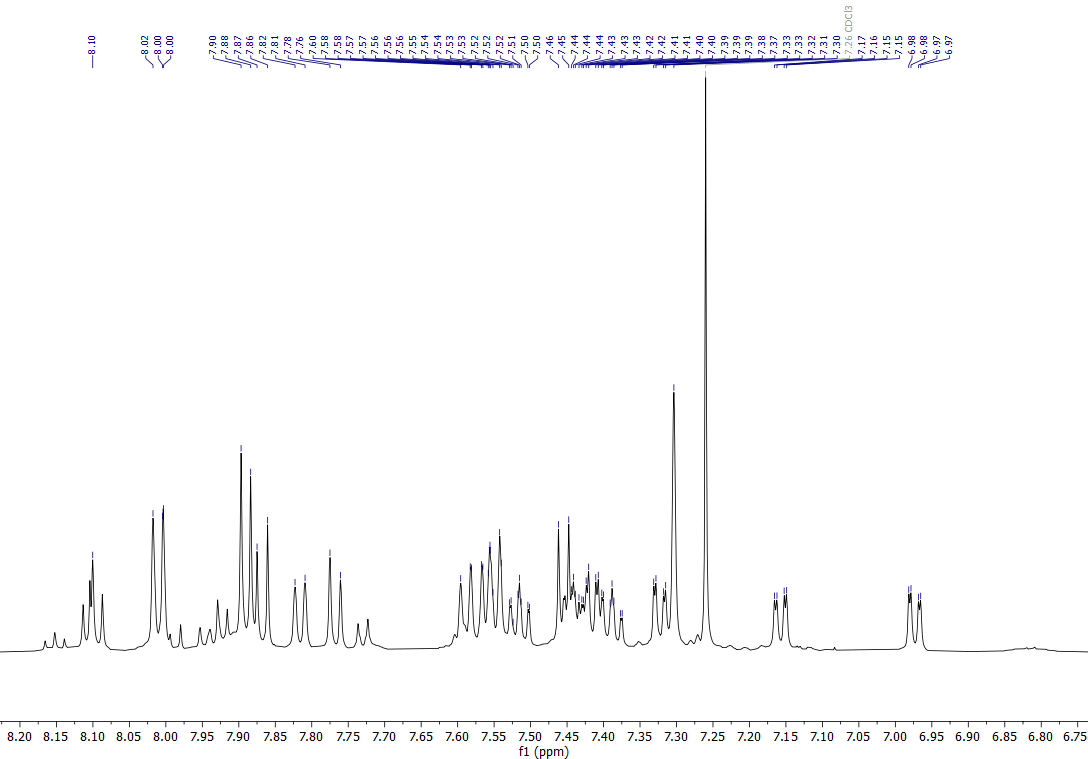


**Figure S62.** Aromatic region of the ^1^H NMR spectrum of **S-1** in CDCl_3_.


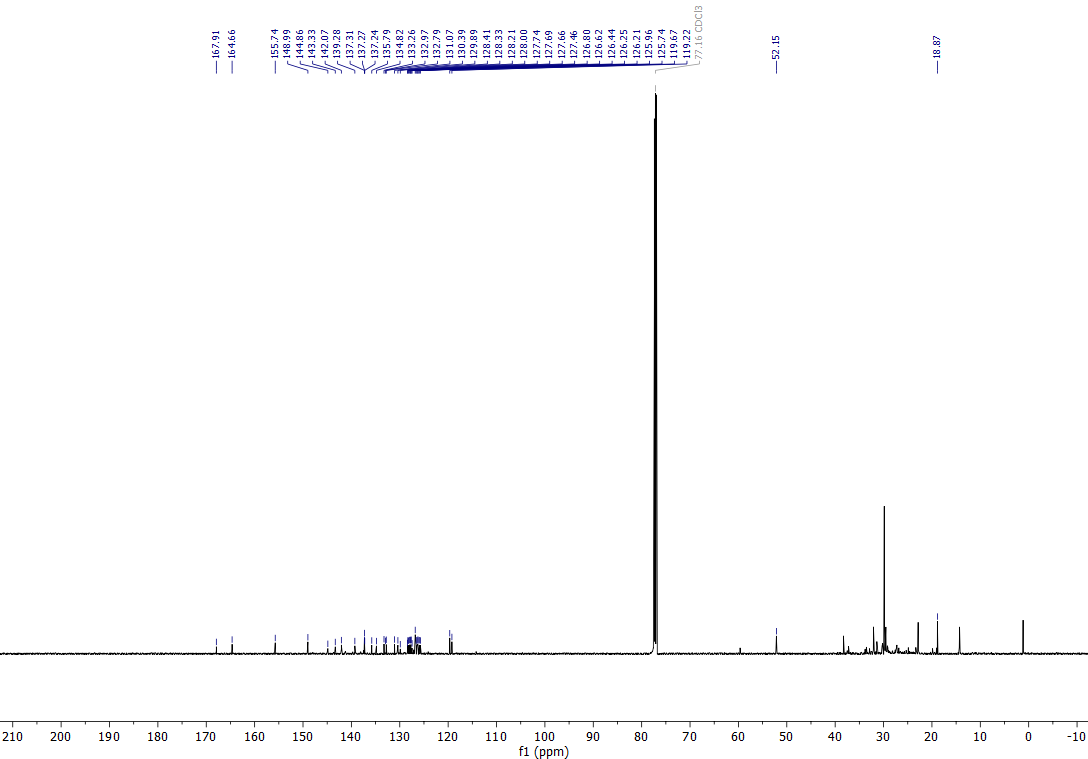


**Figure S63.** ^13^C NMR spectrum of **S-1** in CDCl_3_.


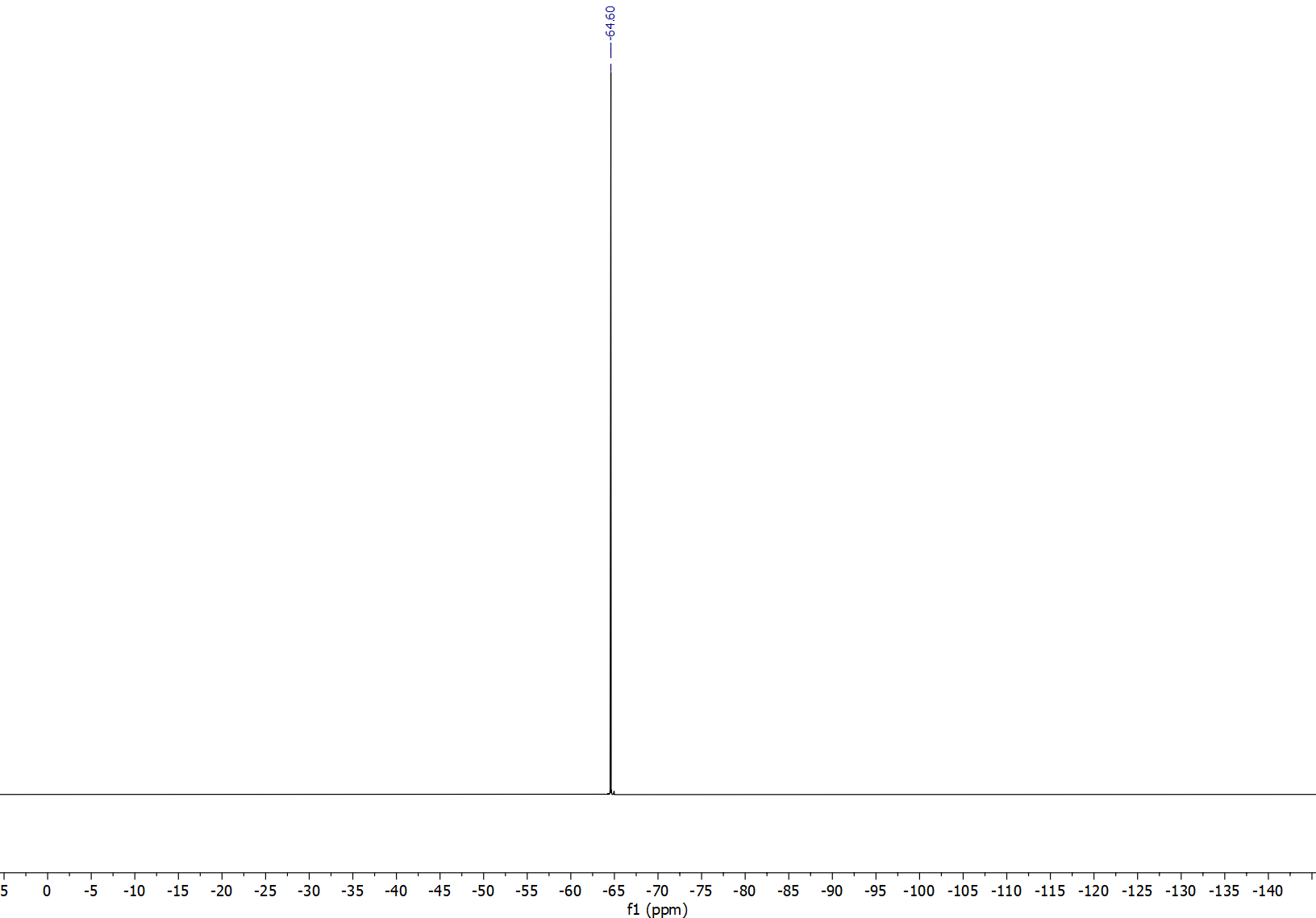


**Figure S64.** ^19^F NMR spectrum of **S-1** in CDCl_3_.


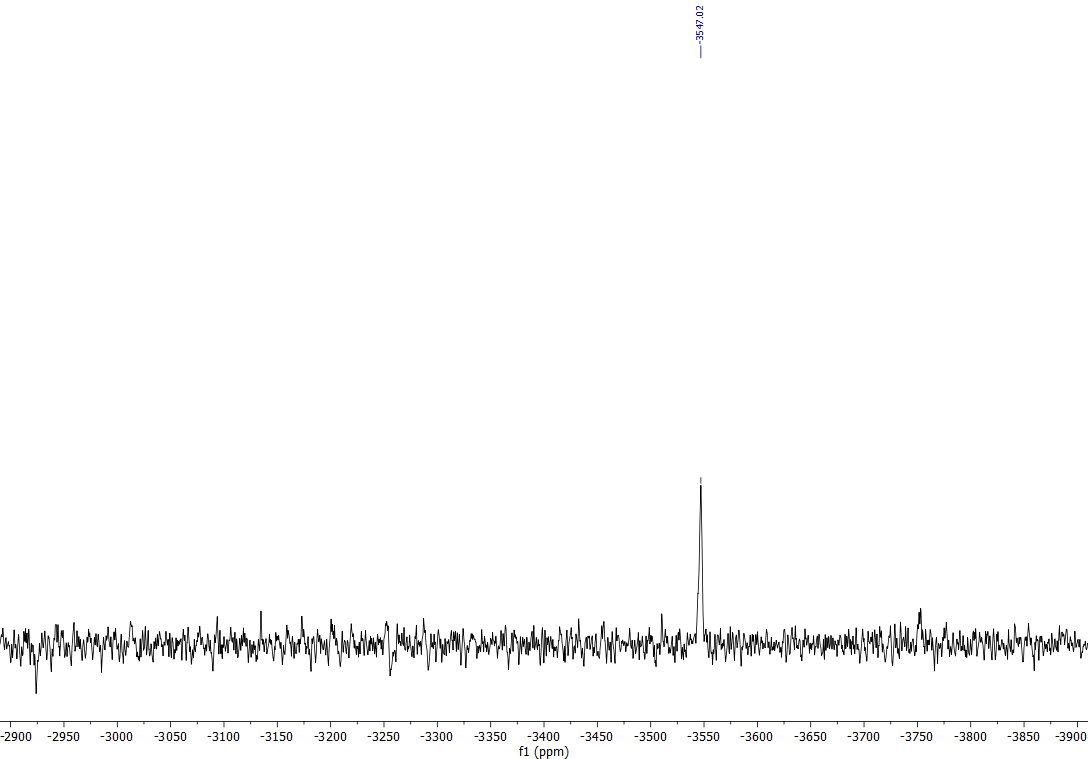


**Figure S65.** ^195^Pt NMR spectrum of **S-1** in CDCl_3_.


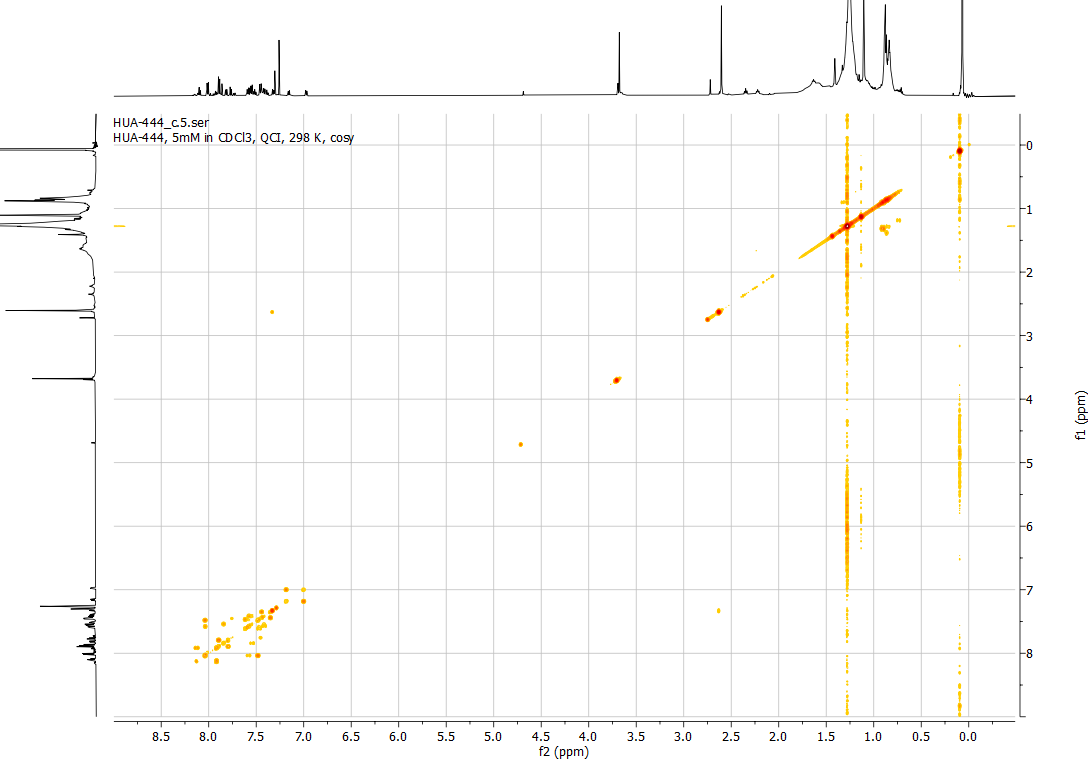


**Figure S66.** COSY spectrum of **S-1** in CDCl_3_.


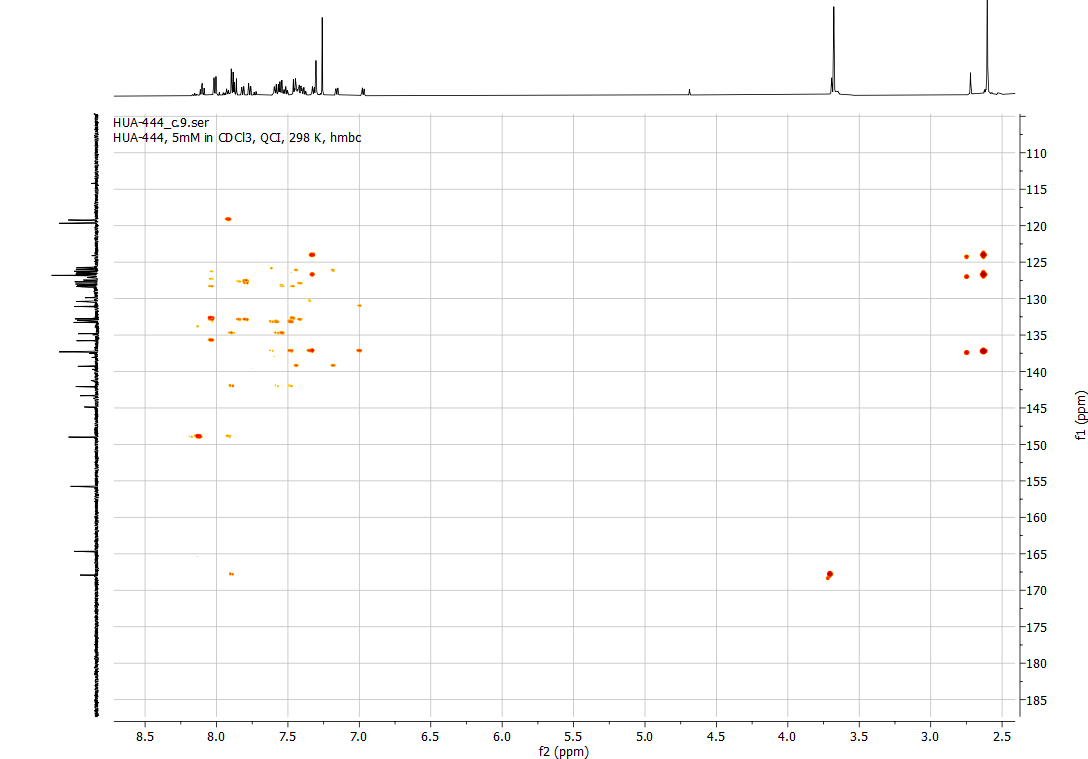


**Figure S67.** HMBC NMR spectrum of **S-1** in CDCl_3_.


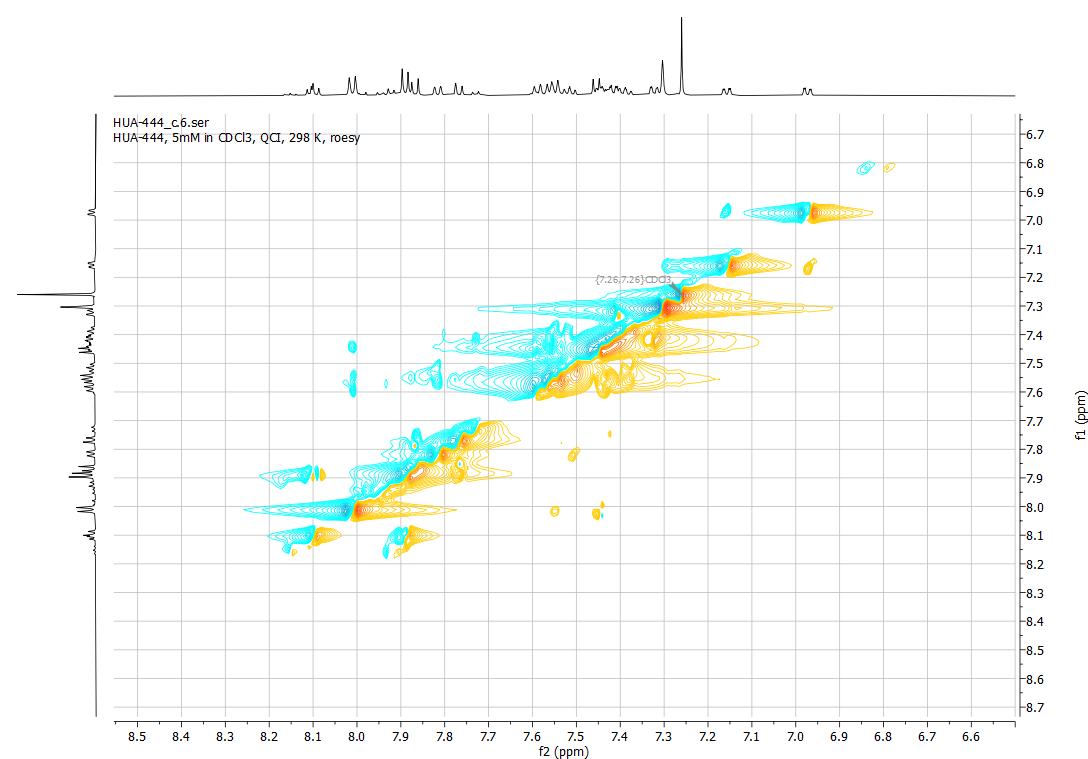


**Figure S68.** ROESY spectrum of **S-1** in CDCl_3_.


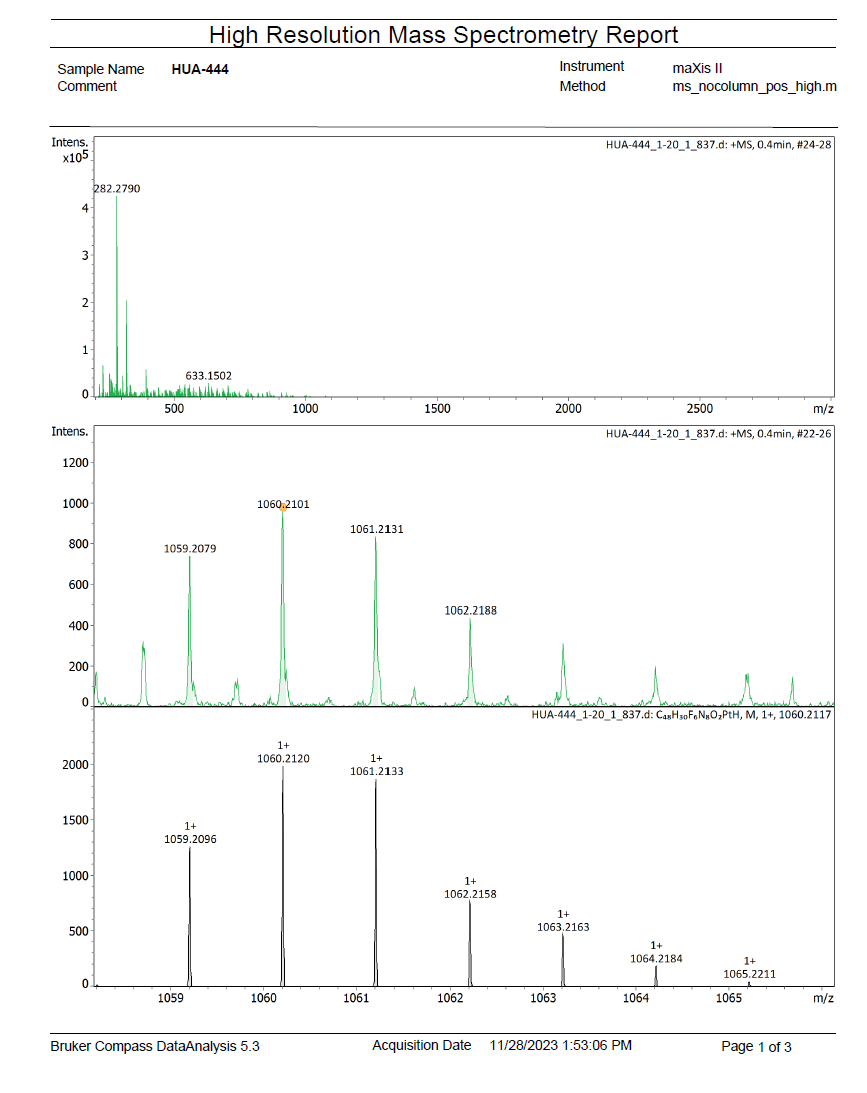


**Figure S69.** HRMS spectra of **S-1.**


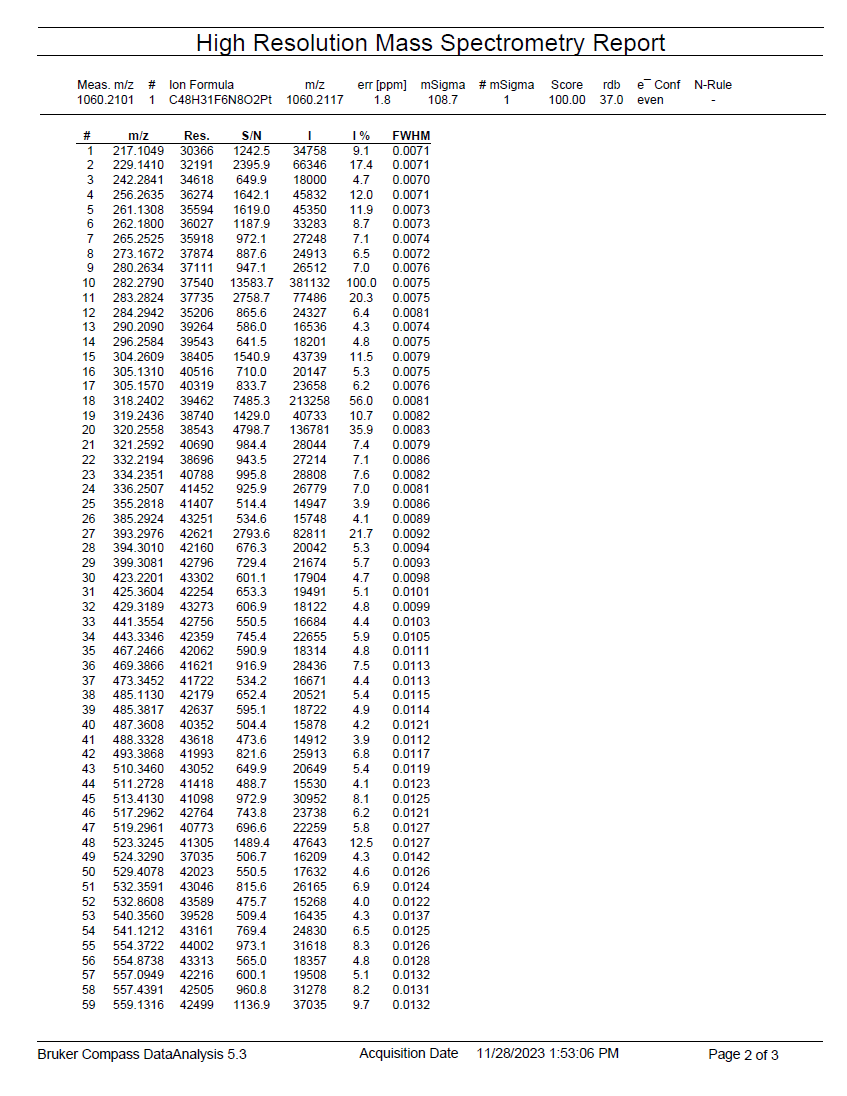


**Figure S70.** HRMS report of **S-1.**

## 8. FTIR spectra


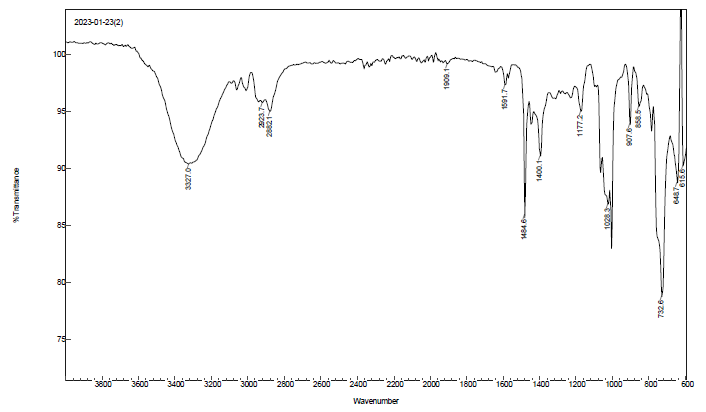


**Figure S71.** FTIR spectrum of **2**.


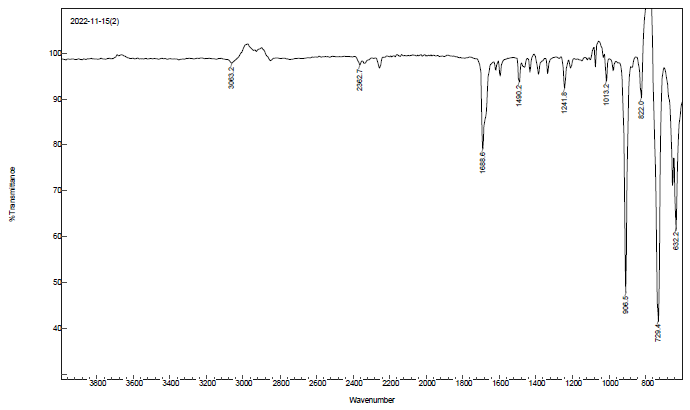


**Figure S72.** FTIR spectrum of **3**.


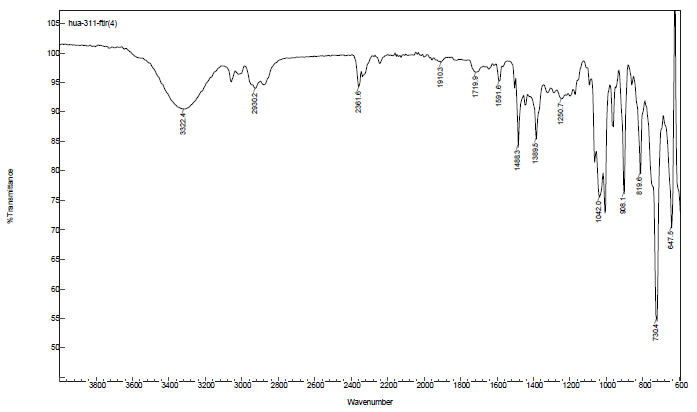


**Figure S73.** FTIR spectrum of **4**.


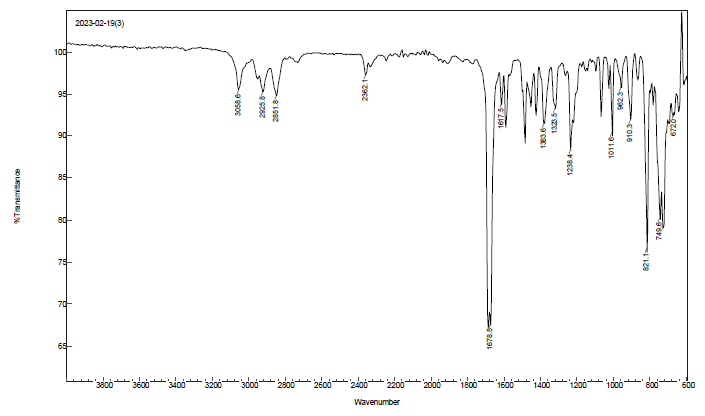


**Figure S74.** FTIR spectrum of **S-5**.


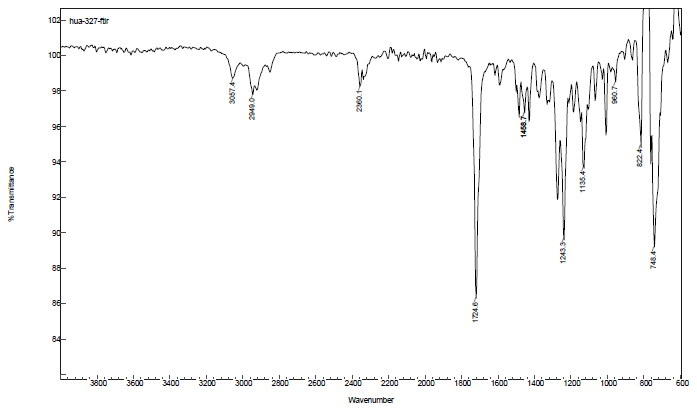


**Figure S75.** FTIR spectrum of **S-6**.


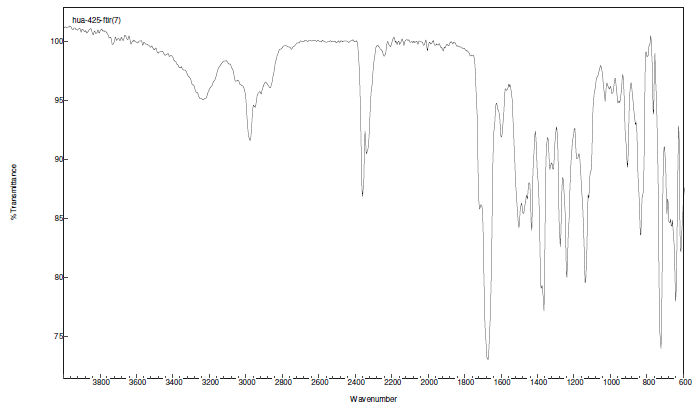


**Figure S76.** FTIR spectrum of **11**.


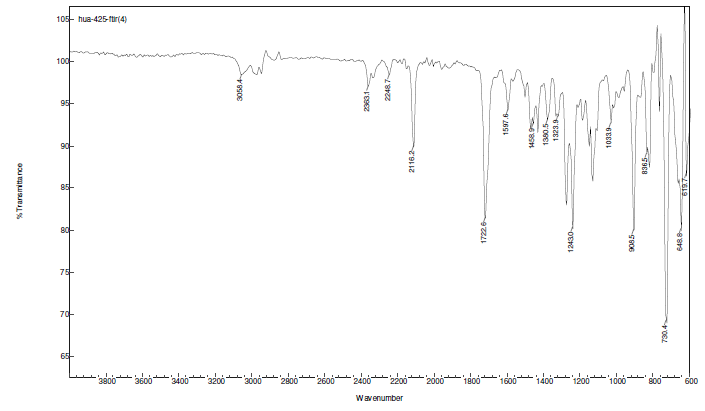


**Figure S77.** FTIR spectrum of **S-8**.

## 9. Chiral HPLC data


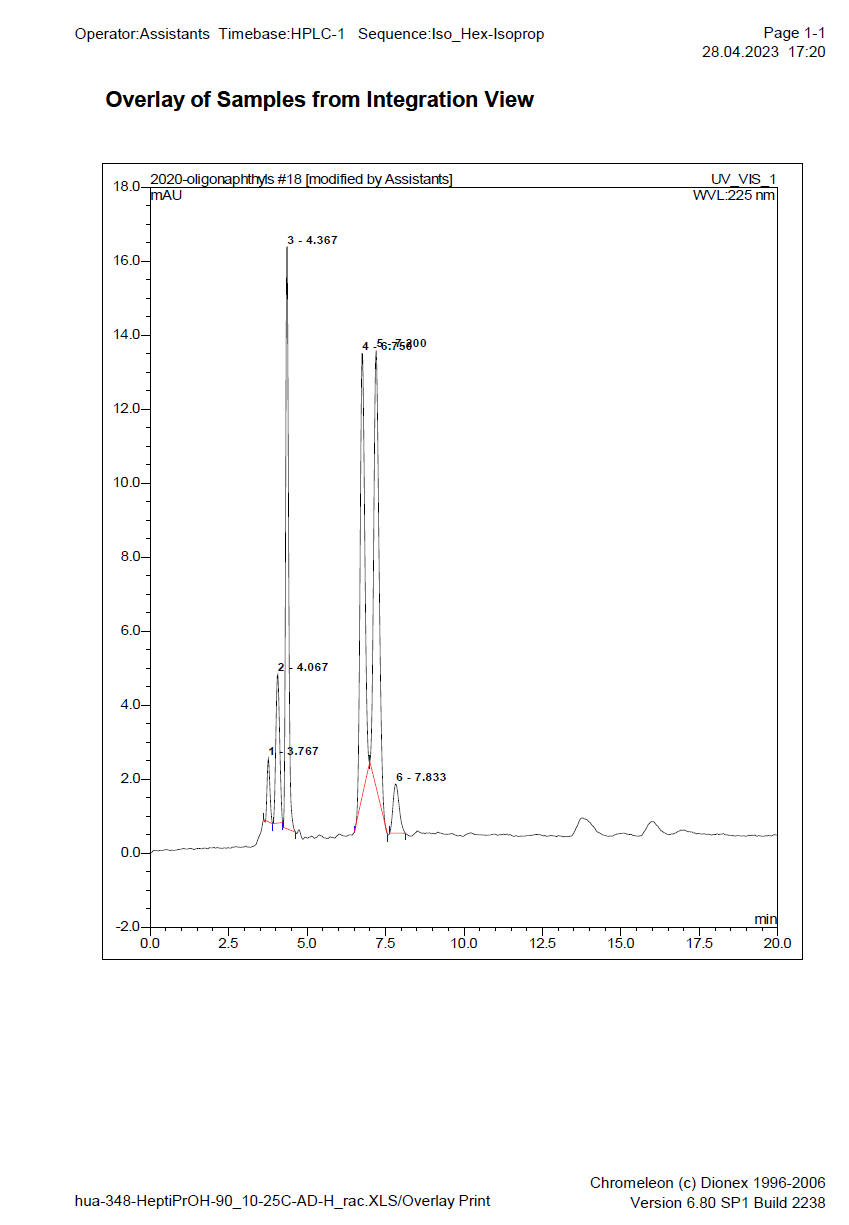


**Figure S78.** HPLC chromatogram of racemic **5.**


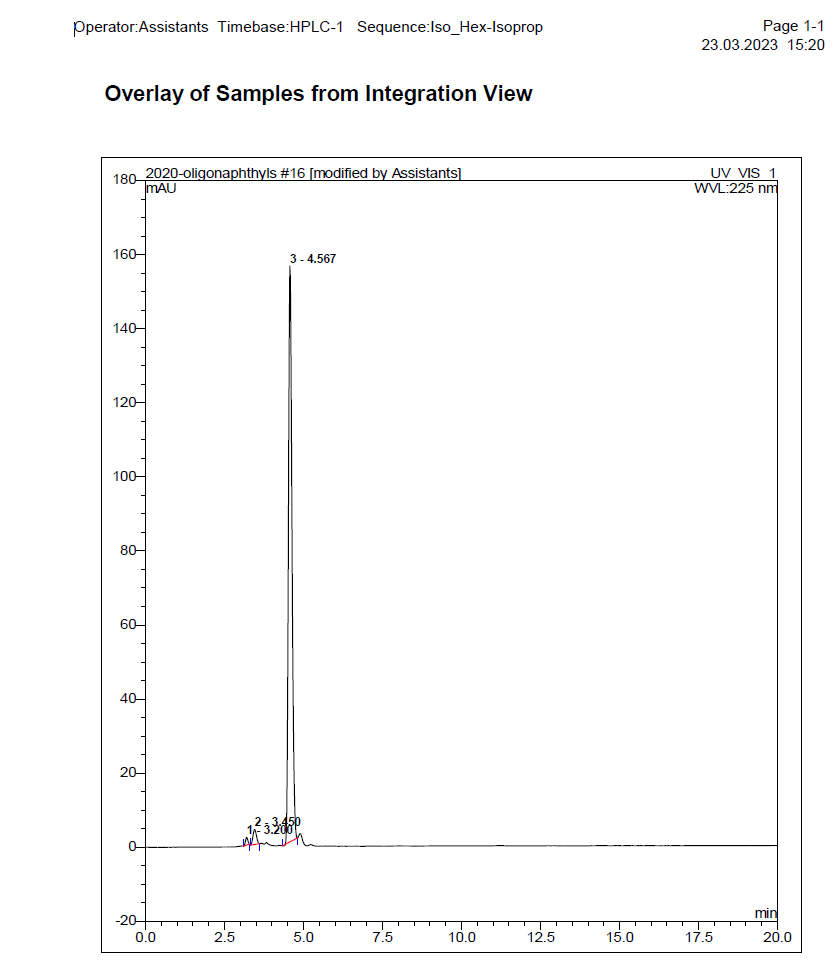


**Figure S79.** HPLC chromatogram of **S-5** using the same column and methods as in Figure S78.

## 10. X-Ray crystal structure of **10**


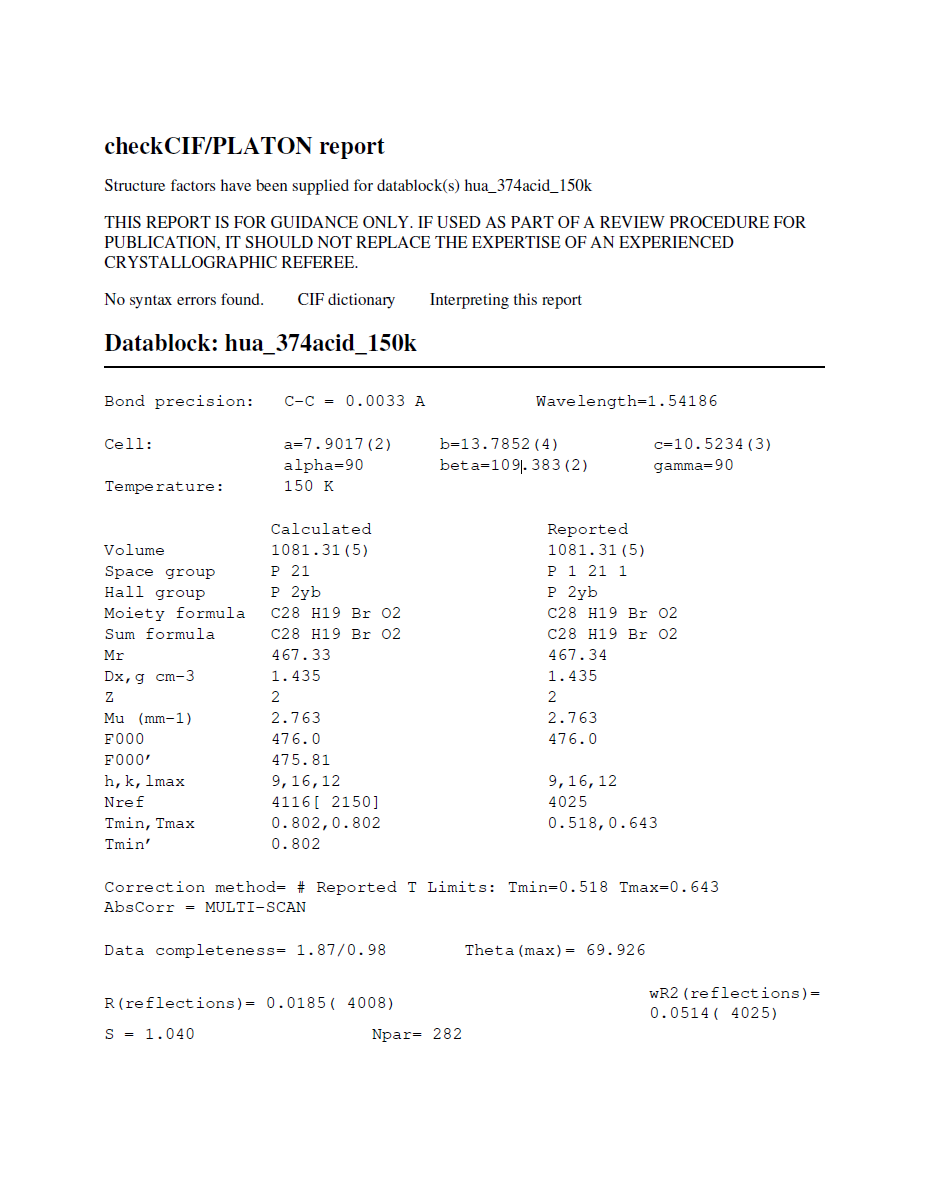


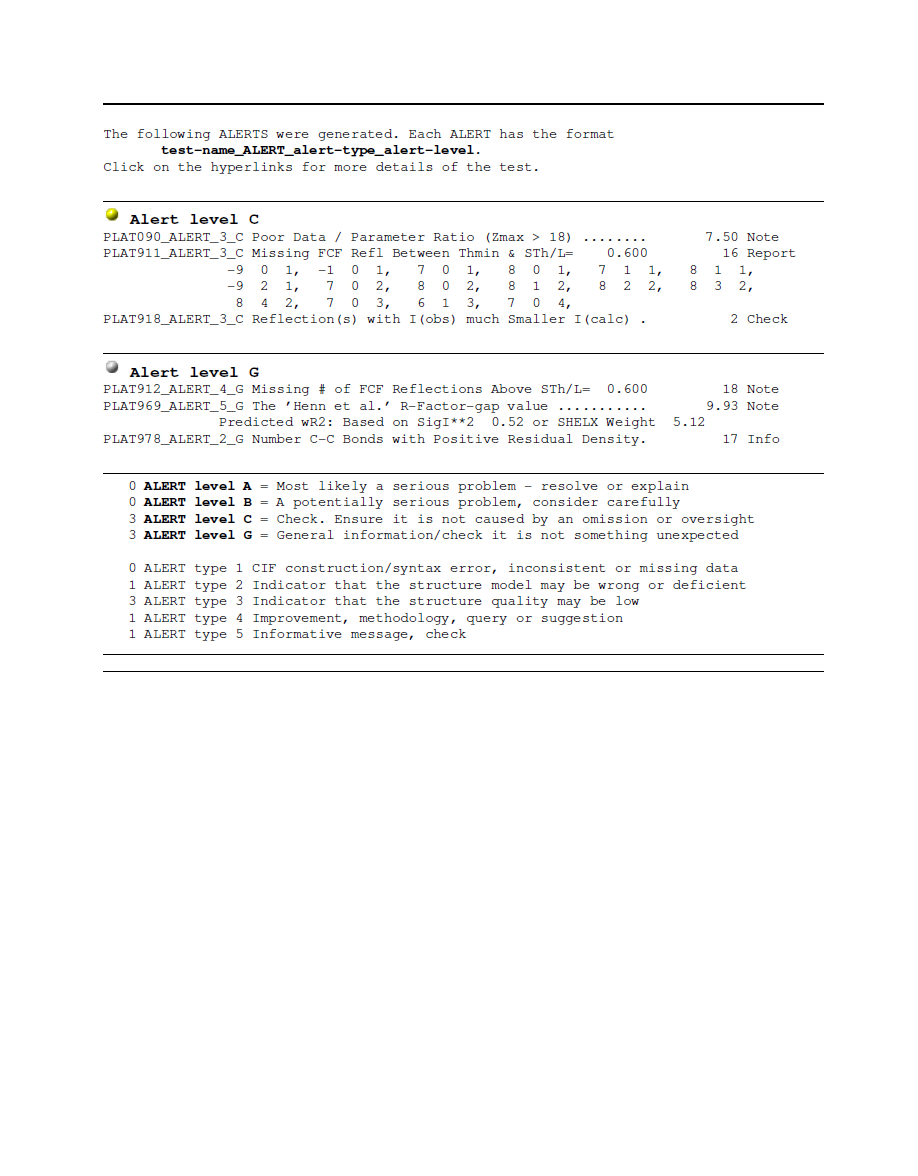


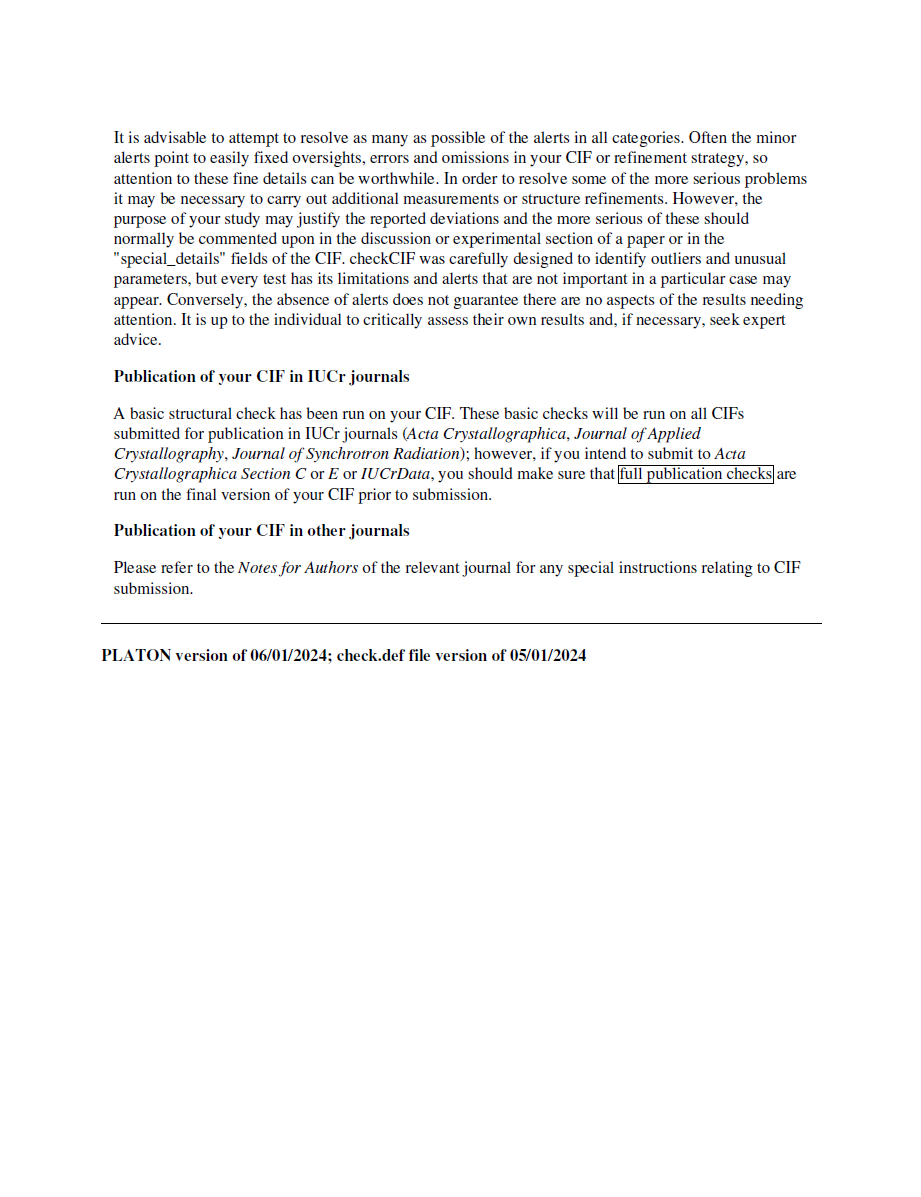


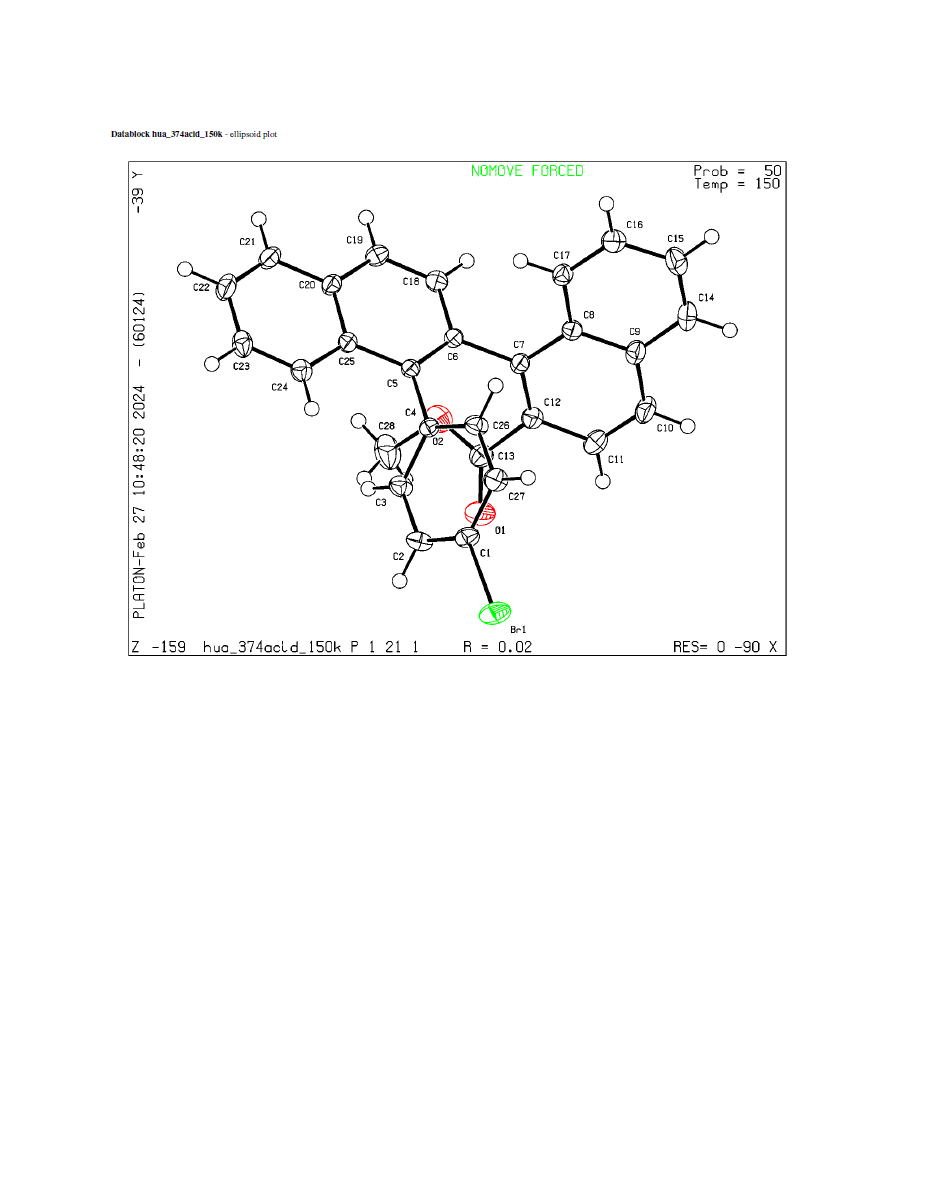


## 11. References

[1] M. Frigerio, M. Santagostino, *J. Org. Chem.* **1999**, *64*, 4537–4538.

[2] A. Krasovskiy, P. Knochel, *Synthesis* **2006**, 890–891.

[3] F. Neese, *Wiley Interdiscip. Rev. Comput. Mol. Sci.* **2012**, *2*, 73–78.

[4] F. Neese, *Wiley Interdiscip. Rev. Comput. Mol. Sci.* **2018**, *8*, e1327.

[5] F. Neese, F. Wennmohs, U. Becker, C. Riplinger, *J. Chem. Phys.* **2020**, *152*, 224108.

[6] S. Grimme, J. G. Brandenburg, C. Bannwarth, A. Hansen, *J. Chem. Phys.* **2015**, *143*, 054107.

[7] S. Grimme, S. Ehrlich, L. Goerigk, *J. Comput. Chem.* **2011**, *32*, 1456–1465.

[8] S. Grimme, J. Antony, S. Ehrlich, H. Krieg, *J. Chem. Phys.* **2010**, *132*, 154104.

[9] F. Weigend, *Phys. Chem. Chem. Phys.* **2006**, *8*, 1057–1065.

[10] D. Septiadi, A. Aliprandi, M. Mauro, L. De Cola, *RSC Adv.* **2014**, *4*, 25709–25718.

[11] L. A. Büldt, X. Guo, A. Prescimone, O. S. Wenger, *Angew. Chem. Int. Ed.* **2016**, *55*, 11247–11250.

[12] D. Lotter, A. Castrogiovanni, M. Neuburger, C. Sparr, *ACS Cent. Sci.* **2018**, *4*, 656–660.

[13] E. F. Valeev, “Libint,” can be found under https://github.com/evaleev/libint, **n.d.**

[14] G. A. Andrienko, I. N. Senchenya, A. Romanov, **n.d.**

[15] L. A. Büldt, O. S. Wenger, *Angew. Chem. Int. Ed.* **2017**, *56*, 5676–5682.

[16] S. Ossinger, A. Prescimone, D. Häussinger, O. S. Wenger, *Inorg. Chem.* **2022**, *61*, 10533–10547.

[17] D. Lotter, M. Neuburger, M. Rickhaus, D. Häussinger, C. Sparr, *Angew. Chem. Int. Ed.* **2016**, *55*, 2920–2923.

[18] S. Sinn, F. Biedermann, L. De Cola, *Chem. Eur. J.* **2017**, *23*, 1965–1971.
